# Click Heterogenization of Phosphines Furnishes Recyclable Hydroformylation Catalysts that Reproduce Homogeneous Performance

**DOI:** 10.1021/jacs.5c10989

**Published:** 2025-09-11

**Authors:** Junjun Chen, Christophe Farès, Aamir Abbas, Constanze N. Neumann

**Affiliations:** 1 28314 Max-Planck-Institut für Kohlenforschung, Kaiser-Wilhelm-Platz 1, 45470 Mülheim an der Ruhr, Germany; 2 CAS Key Laboratory of Low-Carbon Conversion Science and Engineering, Shanghai Advanced Research Institute, Chinese Academy of Sciences, Shanghai 201210, PR China; 3 University of the Chinese Academy of Sciences, Beijing 100049, PR China

## Abstract

Heterogeneous catalysts confer notable practical advantages for large-scale reactions, while homogeneous catalysts permit targeted performance optimization. A rapid and general method for the heterogenization of molecular transition-metal catalysts without loss of performance would thus permit facile translation of optimized homogeneous catalysts into practical heterogeneous catalysts. Here, we show how a wide variety of phosphines carrying anionic substituents can be charge-tethered to the walls of the spacious supercages of a metal–organic framework (MOF) to provide an adaptable heterogeneous ligand set in a single synthetic step. The addition of Co_2_(CO)_8_ to MOF-heterogenized phosphine ligands provides recyclable, heterogeneous hydroformylation catalysts that faithfully reproduce the performance of the molecular analogues in both activity and selectivity. Key to the solution-like reactivity of the click heterogenized phosphines is their high degree of mobility, which was directly demonstrated by ^31^P NMR analysis and which enables them to effectively accommodate cobalt complexes with three distinct oxidation states and coordination geometries. While the lack of directionality of the ionic interaction between the ligand and the host permits the phosphines to effectively reproduce homogeneous catalytic cycles, the strength of the ionic interaction ensures that phosphine leaching remains below 0.05 ppm.

## Graphical abstract

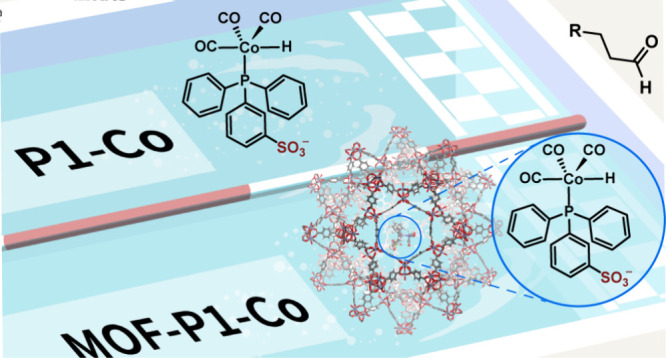

## Introduction

As the chemical industry shifts from oil-derived feedstock to waste- or renewable-derived feedstock, many traditional production processes need to be replaced or reoptimized so that a rapid pipeline for new high-performance catalysts is required.^1–3^ Given the challenges associated with the targeted optimization and characterization of heterogeneous catalysts, high-throughput optimization of a molecular catalyst followed by molecular heterogenization presents a promising approach for accelerated development of recyclable catalysts (Figure 1A).^4^ Molecular heterogenization with a wide range of different platforms, including solid surfaces,^5,6^ single atom catalysts (SACs),^7^ MOFs,^8,9^ covalent organic frameworks (COFs),^10^ porous polymers^11^ and supported ionic liquids^12^ have furnished examples of excellent catalytic performance. Nonetheless, the practical value of heterogenized molecular catalysts has frequently been limited by catalyst leaching or an unreasonable increase in the complexity of catalyst synthesis.^13,14^ The challenges associated with an economical heterogenization strategy which does not compromise catalyst performance are illustrated by the fact that multiple large-scale industrial processes still rely on homogeneous metal catalysts despite the need for laborious catalyst recovery and recycling strategies.^15–18^ Olefin hydroformylation, for example, is used to convert olefins and syngas to a mixture of terminal and internal aldehydes in the presence of homogeneous (phosphine or phosphite promoted) rhodium or cobalt catalysts on an annual scale of 10 million tons.^19^ While many examples of molecular heterogenization of rhodium hydroformylation catalysts have been reported,^20–29^ there is a dearth of examples for cobalt,^30–32^ even though recycling of the homogeneous cobalt catalyst requires a laborious sequence of steps that results in copious amounts of corrosive waste.^33,34^ A notable additional challenge for the heterogenization of the cobalt-catalyzed process is that the active catalyst is unstable in the absence of syngas, meaning a heterogeneous ligand set for a cobalt phosphine hydroformylation catalyst needs to effectively support the precatalyst in addition to the various intermediates involved in the catalytic cycle (Figure S7). Ligands in homogeneous catalysis enjoy substantial mobility which permits them to readily adapt to the coordination preferences of transition metal centers, but their immobilization on a support material commonly robs them of their translational freedom.^35,36^ The mobility restriction in solid matrices, polymers and supported ionic liquids can be strategically employed to realize unusual selectivity outcomes, increased catalyst lifetimes, or novel reaction pathways.^37–46^ But if a highly efficient homogeneous catalyst is already known or can easily be identified, no performance change is desired upon heterogenization (Figure 1B). Here we show that charge-tethering of sulfonated phosphine ligands to spacious MOF supercages gives rise to a mobile heterogeneous ligand set that is capable of adapting to the coordination needs of the metal while offering recyclability and curbing leaching. Cobalt complexes of the heterogeneous phosphine ligands faithfully reproduce the olefin hydroformylation performance of their soluble analogues, and, unlike classic heterogeneous catalysts for olefin hydroformylation,^47–56^ can be rationally optimized through changes to the phosphine ligand.^57–59^ We developed a click heterogenization strategy that tolerates a range of structurally distinct phosphines, proceeds in a single synthetic step from commercially available starting materials, and permits recovery of the MOF support at the end of life of the catalyst (Figure 1A).

**Figure 1:**
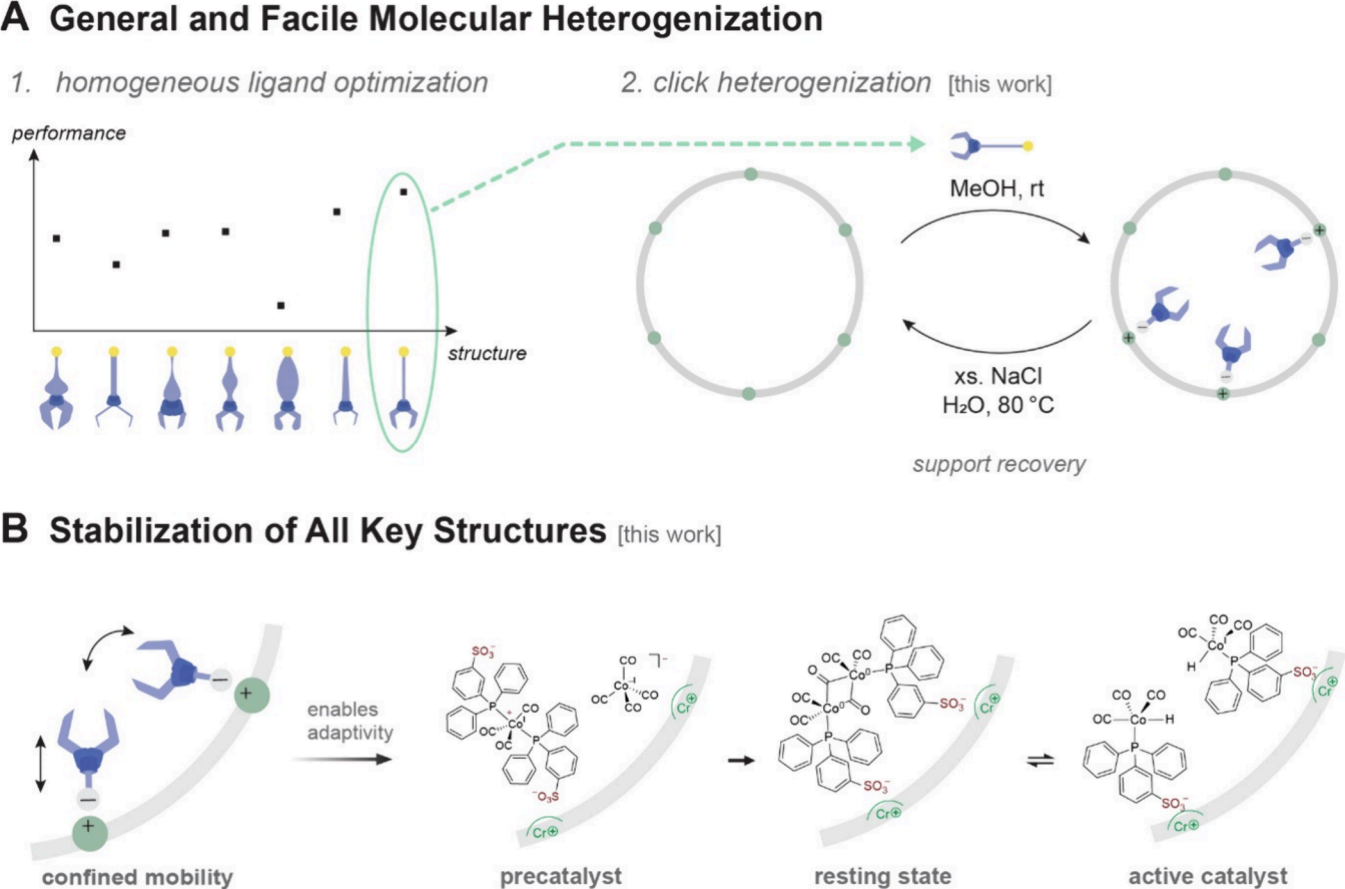
Addressing Challenges in Molecular Heterogenization. **A** High-performing phosphine ligands, identified via screening under homogeneous conditions, can be securely (but reversibly) heterogenized via simple charge-tethering. **B** The MOF provides an adaptable heterogeneous ligand set that can accommodate transition metals with a variety of oxidation states and coordination geometries, which is illustrated here for cobalt catalyzed hydroformylation.

## Results and Discussion

The MOF Cr-MIL-101, developed by the Ferey group,^60^ was chosen as a support matrix because of its high chemical stability, cheap linker, facile preparation on gram-scale, and its cage-type pore system. The presence of two types of mesoporous cages (2.9 and 3.4 nm in diameter) that are accessed via pentagonal or hexagonal windows with diameters of 1.2 and 1.6 nm provides spacious cages for catalytic transformations and permits facile ingress and egress of even large substrate molecules. Furthermore, prior work from the Sanford,^61^ Dincă ^62,63^ and Chmielewski^64^ laboratories demonstrated anionic and cationic complexes could be securely anchored within the pores of Cr-MIL-101 via ion-pair interactions with the MOF nodes or linkers.^62,63^ Each node of MIL-101 consists of three chromium centers, two of which carry a neutral water ligand, while one of them carries an X-type ligand such as fluoride, chloride or hydroxide. Our click heterogenization approach for phosphine ligands relies on the preparation of MIL-101 with chloride-terminated nodes (Figure S1) which is then exposed to phosphine ligands carrying a sulfonate group. Salt metathesis gives rise to the charge-tethered phosphine in the MOF pore in addition to NaCl, which is removed by simple washing of the MOF-P powder (Figure 2A).^61,65,66^ Notably, a wide range of sulfonated phosphines are commercially available since they are commonly employed as water-soluble ligand derivatives for biphasic reactions. When we tested the phosphine heterogenization protocol developed with **P1** on a range of structurally distinct phosphine ligands (Figure 2B, Figure S3), all of them could be heterogenized effectively without the need to reoptimize the reaction conditions. The click heterogenization protocol is applicable to aliphatic and aromatic phosphines, bidentate phosphines, phosphines carrying varying numbers of sulfonate groups, and even phosphines carrying a positive charge in addition to a negatively charged substituent. A theoretical maximum of 10 phosphine ligands can be accommodated in the smaller cage and 14 phosphine ligands in the larger cage of the MOF via formation of an ion pair with the MOF node. Experimentally, a range of loadings of **P1** (up to a maximum of 6.2 phosphines per cage) could be installed in MIL-101 without any noticeable change in the crystallinity (Figure 2C). Due to the extremely large diameter of the MOF cages, the incorporation of an average of 6.2 **P1** per cage proceeds with only a 21% reduction in the available pore volume (Figure 2E). Argon adsorption isotherms confirmed that all MOF-*x* **P1** samples (*x* = 2.0 – 6.2) exhibited type IV isotherms (Figure 2D), and the calculated pore size distribution (Figure 2E) confirmed the presence of the two types of mesopores expected for MIL-101. The sizes of both pores decreased smoothly with the introduction of charge-tethered phosphine ligands, which indicates that phosphines were distributed over mesopores throughout the MOF rather than fully occupying a subsection of pores and leaving other pores vacant. However, the larger pores showed a more significant decrease in diameter for smaller numbers of phosphine ligands, while the majority of the size reduction observed for the smaller cage only became apparent with high phosphine occupancy (Figure 2E). The disparate response to phosphine content suggests that the ligands preferentially enter the larger pores and that the majority of the difference between MOF-4.3**P1** and MOF-6.2**P1** lies in the degree of occupancy of the smaller cages.

**Figure 2:**
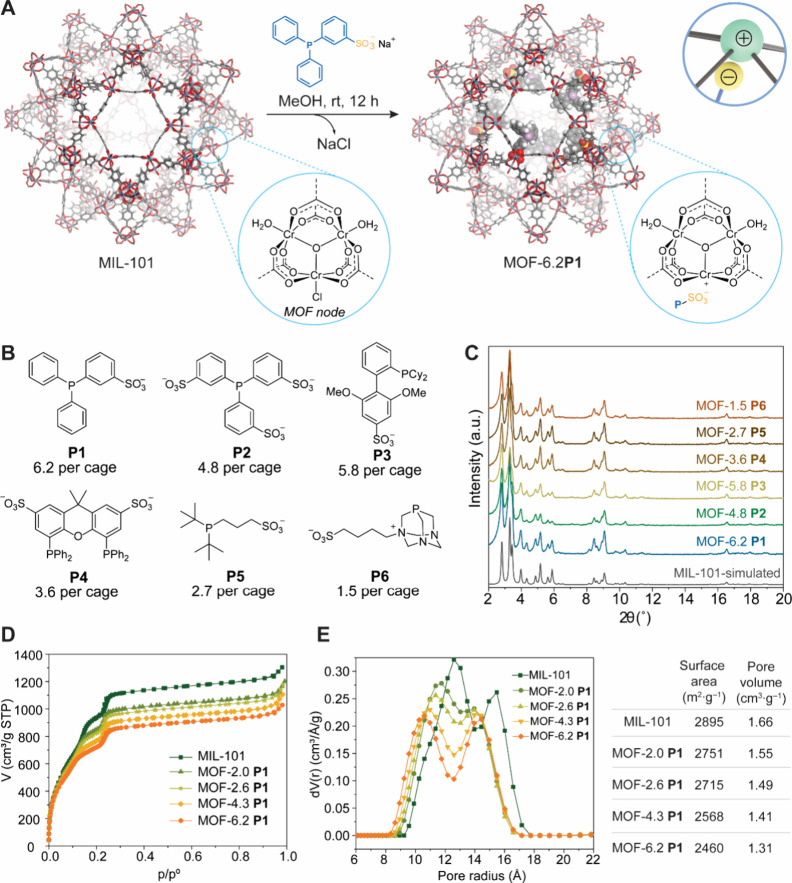
Click Heterogenization of Phosphine Ligands. **A** Synthetic procedure for the preparation of MOF-*x* **P**, where *x* is the average number of ligands **P** per MOF cage as determined by ICP-OES analysis. **B** Substrate scope of phosphines for MOF-*x* **P** synthesis. **C** Comparison of PXRD patterns of MOF-*x* **P**. Comparison of Ar adsorption isotherms (**D**) and pore size distributions (**E**) of MOF-*x* **P1** with different phosphine loadings.

After we had established that a wide range of phosphines could efficiently be installed within the mesoporous cages of MIL-101, we turned our attention to establishing how much mobility the charge-tethered phosphines enjoy within the mesopores. Solid-state NMR (SS-NMR) spectroscopy is a powerful technique for the analysis of dynamics in MOFs which is commonly applied to the study of guest diffusion, the rotation of linkers, and the breathing behavior of the framework.^67^ Here we relied on magic angle spinning (MAS) ^31^P SS-NMR spectroscopy to compare the relative freedom of motion enjoyed by the sodium salt of **P1** (Na-**P1**) and **P1** that is charge-tethered to a wall of a vacant or a solvated MOF pore. The solid-state ^31^P spectrum of Na-**P1** exhibited a resonance at δ = – 4.5 ppm in addition to small spinning side bands (Figure 3A). For **P1** tethered to desolvated MOF pores, significant signal broadening (fwhm = 2155 Hz) and multiple pronounced spinning side bands were observed due to a larger degree of bonding heterogeneity within the MOF pore compared to the crystal lattice of Na-**P1**. To determine the effect of pore solvation on phosphine mobility, ^31^P NMR spectra were collected after the sample in the NMR rotor was wetted with different solvents. Despite the fact that the added solvents had only modest polarity, the appearance of a sharp signal was observed in all three cases, alongside a residual contribution of the broadened signal with spinning side bands that was also observed for the dry MOF (Figure 3A).

**Figure 3:**
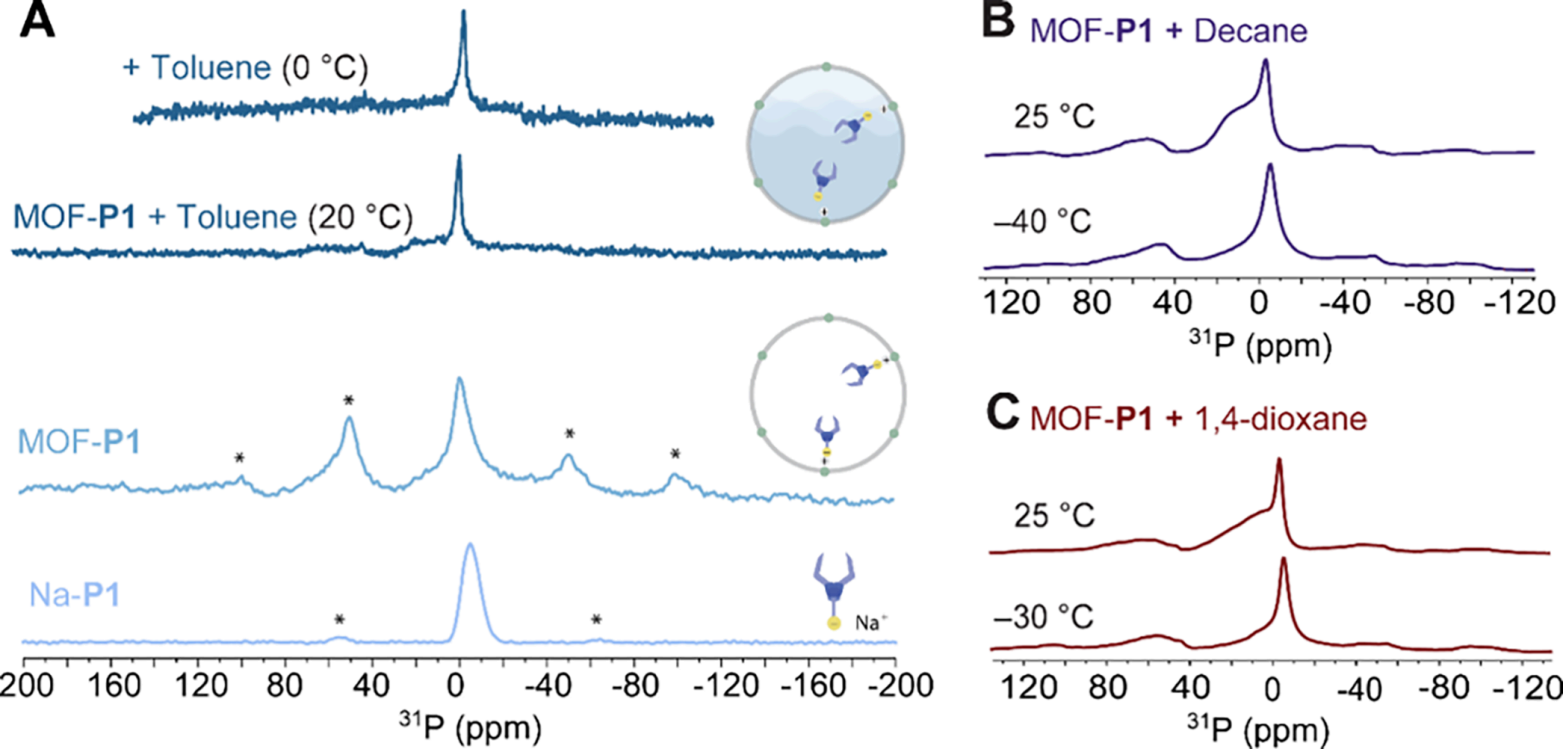
Mobility Assessment by ^31^P SS-NMR Spectroscopy. **A** Comparison of ^31^P SS-NMR spectra of Na-**P1** and MOF-**P1** in the presence and absence of solvent in the MOF pore. Variable-temperature ^31^P SS-NMR spectra of MOF-**P1** with *n*-decane (**B**) or 1,4-dioxane (**C**) solvated pores. Asterisks mark spinning side bands. All spectra were measured with a MASr = 10 kHz at 20 °C unless labeled otherwise.

We attribute the presence of a residual broad signal to limited filling of the large mesoporous cages with apolar guest molecules at elevated temperatures. In fact, the intensity of the broad signal decreased both with solvent polarity from *n*-decane (ε = 1.99; Figure 3B) to 1,4-dioxane (ε = 2.25; Figure 3C) and toluene (ε = 2.38; Figure 3A) as well as with decreasing measurement temperature (Figure 3, Figure S35). Notably, ^31^P NMR data collected at 0 °C for a MOF-**P1** sample with toluene solvated pores showed a complete disappearance of the spinning side bands, which indicates orientational time-averaging of the chemical shift anisotropy due to loss of order. In contrast to ligands introduced into MOF pores via click heterogenization, the mobility of linkers attached to secondary building units (SBUs) via dative bonds has been repeatedly observed to decrease upon addition of solvent.^68–70^ Seminal studies by Blümel and co-workers previously established a clear correlation between a solvent-induced decrease in the ^31^P NMR line widths and the mobility enjoyed by phosphine ligands heterogenized on silica surfaces.^71–73^ But while metal centers supported by mobile phosphine ligands bound to silica were found to aggregate into nanoparticles during reductive transformations, MOF-**P1** provides a mobile phosphine ligand set that is able to stabilize isolated metal centers even under high pressures of H_2_.^73^ The appearance of a single sharp ^31^P resonance for MOF-**P1** in the presence of toluene also provides convincing evidence of a single chemical environment, and thus a high degree of homogeneity among the heterogeneous ligands.

After we had confirmed that charge-tethered phosphines within a MIL-101 framework constitute a mobile heterogeneous ligand set, we investigated their ability to form coordination complexes with Co_2_(CO)_8_, the cobalt precursor for industrial cobalt hydroformylation catalysts. Simple addition of a THF solution of Co_2_(CO)_8_ to MOF-**P1** at room temperature led to the formation of MOF-**P1**-Co, which contains [Co^I^(CO)_3_(**P1**)_2_]^−^ and [Co^–I^(CO)_4_]^−^ anions that are charge-tethered to the cationic MOF nodes. The observed outcome parallels reactivity in solution, where addition of phosphine ligands to Co_2_(CO)_8_ has been reported to result in the formation of [Co­(CO)_3_(PPh_3_)_2_]^+^[Co­(CO)_4_]^−^.^74–81^ Since no solid-state structure of [Co­(CO)_3_(PPh_3_)_2_]^+^[Co­(CO)_4_]^−^ (**1**) had previously been reported, we collected single crystal X-ray data (Figure 4A, Table S5, S6) to provide unambiguous confirmation of the structure of the molecular model complex used to assist in the structure assignment of MOF-**P1**-Co. Collection of ^59^Co MAS SS-NMR data initially only furnished a single peak at – 3001 ppm (Figure 4B, Figure S37), which was in excellent agreement with the theoretically predicted (Figure 4B) and the previously reported chemical shift of the Co­(CO)_4_ anion.^82^ Compared to the tetrahedral Co­(CO)_4_ anion, the trigonal bipyramidal Co­(CO)_3_(PPh_3_)_2_ cation contains a ^59^Co nucleus in a significantly more anisotropic environment, which leads to a large quadrupolar coupling constant that stretches the NMR signal over hundreds of MHz. After prolonged acquisition time, however, a faint signal could be detected that is in good agreement with the theoretical prediction obtained by optimization of the experimental structure of **1** in the CASTEP program and prediction of the NMR parameters using the PBE functional (Figure 4B, 4C). In both the solution and solid-state ^31^P NMR spectra of **1**, only a single resonance was observed at 58 ppm (Figure 4E), which confirmed the presence of the single phosphorus environment expected for the target structure.^75,83^ Notably, both the ^31^P (Figure 4E) and ^59^Co SS-NMR data (Figure 4D) of MOF-**P1**-Co were in excellent agreement with the data obtained for molecular model complex **1**, albeit the lower cobalt content of MOF-**P1**-Co precluded direct observation of the ^59^Co resonance of the Co^I^ center. To further confirm that the pore-confined cobalt complex is structurally analogous to **1**, we collected ATR-IR data with particular focus on the carbonyl stretching region that acts as a sensitive reporter of the geometry (Figure 4F). In addition to the CO stretches at 2006 and 1890 cm^–1^ expected based on comparison with theoretical models and **1**, we observed an additional signal at 1962 cm^–1^ for MOF-**P1**-Co. We attribute the additional signal to the presence of minor amounts of [Co­(CO)_3_ **P1**]_2_ based on comparison with the predicted stretching frequencies of molecular model **2** (Figure 4F) and prior literature reports.^84^ Notably, the formation **2** has previously been reported to occur upon heating of **1** in toluene.^85,86^ A slight desymmetrization and a minor change in the position of the CO stretches of [Co­(CO)_4_]^−^ in MOF-**P1**-Co compared with **1** could be attributed to interaction of the [Co­(CO)_4_]^−^ anion with positively charged Cr^III^ centers on the MOF node by comparison with an authentic sample of MOF-Co­(CO)_4_ (Figure 4F).^62,63^

**Figure 4:**
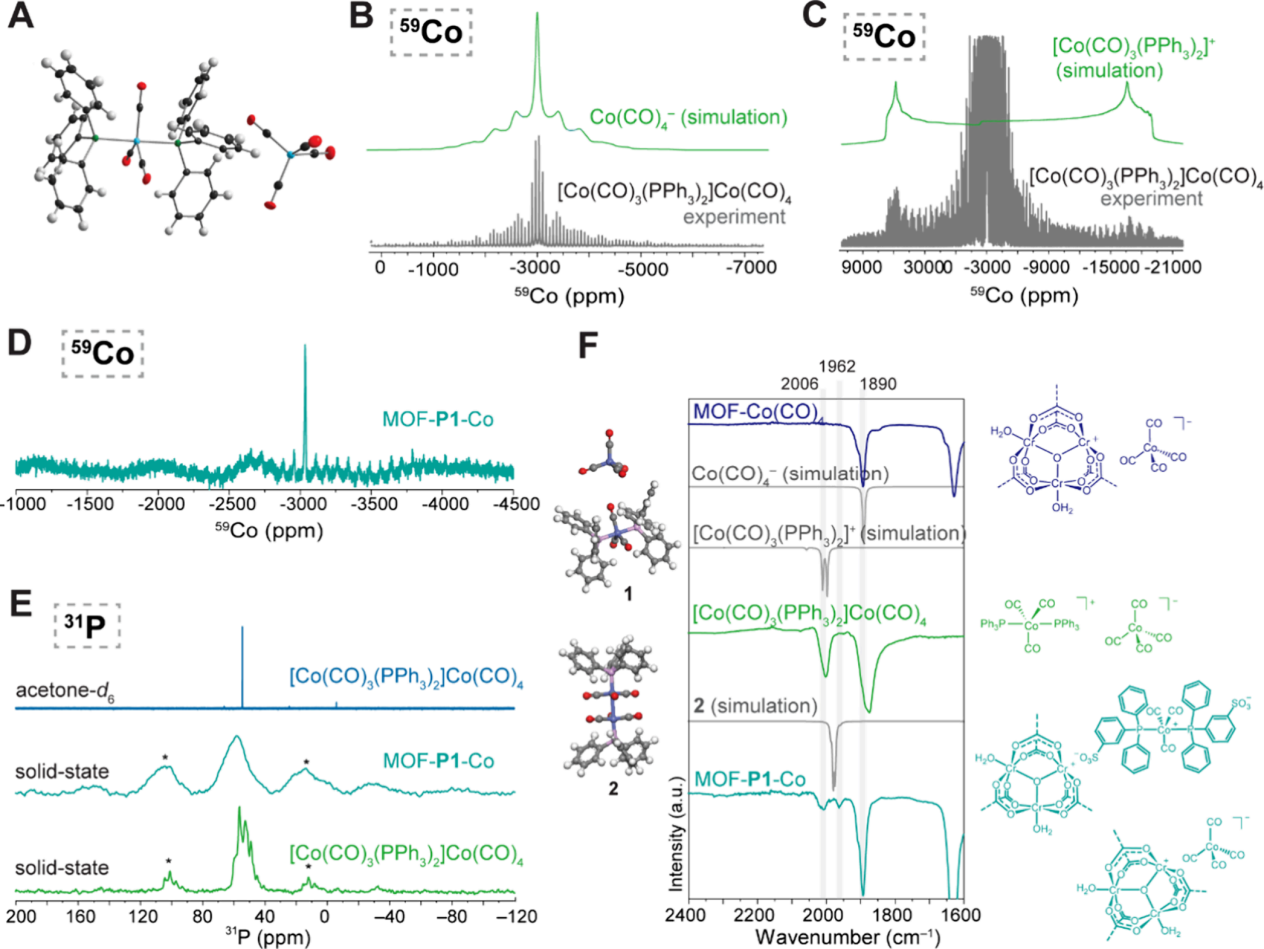
Structure of the MOF-**P1**-Co Precatalyst. X-ray structure (**A**) and ^59^Co solid-state NMR acquired using a WURST-CPMG pulse sequence (**B**, **C**) of molecular model complex [Co­(CO)_3_(PPh_3_)_2_]^+^[Co­(CO)_4_]^−^. **D** Static WURST-CPMG ^59^Co solid-state NMR of the MOF-**P1**-Co precatalyst. **E** Comparison of the solution and solid-state ^31^P NMR spectrum of [Co­(CO)_3_(PPh_3_)_2_]^+^[Co­(CO)_4_]^−^ with the ^31^P SS-NMR spectrum of the MOF-**P1**-Co precatalyst (MASr = 10 kHz for solid-state measurements). **F** Comparison of the ATR-IR spectrum of different complexes between experiment and simulation.

With the targeted precatalyst for olefin hydroformylation in hand, we carried out a brief optimization of the reaction conditions (Figure S46 and S47) and selected a temperature of 170 °C, a pressure of 40 bar, and a H_2_ : CO ratio of 1:1 for further experiments.^59^ Interestingly, a comparison of different phosphine loadings demonstrated that MOF-based catalysts containing a higher phosphine loading furnished improved performance (Figure S48), which has also been observed for a MOF-based rhodium hydroformylation catalyst reported by Canivet and co-workers.^27^ Optimal performance was reached for an average pore occupancy of 6.2 **P1** – 6.4 **P1** per MOF cage. We then explored a range of different phosphine to cobalt ratios in the hydroformylation of 1-hexene with a loading of 0.5 mol % cobalt (Figure S49). While a marginally higher conversion was observed for higher cobalt loadings, **P1**/Co ratios of 0.9 – 1 were selected for further experiments to minimize the risk of cobalt leaching during recycling. In homogeneous catalysis, higher activity is likewise observed for the unligated cobalt catalyst, but hydroformylation is carried out in the presence of phosphine ligand in the Shell process to maximize catalyst lifetime and permit adjustment of the product distribution by the phosphine ligand.^57,82^ Comparison between the homogeneous and heterogeneous cobalt catalyzed hydroformylation with **P1** as well as **P2** demonstrated that close to identical conversions and selectivities were obtained for both ligand structures (Figure 5A). It should be noted that olefin isomerization is extremely rapid under both homogeneous and heterogeneous reaction conditions and all hexene isomers participate in subsequent hydroformylation (Figure 5A and Table S7). The olefin isomers thus function as substrates and not as products of the hydroformylation reaction and are gradually converted to hydroformylation products as the reaction time is extended. Data shown in Figure 5A and 5C was deliberately collected at partial conversion to permit comparison of catalytic activity upon heterogenization and upon recycling.^87,88^ Comparative data collected for reaction times of 1 h, 2 h or 3 h (Figure S50) show that the relative reaction rates of MOF-**P1**-Co and its homogeneous analogue are close to identical throughout the course of the reaction.

**Figure 5:**
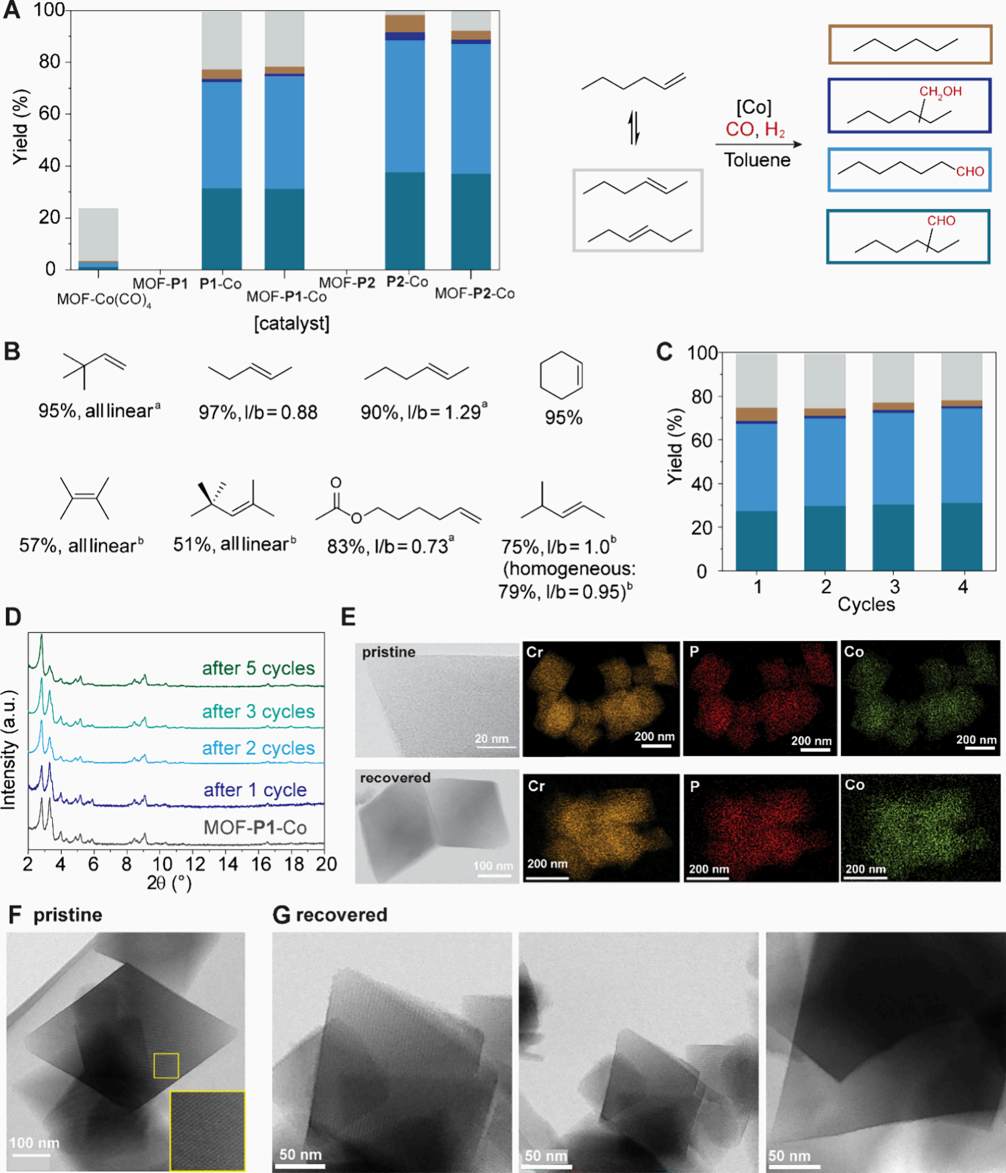
Performance of MOF-**P1**-Co in Olefin Hydroformylation Catalysis. **A** Comparison of the performance of MOF-**P1**-Co with that of the homogeneous catalyst as well as the MOF-immobilized Co­(CO)_4_ anion. **B** Substrate scope of MOF-**P1**-Co hydroformylation catalysis; [Co] loading increased to (a) 1.0 mol %, (b) 3.0 mol %, for detailed reaction condition see SI. **C** Recycling of MOF-**P1**-Co catalyst in 1-hexene hydroformylation. **D** Comparison of PXRD patterns of pristine and recovered catalyst after different numbers of reaction cycles. **E** Comparison of TEM images and EDX mapping of pristine MOF-**P1**-Co (top row) and catalyst recovered after three cycles (bottom row). Comparison of STEM images of pristine (**F**) and recovered (**G**) MOF-**P2**-Co.

Interestingly, a slight decrease in the undesired hydrogenation product was observed upon heterogenization, while the amount of aldehyde product obtained remained unperturbed. Comparison of homogeneous and heterogeneous catalytic performance was carried out for the fourth reuse of MOF-**P1-**Co, and the first use of MOF-**P2-**Co to demonstrate that the MOF-based catalyst reproduces the homogeneous product distribution at different points of the lifetime of the recyclable catalyst. Negligible catalytic activity was observed with MOF-Co­(CO)_4_ (Figure 5A), however, which suggests that the cobalt speciation of the precatalyst MOF-**P1**-Co (which contains an equimolar ratio of Co­(CO)_3_(**P1**)_2_^–^ and [Co­(CO)]_4_^–^) is altered under the reaction conditions to permit both cobalt centers in the precatalyst to actively contribute to catalysis.

In our exploration of the substrate scope, we focused on longer-chain terminal olefins and substrates containing an internal double bond, since efficient cobalt-catalyzed olefin isomerization permits an isomerization-hydroformylation sequence (Figure 5B and Figure S51). While light olefins are industrially hydroformylated via rhodium catalysis, cobalt catalysis is the preferred solution for longer-chain olefins, internal olefins, or when both the linear and branched isomer of the product are desired.^59,89^ With a catalyst loading of MOF-**P1**-Co that corresponds to a Co loading of 0.5 mol %, 3,3-dimethylbut-1-ene was selectively transformed to the terminal aldehyde product with a conversion of 95%. Cyclic alkenes such as cyclohexene could be efficiently converted and ester groups were readily tolerated. Internal alkenes such as 2-pentene and 2-hexene both proceeded smoothly to deliver the corresponding products in good to excellent yield (97% and 90%). While 75% conversion could be achieved for 4-methylpent-2-ene, a l/b ratio of 1 was observed for aldehyde products. To confirm that the low degree of regioselectivity was not due to MOF-heterogenization, we carried out hydroformylation under homogeneous conditions with an identical cobalt loading and Co:**P1** ratio, for which 79% conversion and a l/b ratio of 0.95 was obtained (Figure 5B). Challenging branched substrates such as 2,4,4-trimethyl-2-pentene and 2,3-dimethyl-2-butene still furnished conversions of 51% and 57%, respectively, to the corresponding hydroformylation products, and the terminal aldehyde was exclusively obtained.^90^ In the presence of MOF-**P1**-Co corresponding to 3 mol % cobalt, even a tetra-substituted olefin underwent Co-catalyzed hydroformylation to furnish exclusively the terminal aldehyde product via isomerization followed by hydroformylation. To probe whether the spacious 1.6 nm pore windows present in the MIL-101 support would permit even large substrates to access the confined catalyst, we selected cholesterol, which has a length of 1.9 nm and a width of 0.8 nm. While the local steric hindrance surrounding the double bond rendered both homogeneous and heterogeneous hydroformylation sluggish, essentially identical ratios of remaining olefin and aldehyde were obtained for both the homogeneous and the MOF-based catalyst (Figure S53 and S54). A control experiment with a TBDMS-protected cholesterol derivative furthermore demonstrated that the unprotected hydroxyl group did not lower the efficiency of the hydroformylation reaction. To rule out the possibility that the MOF-based catalyst functions through release of a catalytically active species into solution, we tested the hydroformylation of an olefin-functionalized Merrifield resin.^61^ While significant conversion was observed when hydroformylation was carried out under homogeneous conditions, MOF-**P1**-Co catalysis furnished no conversion for the resin-bound substrate within 55 h (Figure S55). The disparate outcomes indicate that the active catalyst in MOF-**P1**-Co is localized within the MOF pores and it does not leave the MOF pores over the course of >2 d under the reaction conditions. Despite the effective confinement the MOF provides for the catalyst, however, large substrates such as cholesterol are still able to access the active sites without apparent mass transfer limitations. To further probe the efficiency of mass transfer through the pore system of the MOF we prepared MOF­(MW)-**P1**-Co from a batch of Cr-MIL-101 synthesized in a microwave, which had a substantially reduced average crystallite size (Figure S15). A comparison of the product distribution after 1 h (Table S10) demonstrated that the rate of 1-hexene hydroformylation does not depend on the crystallite size of the porous host, so that active sites throughout the MOF appear to be efficiently sampled by the substrate.

We next turned our attention to the quantification of metal leaching; cobalt leaching is notoriously problematic for heterogeneous hydroformylation due to the strong interaction between cobalt and CO, which can lead to cobalt leaching from bulk metal, supported nanoparticles, single-atom catalysts, or supported complexes.^91–100^ In our initial attempts at MOF-**P1**-Co recycling (Figure S58), we observed a largely undiminished catalyst activity over five cycles, but also an increase in the Cr:Co ratio of the catalyst from 5.58 to 5.96 upon recycling, which would imperil the long-term stability of the catalyst. We found that a high-temperature washing step of the as-synthesized precatalyst in THF at 170 °C led to a perceptible change in the cobalt speciation: While a minor contribution of a linear Co^0^ dimer was initially present (Figure 4F, 6A), the washed sample only showed CO stretches corresponding to node-bound Co­(CO)_3_ **P**_2_ and Co­(CO)_4_ anions (Figure 6A). When THF-washed MOF-**P1**-Co samples were subjected to recycling (Figure 5C), a maximum of 0.7 ppm cobalt leaching could be observed while phosphine leaching was barely detectable (<0.05 ppm). Notably, the degree of cobalt leaching into the organic solvent observed with Co/pore-tethered **P1** (<0.7 ppm) was substantially lower than that previously reported for biphasic Co/**P1** hydroformylation catalysis (9 – 60 ppm).^81^ To further verify that no catalytically active species are leached into solution, we carried out hot filtration experiments at different time points during the reaction (Figure S59). A comparison of the PXRD patterns of pristine and recycled MOF-**P1**-Co samples (Figure 5D) confirmed that the crystallinity of the MOF support was retained upon repeated use in catalysis, while HR-(S)­TEM images and EDX mapping showed that the dispersion of both P and Co remained unchanged after three catalytic cycles (Figure 5E, 5F, 5G, Figure S60). Interestingly, recycling of MOF-**P1**-Co gave rise to a slight increase in the amount of aldehyde products and a decrease in the fraction of the hydrogenated side product, which is consistent with the need to convert the precatalyst present in the as-synthesized material into the catalytically active species. IR analyses of catalyst recovered after exposure to the hydroformylation reaction demonstrates that the catalyst resting state is gradually formed during cycle 1 and then remains intact upon recycling (Figure 6A), so that a well-defined cobalt speciation is present throughout the entire reaction time for recycled MOF-**P1**-Co­(R), but not for either the homogeneous or the pristine heterogeneous catalyst.

**Figure 6:**
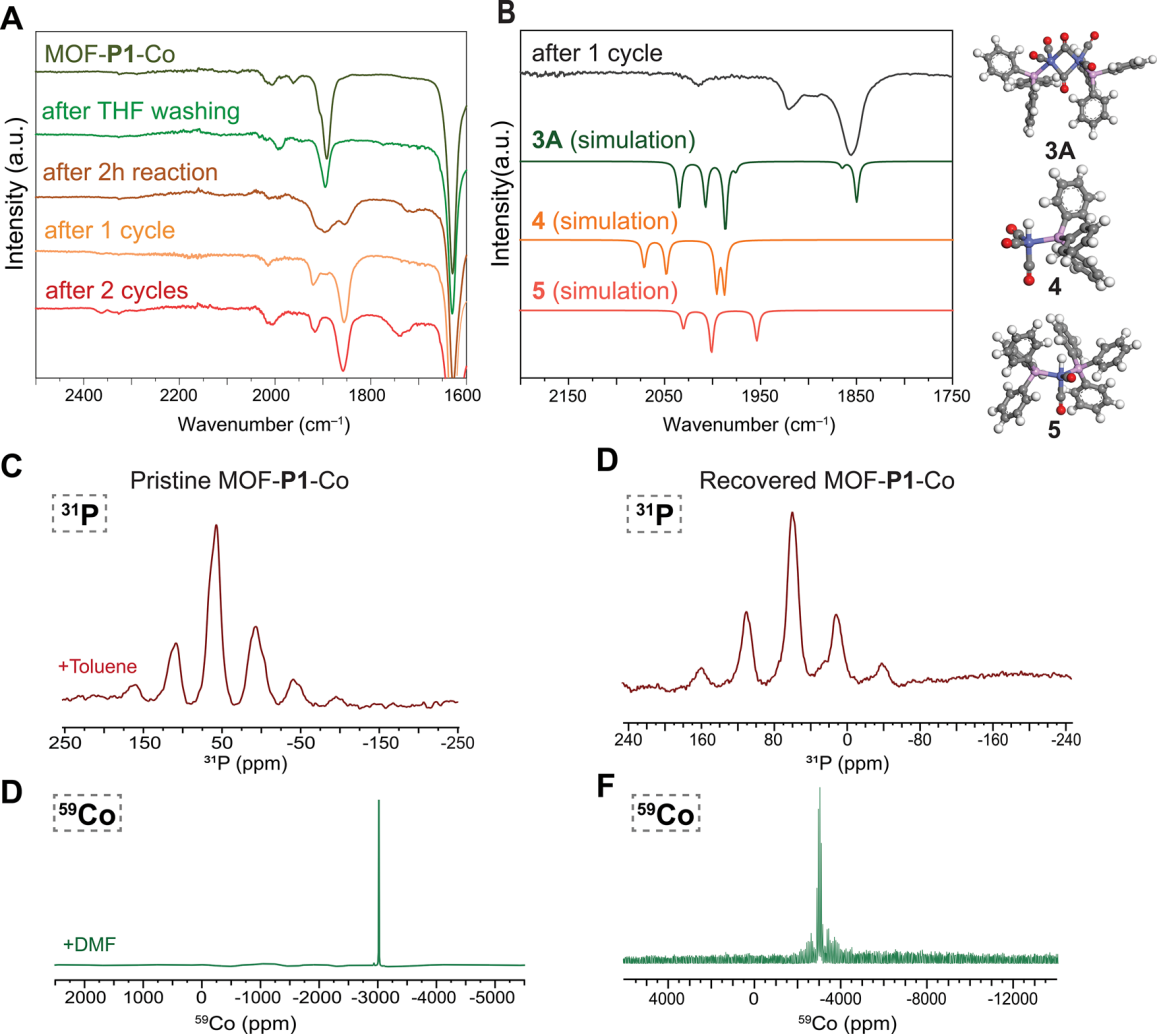
Adaptable Heterogeneous Ligand Platform Permits the Precatalyst to Evolve in Catalytically Active Co Complex. **A** Comparison of ATR-IR spectrum of pristine catalysts MOF-**P1**-Co and recovered catalysts after THF washing and different cycles. **B** Comparison of ATR-IR spectrum of recovered catalysts after 1 cycle and simulated spectra of complexes **3A**, **4**, and **5**. **C** ^31^P SS-NMR spectrum of pristine MOF-**P1**-Co with toluene-solvated pores (MASr = 10 kHz). **D** Static ^59^Co SS-NMR spectrum of pristine MOF-**P1**-Co with DMF-solvated pores (16384 scans). **E** ^31^P SS-NMR spectrum of recovered MOF-**P1**-Co­(R) (MASr = 10 kHz). **F** Static ^59^Co SS-NMR spectrum of recovered MOF-**P1**-Co­(R) (49540 scans).

Prior studies showed that Co­(CO)_3_ **P**_2_ Co­(CO)_4_ salts, which are structurally analogous to the MOF-**P1**-Co precatalyst, are not catalytically active in hydroformylation.^101^ In solution, an increase in the reaction temperature leads, depending on the nature of the phosphine ligand, to the formation of linear (**2**) or bridged (**3**) dimeric Co^0^ complexes **P**-Co­(CO)_3_-Co­(CO)_3_-**P**, which can then undergo cooperative oxidative addition to form the catalytically active Co^I^ cobalt-hydride **4**.^80^ To probe whether a similar reorganization of cobalt oxidation states and phosphine coordination takes place within the MOF pore, we compared IR spectra of samples recovered from the reaction conditions after 2 or 6 h with that of the precatalyst (Figure 6A). Samples recovered from the reaction conditions showed a gradual disappearance of the precatalyst bands and the appearance of a carbonyl stretch at 1850 cm^–1^, which indicates the presence of a dimeric complex with bridging carbonyl ligands. Based on theoretical models (Figure S68) and comparison with CO stretches of reported structures, we provide bridged dimer **3A** as a tentative structure model for the catalyst resting state. While linear dimer **2** is thermodynamically favored over bridged dimer **3A** in homogeneous solution, the energy difference between the two structures is minor.^86^ We hypothesize that the formation of the bridged dimer may be favored within the MOF since it can accommodate a wide range of distances between the sulfonate groups on the phosphine ligands and could thus be stabilized by pairs of MOF nodes with a wide range of different distances. The need for an encounter between two pore-confined cobalt centers to convert the Co^–I^/Co^I^ precatalyst into the dimeric Co^0^ resting state accounts for our earlier observation that catalysts containing a higher phosphine loading furnished improved performance. Each pore in the optimized MOF-**P1**-Co catalyst contains an average of 6.4 Co centers, which facilitates encounters between the mobile cobalt centers while maximizing the residual pore volume available for substrate occupation and transport.

To obtain direct evidence for the mobility of pore-confined cobalt phosphines we collected ^59^Co (Figure 6D and Figure S42) and ^31^P (Figure S43) NMR data in the presence of DMF. A complete disappearance of the spinning side bands was observed for the [Co­(CO)_4_]^−^ derived ^59^Co signal, which supports substantial mobility of the tetrahedral anion in the presence of the polar solvent. The emergence of a sharp peak was likewise observed for the ^31^P signal derived from [Co­(CO)_3_(**P1**)_2_]^−^ when the pores were filled with DMF, but the coordinating solvent also led to the partial displacement of phosphine ligands from the Co^I^ center (Figure S43). When DMF was replaced with toluene, no change in the appearance of the ^31^P spectrum (Figure 6C) could be observed compared to a spectrum acquired in the absence of solvent (Figure 4E). So while **P1** ligands were found to achieve substantial mobility even in the presence of decane (Figure 3A), a polar solvent was required to achieve notable mobility for the [Co­(CO)_3_(**P1**)_2_]^−^ complex at room temperature. We attribute this discrepancy to the fact that **P1** is tethered to a single MOF SBU, whereas [Co­(CO)_3_(**P1**)_2_]^−^ contains two **P1** ligands that are charge-tethered to two distinct SBUs, so that the mobility of the complex is reduced compared to the ligand.

To gain additional information about the structure of the catalyst resting state that could thus be assembled within the MOF pore, we collected ^31^P SS-NMR of the recovered catalyst (MOF-**P1**-Co­(R)). We observed the presence of a single dominant phosphorus species with a chemical shift centered at 59 ppm (Figure 6E). The chemical shift confirms that the phosphines remain ligated to cobalt, while the appearance of a single ^31^P resonance is consistent with structure **3A** proposed for the catalyst resting state in which the two phosphine ligands are magnetically equivalent. Further support was provided by the P 2p X-ray photoelectron spectrum of MOF-**P1**-Co­(R), which likewise showed only a single phosphorus environment (Figure S67). We anticipated that observation of a ^59^Co signal for structure **3A** would not be possible, because multiple literature reports confirmed that the symmetry of the ^59^Co environment of bridged cobalt dimers ligated by two phosphine ligands is too low to permit NMR detection.^82^ Indeed only a weak ^59^Co SS-NMR signal could be obtained for MOF-**P1**-Co­(R) with a chemical shift of – 3040 ppm (Figure 6F) after an extended measurement time (49540 scans), which we attribute to residual [Co­(CO)_4_]^−^ in the sample. While the remaining concentration of [Co­(CO)_4_]^−^ present in the recovered catalyst was too low to permit detection by ATR, the appearance of a band at 1920 cm^–1^ (which does not originate from the dimeric carbonyl bridged Co^0^ catalyst resting state) indicates the presence of an additional species in the recovered catalyst. We predicted the IR stretches for a range of potential reaction intermediates and side products, among which only bis-phosphine ligated Co^I^ hydride **5** provided a reasonable match for the experimentally observed IR data (Figure 6B, Figure S68).^102,103^ Since a definite identification of cobalt–carbonyl hydrides is extremely challenging, however, further in-depth studies are required to determine whether the low mobility in dry MOF pores can sufficiently slow down cooperative bimolecular reductive elimination of H_2_ to stabilize pore-confined cobalt-hydrides.^104^

However, clear evidence for the key role of solvent induced mobility on catalytic activity could be obtained through hydroformylation experiments of liquid and gaseous substrates in the presence and absence of added solvent (Figure 7A). No conversion was observed when propene was subjected to syngas and MOF-**P1**-Co in the absence of added solvent. However, when 0.15 mL toluene was added to the reaction mixture to moisten the catalyst, C_4_ oxygenated products were formed with low efficiency (TON = 26). When the amount of added toluene was increased to 10 mL, the cobalt-catalyst contained within fully solvated MOF pores gave rise to a TON of 934. Likewise, in the case of 1-hexene, no conversion was observed in the absence of added solvent, while efficient cobalt-catalyzed hydroformylation took place in the presence of toluene or 1,4-dioxane (Figure 7A).^105,106^ To rule out that the solvent plays a mechanistic role in the reaction other than engendering mobility of the heterogenized catalyst, we carried out 1-hexene hydroformylation under neat conditions: When 5 mL of 1-hexene substrate was used to ensure that the MOF pores were filled with liquid, a comparable TON value was obtained than for 1-hexene hydroformylation in the presence of solvent. Notably, the l/b ratio for the neat reaction was 0.4 compared with 1.4–1.6 in the presence of solvent, which may be due to olefin enrichment within the MOF pores under the neat reaction conditions.^54^ Homogeneous-like activity and selectivity can thus be obtained with the MOF-heterogenized catalyst, as long as sufficient liquid is present within the supercages to permit them to serve as nanoreactors for mobile catalyst species.

**Figure 7:**
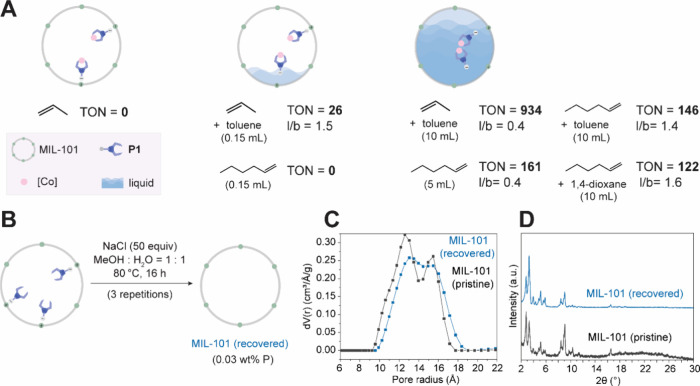
**A** The need for liquid in the MOF pore for efficient turnover substantiates that mobility is required for catalytic activity. **B** Recovery of MOF support. **C** Comparison of pore size distributions of pristine and recovered MIL-101 derived from Ar sorption data. **D** Comparison of PXRD patterns of pristine and recovered MIL-101.

Of course, even a recyclable catalyst will eventually lose catalytic activity, which makes it highly desirable that the functional support material used for heterogenization can be efficiently recovered from spent catalyst for refitting with fresh catalyst and reuse.^14,107^ We thus tested whether charge-tethered phosphines can be fully removed from MOF-**P1** and the MIL-101 support can be recovered without structural damage. Three repetitions of a treatment of MOF-**P1** with an excess of NaCl in a methanol/water mixture at 80 °C for 16 h led to complete removal of phosphine ligand (Figure 7B) from the MOF pores. Analysis of the recovered support material showed full retention of the crystallinity (Figure 7D), as well as the pore volume (1.65 cm^3^·g^–1^ for recovered MIL-101 compared to 1.66 cm^3^·g^–1^ for the pristine material), which renders the support ready for reloading with fresh phosphine. Notably, earlier attempts to remove **P1** from the MOF pore in the presence of excess LiCl or NaCl in methanol at 80 °C did not lead to phosphine removal (Figure S69A), so that MOF-**P1** can function as leaching-proof heterogeneous ligands even under forcing reaction conditions.

## Conclusion

We show that sulfonated phosphine ligands can be heterogenized within the spacious supercages of the cheap and chemically stable MOF MIL-101. Solid-state ^31^P NMR studies provide direct evidence that the heterogenized phosphine ligands enjoy substantial mobility in the presence of solvent, and display a high degree of structural homogeneity. To mimic the performance of homogeneous cobalt hydroformylation catalysts, a mobile heterogeneous ligand set is required in order to stabilize the precatalyst as well as diverse reaction intermediates which have different nuclearity, oxidation states, and phosphine coordination environments. The MOF-based catalyst MOF-**P1**-Co faithfully reproduces the performance of the homogeneous catalyst even after repeated recycling, and thus constitutes a mechanistically homogeneous and functionally heterogeneous catalyst. In-depth characterization of the structure of the MOF-confined cobalt precatalyst could be achieved via a combination of SC-XRD, ^59^Co and ^31^P NMR as well as IR spectroscopy, and the gradual evolution of the precatalyst to the catalyst resting state was monitored via IR spectroscopy. The high stability of the MOF matrix permits the recovery of the MOF support from MOF-**P1**, so that the MOF can be further recycled after the heterogenized phosphine becomes deactivated. The facile heterogenization of a variety of phosphine ligands is expected to permit a facile extension of mechanistically homogeneous and functionally heterogeneous catalysis to transformations involving structurally diverse phosphines.

## Supplementary Material





## Notes

Experimental procedures, additional characterization data, single-crystal X-ray structure analysis, coordinates for theoretical structures (PDF)
Raw data of gas sorption isotherms in the form of adsorption information format (AIF) files (ZIP)
