# Supplementary material for: Click Heterogenization of Phosphines Furnishes Recyclable Hydroformylation Catalysts that Reproduce Homogeneous Performance
Source: J Am Chem Soc. 2025 Sep 11;147(48):44087–100. doi: 10.1021/jacs.5c10989 (PMC12679637; doi:10.1021/jacs.5c10989)
Supplement: Supplementary file 1 [file ja5c10989_si_001.pdf]

Supporting Information

Click Heterogenization of Phosphines Furnishes Recyclable Hydroformylation Catalysts that Reproduce Homogeneous Performance

*Junjun Chen[1,2,3], Christophe Farès[1], Aamir Abbas[1], and Constanze N. Neumann[1]**

[1]Max-Planck-Institut für Kohlenforschung, Kaiser-Wilhelm-Platz 1, 45470, Mülheim an der Ruhr, Germany.

[2]CAS Key Laboratory of Low-Carbon Conversion Science and Engineering, Shanghai Advanced Research Institute, Chinese Academy of Sciences, Shanghai 201210, PR China.

[3]University of the Chinese Academy of Sciences, Beijing 100049, PR China.

*neumann@kofo.mpg.de

Contents

Supporting Information 1
1 General Information 4
1.1 Chemicals 4
1.2 Experimental Techniques 5
2 Synthetic Procedures 8
2.1 Synthesis of Cr-MIL-101-Cl 8
2.2 Synthesis of MOF(MW) 8
2.3 Synthesis of MOF-**P** 9
2.3.1 Synthesis of MOF- x**P1** 9
2.3.2 Calculation of the Average Pore Occupancy of MOF-x**P1** 10
2.3.3 Synthesis of MOF-x**P** 10
2.4 Synthesis of MOF-**P**-Co 11
2.5 Synthesis of MOF-$Co(CO)_4$ 13
2.5.1 $Na[Co(CO)_4]$ 13
2.5.2 MOF-$Co(CO)_4$ 14
2.6 Synthesis of $[Co(CO)_3(PPh_3)_2][Co(CO)_4]$ 14
2.7 Synthesis of **S1** 15
3 Temporal Evolution of Rh and Co Prices 16
4 Mechanism of Homogeneous Hydroformylation 17
5 Characterization Data 18
5.1 X-ray Crystallography 18
5.1.1 X-ray structure of $[Co(CO)_3(PPh_3)_2]^+[Co(CO)_4]^-$ 18
5.2 Powder X-ray Diffraction 22
5.3 SEM Images and Energy Disperse X-ray Spectroscopy 27
5.4 Gas Sorption 30
5.5 Thermal Gravimetric Analysis 37
5.6 NMR Spectroscopy 40
5.1 Infrared Spectroscopy 47
6 Catalysis 49
6.1 Standard Experimental Procedure for Heterogeneous Hydroformylation 49
6.2 Optimization of Reaction Conditions 49
6.2.1 Reaction Temperature 49

6.2.2 Total Pressure and CO / H_2 Ratio 50
6.2.3 Reaction Time 50
6.3 Optimization of Catalyst Composition 51
6.3.1 Effect of **P1** Loading 51
6.3.2 Effect of P / Co Ratio 51
6.4 Control Reaction with Cr-MIL-101 52
6.5 Kinetic experiments 53
6.6 Evaluation of Potential Mass Transfer Limitations with MOF(MW)-**P1**-Co 54
6.7 Substrate Scope 54
6.8 Hydroformylation of Bulky Substrates 55
6.9 Hydroformylation of Resin-Bound Substrate 59
6.9.1 Procedure for Hydroformylation of Olefin-Functionalized Merrifield Resin 60
7 Recycling Experiments 62
7.1 Experimental Procedure 62
7.2 Recycling of MOF-**P1**-Co that was not washed with THF 62
7.3 Recycling of THF-Washed Catalyst 63
7.4 Hot filtration 64
8 Comparison of Pristine and Recovered Catalyst 65
8.1 Electron Microscopy 65
8.2 Nuclear Magnetic Resonance 68
8.3 X-ray Photoelectron Spectroscopy 70
9 Structure of the Catalyst Resting State 72
10 Recovery of MIL-101 from MOF-**P1** 73
10.1 Unsuccessful Phosphine Removal with Excess Salt 73
10.2 Successful Phosphine Removal with Excess Salt and Water 73
11 Computational Details 75
11.1 List of DFT-Optimized Atomic Coordinates 76
12 References 95

1 General Information

1.1 Chemicals

Unless otherwise indicated, chemicals and solvents were obtained from commercial suppliers (Table S1), and used as received. Deuterated solvents were obtained from Eurisotop. Dry and degassed solvents (pentane, tetrahydrofuran, and toluene) were dried by distillation from an appropriate drying agent in the technical laboratories of the Max-Planck-Institut für Kohlenforschung and stored in Schlenk flasks under argon. Dry argon was purchased from Air Liquide with >99.5% purity.

Table S1. Reagents obtained from commercial suppliers.

**Chemical Name**	**Supplier**
Chromium(III) nitrate nonahydrate<br>Dodecane	Sigma-Aldrich Chemie GmbH
Benzene-1,4-dicarboxylic acid<br>Sodium Diphenylphosphinobenzene-3-sulfonate (Na-**P1**)<br>2-Pentene (cis- and trans- mixture)<br>4-Methyl-2-pentene (cis- and trans- mixture)	TCI Deutschland GmbH
Triphenylphosphine (TPP)	Alfa Aesar
4-(1,3,5-Triaza-7-phosphaadamantan-1-ium-1-yl)butane-1-sulfonate (**P6**)<br>Sodium 3,3',3''-phosphinetriyltribenzenesulfonate (Na-**P2**)<br>Sodium 2'-(dicyclohexylphosphino)-2,6-dimethoxy-[1,1'-biphenyl]-3-sulfonate (Na-**P3**)<br>Sodium 4,5-bis(diphenylphosphino)-9,9-dimethyl-9H-xanthene-2,7-disulfonate (Na-**P4**)	BLD Pharmatech GmbH
Hydrogen fluoride (48%-51%)<br>cis-2-Hexene<br>Dicobaltoctacarbonyl<br>Methanol (extra dry)<br>1-Hexene<br>2,4,4-Trimethyl-2-pentene<br>3-(Di-tert-butylphosphonium)propane sulfonate (H-**P5**)	Fisher Scientific GmbH
Sodium hydroxide	VWR International GmbH

1.2 Experimental Techniques

Powder X-ray diffraction (PXRD): PXRD measurements were conducted on a STOE STADI P diffractometer operating in Debye Scherrer geometry with a Cu Kα radiation source and a primary germanium monochromator. Data were acquired with a HyPix-3000 multi-dimensional detector in 1D mode. Samples were placed in the capillaries and data were acquired continuously in the 2θ range of 2 – 50° with steps of 0.01° and a scan speed of 0.5° min^{-1}. The measured samples were qualitatively evaluated by comparison with entries from the ICDD PDF-2 database.

Single crystal X-ray diffraction (SC-XRD): SC-XRD data were recorded on Bruker AXS Enraf-Nonius KappaCCD diffractometer with a FR591 rotating Mo-anode X-ray source and a Bruker-AXS Kappa Mach3 with APEX-II detector and IµS microfocus Mo-anode X-ray source.

Electron Microscopy (EM): High-resolution transmission electron microscopy (HRTEM) images were collected with a Thermo Scientific Talos F200X (S) TEM microscope with an acceleration voltage of 200 kV with a Super-X EDS system. The sample was deposited on a copper grid covered with a Lacey carbon membrane layer. High-angle annular dark-field scanning-transmission electron micrographs (HAADF-STEM) and energy dispersive X-ray spectroscopy (EDX) elemental mapping were obtained on a Cs probe-corrected Hitachi HD-2700 microscope equipped with a cold field emission gun and two EDAX Octane T ultra W EDX detectors at an acceleration voltage of 200 kV. High magnification Scanning Electron Microscopy SEM-images were recorded on a S-5500 In-lens FE-SEM from Hitachi Europe with 30 kV of acceleration voltage. Energy-dispersive X-ray Spectroscopy (EDX) measurements were conducted using a NORAN System 7 X-ray Microanalysis System with UltraDry EDS Detector 30 mm^2 (both from Thermo Fisher Scientific). The samples were prepared by sprinkling on carbon Lacey-Film on copper grids.

Gas Chromatography (GC): Gas chromatography (GC) measurements were performed using an Agilent 7890A gas chromatograph equipped with a capillary column (DB-624, i.d. 0.25 mm, df. 0.25 µm, 30 m). The column oven temperature started from 35 °C (10 min hold) and was then increased to 250 °C (10 min hold) at a heating rate of 20 °C · min^{-1}. The split was 80:1, injector temperature of 350 °C and detector temperature of 220 °C.

Mass Spectrometry (MS): GC-EI or GC-CI were performed in ISQ Series Single Quadrupol GC-MS with Trace GC Ultra and AI 1310 Autosampler (Thermo Scientific, San Jose, CA, USA) or Q Exactive GC Orbitrap with Trace 1310 GC and TriPluse Autosampler (Thermo Scientific, San Jose, CA, USA).

Infrared Spectroscopy: Attenuated total reflectance-Fourier transform infrared spectroscopy (diamond crystal) was measured using an Agilent Cary 630 FTIR. Spectra were recorded between 4000 and 650 cm^{-1} range (4 cm^{-1} resolution, 128 scans per spectrum) at room temperature inside an Ar-filled glovebox.

Nuclear Magnetic Resonance (NMR) Spectroscopy: NMR data were recorded using a Bruker AVIII HD 300 MHz, Bruker AVIII 500, Bruker AVNeo 600 MHz NMR spectrometer at 298/300 K unless indicated otherwise. Chemical shifts (δ) are given in ppm relative to TMS, coupling constants (J) in Hz. The solvent signals were used as internal references and the chemical shifts converted to the TMS scale ($CDCl_3$: δ_H = 7.26 ppm, δ_C = 77.16 ppm; CD_2Cl_2: δ_H = 5.32 ppm, δ_C = 53.84 ppm; C_6D_6: δ_H = 7.16 ppm; δ_C = 128.06 ppm DMSO-d_6: δ_H = 2.50 ppm, δ_C = 39.52 ppm; THF-d_8: δ_H = 3.58, 1.72 ppm, δ_C =67.21, 25.31 ppm).[108] Multiplicity (s = singlet, d = doublet, dd = doublet of doublets, t = triplet, td = triplet of doublets, q = quartet, quint = quintet, sext = sextet, sept = septet, m = multiplet, br = broad singlet) was used to report data.

All solid-state NMR (SS-NMR) spectra were recorded on a Bruker Avance III HD 500WB spectrometer using double-bearing MAS probes (DVT BL4) at resonance frequencies of 500.192 MHz, 202.481 MHz and 118.68 MHz for ^{1}H, ^{31}P, and ^{59}Co. The chemical shifts were referenced indirectly relative to an external TMS sample. All NMR spectra were recorded at a temperature of 298 K (sensor temperature). All spectra were processed in Topspin 3.6. Samples were packed into a 4 mm ZrO_2 rotor from Bruker and a KelF or Vesp rotor cap (for the acquisition of ^{59}Co SS-NMR data of the recovered catalyst, a 7 mm ZrO_2 rotor was used that contained 95 mg sample). For air sensitive samples, the powder was transferred to the rotor within a glove box.

The ^{59}Co Wideband Uniform Smooth Truncation-Carr-Purcell Meiboom-Gill (WURST CPMG) spectra were recorded on a non-spinning sample with a sweep width of 33900 ppm and a carrier frequency of –5800 ppm. About 25000 scans were accumulated with a recycling delay of 3 s (27 h). The FID was recorded over a duration of 5 ms and included 40 CPMG echo loops consisting of a 80 µs WURST pulse centered between 10 µs delays for ring-down spacing (receiver off) and a 53 µs acquisition delay (receiver on) resulting in the spikelet separation of 8000 Hz. The WURST pulse (pulse length = 5 µs, sweepwidth = 4000000 Hz, power index = 80) was generated within the shape tool module of Topspin 3.6. ^{1}H-decoupling was achieved with the tppm15 pulse train at 96 kHz.

The ^{31}P SS-NMR spectra were obtained under MAS = 8 – 12 kHz using the bruker "hpdec" pulse sequence consisting of an excitation pulse of 1 µs corresponding to about 36 degrees flip angle and a recycling delay of 4 seconds. ^{1}H-decoupling was achieved with the tppm 15 pulse train at 96 kHz.

Argon Sorption: Argon gas sorption measurements were performed on a Micro 200C from 3P Instruments at 87 K. Sample activation was carried out by drying on a high-vacuum line followed by attachment of the sample to the instrument's activation port under high vacuum at 150 °C for 12 h. For BET surface area calculations, all quality criteria outlined by IUPAC are met (positive C value, $n \cdot (1 - p \cdot p_0^{-1})$ increases continuously with the relative pressure, the relative pressure corresponding to the monolayer capacity is within the BET range). The correlation coefficients for the fitted range were >0.9999. Pore size distributions were obtained from the argon sorption data with the Quantachrome ASiQwin software by applying the model "Ar at 87 K on carbons (cylindr. pores, QSDFT equilibrium model)".

Inductively Coupled Plasma Optical Emission spectroscopy (ICP-OES): ICP-OES were performed using a Spectrogreen FMX 46 Typ 76004566 DSOI (Dual Side On Interface) and a UVPlus Optic ORCA (Optimized Rowland Circle Alignment). Solid sample was treated with HCl and HNO_3 and then cooked for 10 min in an Anton Paar Multiwave 5000. After acid and microwave treatment, the liquid was nebulized into an aerosol using a nebulizer and transported into the plasma torch with an argon gas flow. The plasma provided sufficient energy to excite atoms and ions in the sample. The measurement was carried out 3 times. The obtained data from all three measurements was averaged and then processed and reported in terms of the measured concentration for each detected element.

Thermogravimetric analysis (TGA): TGA was done on a Netzsch STA 449F3 instrument under constant flow of air (40 $mL \cdot min^{-1}$) by heating the sample (around 8 mg) from 40 °C to 800 °C at a heating rate of 10 $°C \cdot min^{-1}$. Data analysis was carried out with the Netzsch Proteus – Thermal Analysis software (version 6.1.0).

X-ray Photoelectron Spectroscopy (XPS): XPS analysis was carried out using a VG ESCALAB 220i-XL with an X-ray source employing monochromatic Al Kα anode (1486.6 eV), operated at 200 W and 15 kV. The

analysis chamber maintained a base pressure of 5×10^{-10} mbar. The C 1s peak for contaminant carbon was used as a reference at 284.5 eV for correction of the binding energy for surface charging.

Glovebox: Argon was used as the glovebox atmosphere with O_2 < 1 ppm and H_2O < 0.1 ppm.

2 Synthetic Procedures

2.1 Synthesis of Cr-MIL-101-Cl

Cr-MIL-101 was synthesized according to a reported method.[109] Specifically, $Cr(NO_3)_3 \cdot 9H_2O$ (6.406 g, 16.00 mmol), terephthalic acid (2.697 g, 16.23 mmol) and 72 mL distilled water were added to a PEEK-lined 200 mL stainless steel autoclave reactor. Additionally, HF (48%, 0.6 mL, 16 mmol) was added. The autoclave was heated in a heating mantle at 220 °C for 8 h (the internal temperature reached 220 °C within 60 minutes). After completion of the reaction, the reaction mixture was removed and subjected to centrifugation (20 min at 4500 rpm) to separate the solid product, which was then washed twice with distilled water. The resulting solid was transferred into a flask to which 180 mL water and then 1.5 mL 38% HCl were added before it was stirred at 50 °C for 4 hours. Treatment with HCl ensured that fluoride and hydroxide anions bound to chromium nodes of MIL-101 were replaced with chloride anions (Figure S1). After anion exchange, the solid was washed with DMF (2 x 40 mL), EtOH (2 x 40 mL) and acetone (2 x 40 mL). In each case, the MOF was allowed to soak in the solvent for at least 1 h. After the supernatants were discarded for the last time, the resulting solid was placed it an oven at 60 °C for 8 hours and subsequently activated at 150 °C under dynamic vacuum for 24 hours to yield Cr-MIL-101-Cl.

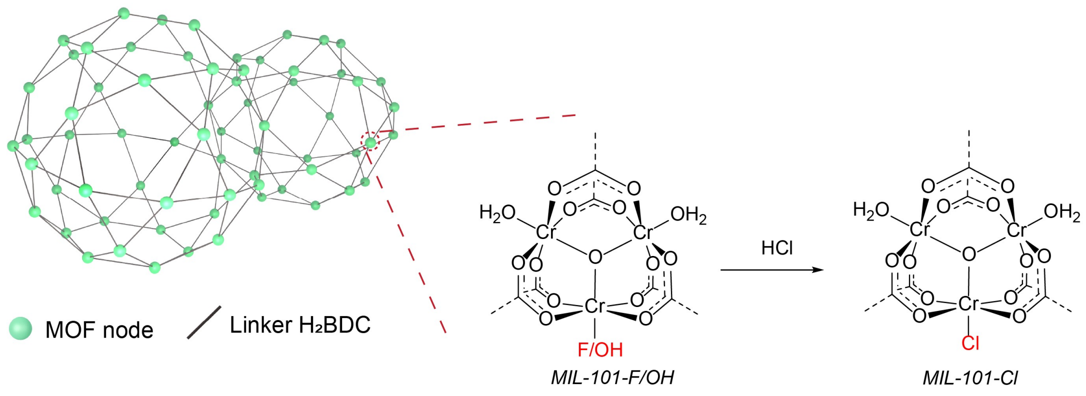

Figure S1. Exchange of node-bound fluoride or hydroxyl group for node-bound chloride using HCl.[110-111]

2.2 Synthesis of MOF(MW)

Cr-MIL-101 with a substantially reduced crystallite size was synthesized according to a reported microwave-based procedure.[112] The as-synthesized MOF was treated with HCl to ensure that fluoride and hydroxide anions bound to chromium nodes of MIL-101 were replaced by Cl (Figure S1). After anion exchange, the solid was washed with DMF (2 x 40 mL), EtOH (2 x 40 mL) and acetone (2 x 40 mL). In each case, the MOF was allowed to soak in the solvent for at least 1 h. After the supernatants were discarded for the last time, the resulting solid was placed it an oven at 60 °C for 8 hours and subsequently activated at 150 °C under dynamic vacuum for 24 hours to yield MOF(MW).

2.3 Synthesis of MOF-P

2.3.1 Synthesis of MOF- xP1

In an argon-filled glovebox, Cr-MIL-101-Cl (100.0 mg), 80 mL methanol and the requisite amount (Table S2) of Na-**P1** were added to a 250 mL flask containing a stir bar (PTFE, cylindrical, 20 × 7 mm), then the resulting suspension was stirred (400 rpm) overnight at room temperature. The resulting green suspension was transferred to 45 mL centrifuge tubes, sealed, and subjected to centrifugation (4500 rpm for 15 min). The supernatant was discarded and 40 mL THF was added to the tubes, which was then shaken to produce a suspension and subjected to centrifugation followed by removal of the supernatant. Washing with THF was then repeated two more times. After the supernatant had been discarded for the last time, the solid remaining in the centrifugation tube was left to dry under Ar flow overnight and subsequently stored inside a glovebox.

The compounds obtained were named MIL-101- x**P1** were x indicates the average number of **P1** ligands that are present per super-cage. The phosphorus content of the samples was determined by ICP-OES analysis, and the experimentally determined Cr : P ratios were converted into an average pore content of **P1** according to method described in Section 2.2.2.

Table S2. Average number of **P1** ligands present per MIL-101 super-cage in MOF-P1 samples prepared with the amounts of Na-**P1** in the column labeled n [mmol].

sample	n [mmol]	Mass [g]	P wt%	Cr wt%	Cr/P	P1 / cage
MOF-2.0**P1**	0.010	0.0036	0.46	13.70	17.82	2.0
MOF-2.6**P1**	0.0300	0.0109	0.58	12.65	13.09	2.6
MOF-4.3**P1**	0.0500	0.0182	0.96	13.42	8.37	4.3
MOF-6.2**P1**	0.0700	0.0255	0.91	8.85	5.82	6.2

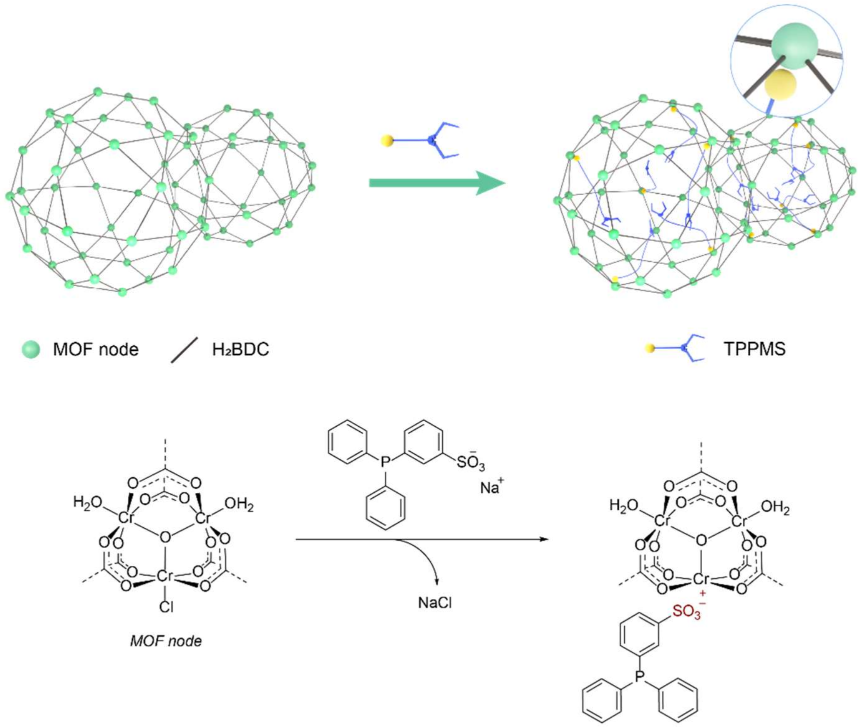

Figure S2. Synthesis of MOF- x**P1**.

2.3.2 Calculation of the Average Pore Occupancy of MOF-xP1

To calculate the average number of phosphines per MOF cage from the experimentally determined ratio of Cr : P in MOF-x**P** the number of Cr centers per super-cage in MIL-101 had to be determined. Each sphere in the simplified structural diagram (Fig. S2) represents a MOF secondary building unit (SBU) which is composed of three chromium atoms of which one carries an X-type ligand (Fig. S1). The large cage is made up of 42 SBUs, and the small cage of 30 SBUs, but each SBU is shared between three cages. Therefore, the large cage can at most take up 14 charge-tethered phosphines and the small cage can take up at most 10 phosphines. Since we cannot experimentally differentiate between phosphine located in the small or in the large cage we calculate the average pore occupancy of both pores by multiplying the P : Cr ratio determined by ICP-OES by the average number of SBUs present in a cage as follows:

$$x = \frac{\mathrm{P}}{Cr} * \frac{42 + 30}{2}$$

2.3.3 Synthesis of MOF-xP

The syntheses of MOF-x**P** with phosphine ligands other than **P1** were carried out according to the same procedure as that described in detail MOF-x**P1** in section 2.2.1. In Figure S3 the molar amount of the respective sodium salt of the phosphine ligand that was used is shown along with the average number of phosphine ligands detected in the resulting product.

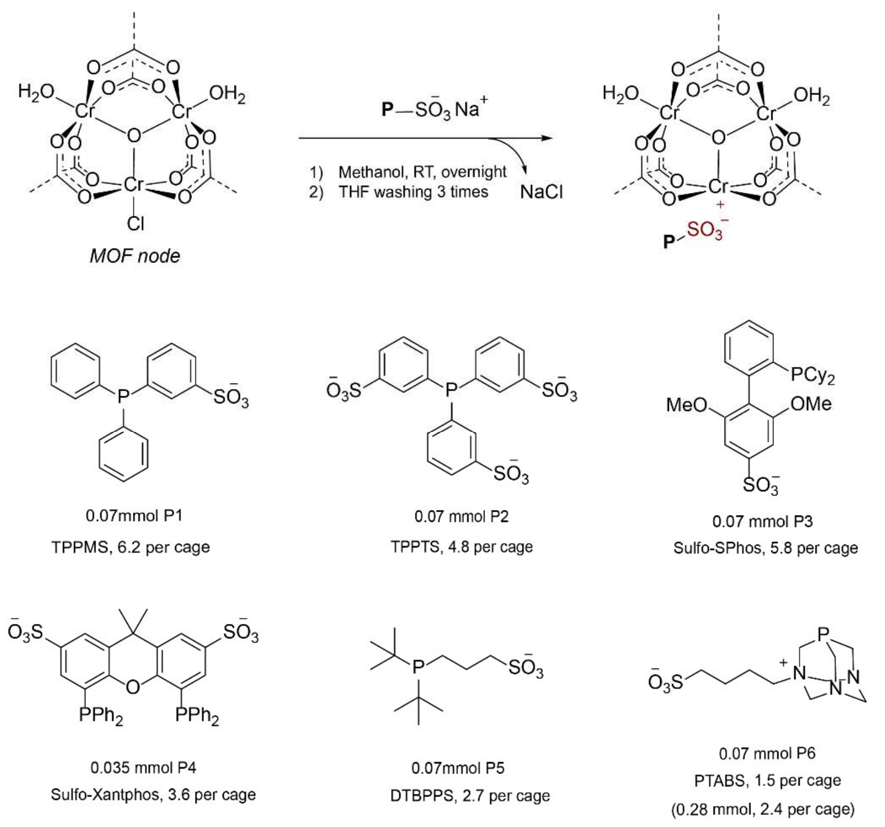

Figure S3. Synthesis of MOF-x**P**.

2.4 Synthesis of MOF-P-Co

The amount of $Co_2(CO)_8$ added to prepare MOF-**P**-Co was calculated based on the experimentally determined phosphine content of the MOF-**P** sample and the desired Co : P ratio in the MOF-**P**-Co sample (Table S3). See Table S4 for experimentally determined Co : P ratios observed when different amounts of $Co_2(CO)_8$ were used in the synthesis of MOF-**P1**-Co from MOF-6.2**P1**.

To a flask containing MOF-x**P** (100 mg) and $Co_2(CO)_8$, THF (80 mL) was added and the reaction mixture was stirred overnight (450 rpm). The resulting suspension was transferred to 45 mL centrifuge tubes, sealed, and then subjected to centrifugation (4500 rpm for 15 min). The recovered solid was washed with THF (3 x 40 mL), at which point the supernatant had become transparent. The solid thus obtained rivals the catalytic activity of the related homogeneous catalyst, but leaching of cobalt is observed upon recycling (see section 7). To ensure minimal Co leaching, as-synthesized MOF-**P**-Co is washed with THF at 170 °C for 6 h prior to use in catalysis. Since cobalt carbonyl complexes are temperature sensitive, the high-temperature washing step is carried out in the presence of syngas (20 bar CO and 20 bar H_2) which prevents the decomposition of the cobalt catalyst during the washing step. The THF washing step serves to remove traces of loosely bound cobalt (3.12 ppm Co were detected in the recovered THF) and alters the speciation of cobalt within the MOF pore (see discussion in main text). The solid recovered from the THF wash was transferred to a vial, dried using Ar flow overnight and stored in a glovebox freezer until further use.

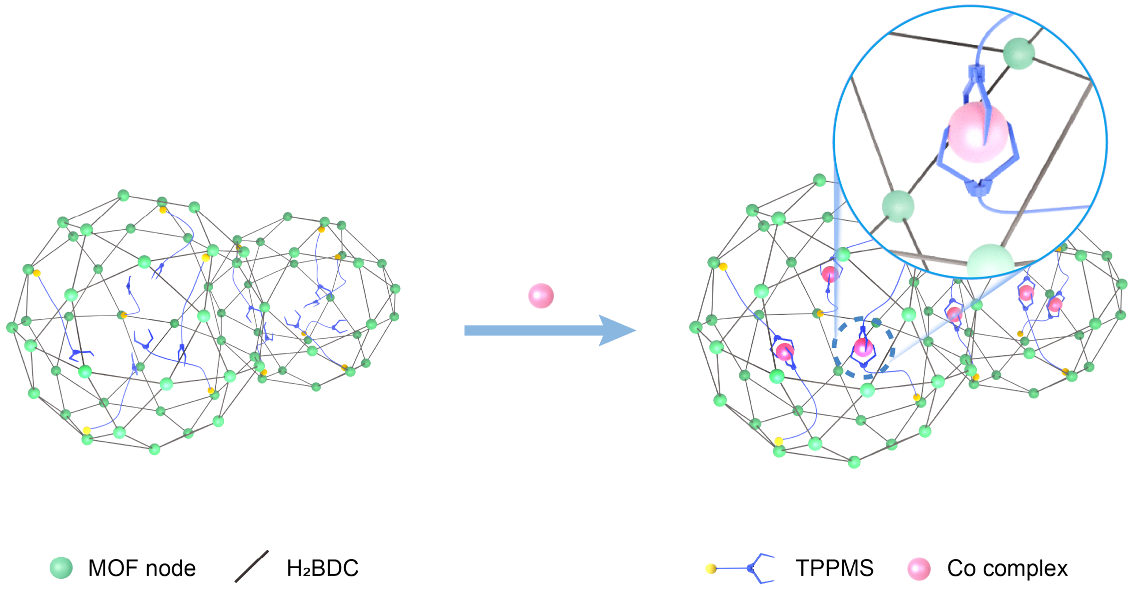

Figure S4. Synthesis of MOF-**P**-Co.

Table S3. Average number of **P1** ligands and cobalt centers present in each MIL-101 cage in MOF-**P1**-Co for samples prepared with different amounts of Na-**P1** and $Co_2(CO)_8$ precursors.

sample	P1 [mmol]	$Co_2(CO)_8$ [mmol]	Cr/Co	Cr/P	Co/P	P1 / cage	Co / cage
MOF-2.0**P1**-2.6Co	0.01	0.01	13.66	17.82	1.30	2.0	2.6
MOF-2.7**P1**-7.7Co	0.03	0.03	4.66	13.09	2.81	2.7	7.7
MOF-4.3**P1**-10.3Co	0.05	0.05	3.50	8.37	2.39	4.3	10.3
MOF-6.2**P1**-13.1Co	0.07	0.07	2.75	5.82	2.11	6.2	13.1
MOF-6.3**P1**-15.9Co	0.10	0.10	2.27	5.69	2.51	6.3	15.9

Table S4. Average number of **P1** ligands and cobalt centers present in each MIL-101 cage in MOF-**P1**-Co for samples prepared with different amounts $Co_2(CO)_8$ precursor but a constant amount of the Na-**P1** precursor of 0.07 mmol.

sample	P1 [mmol]	$Co_2(CO)_8$ [mmol]	Cr/Co	Cr/P	P/Co	P1 / cage	Co / cage
MOF-5.7**P1**-1.0Co	0.07	0.005	34.63	6.30	5.6	5.7	1.0
MOF-5.8**P1**-2.1Co	0.07	0.01	17.53	6.16	2.9	5.8	2.1
MOF-6.0**P1**-6.5Co	0.07	0.03	5.58	6.02	0.9	6.0	6.5
MOF-6.2**P1**-10.3Co	0.07	0.05	3.48	5.85	0.6	6.2	10.3
MOF-6.2**P1**-13.1Co	0.07	0.07	2.75	5.82	0.4	6.2	13.1

2.5 Synthesis of MOF-$Co(CO)_4$

2.5.1 $Na[Co(CO)_4]$

$Na[Co(CO)_4]$ was synthesized according to a reported method.[113] Specifically, a 250 mL Schlenk flask containing a magnetic stir bar was charged with $Co_2(CO)_8$ (5.00g, 14.6 mmol, 1.00 equiv) under an inert atmosphere. Freshly ground NaOH (6.00 g, 150 mmol, 10.3 equiv) dissolved in tetrahydrofuran (50 mL) was transferred via a cannula to the Schlenk flask, and the reaction mixture was left to stir in an ice bath for 6 h while wrapped in aluminum foil. The product mixture was then passed through a Celite pad and washed several times with tetrahydrofuran. The collected filtrate was concentrated and dried overnight under Ar atmosphere to obtain the final product in a yield of around 60%.

2.5.2 MOF-$Co(CO)_4$

In an argon-filled glovebox, a round-bottom flask was charged with MIL-101-Cl (80.0 mg, 0.109 mmol, 1.00 equiv.) and $Na[Co(CO)_4]$ (92.0 mg, 0.474 mmol, 4.35 equiv.) and methanol (80 mL) was added. The reaction mixture was left to stir (400 rpm) overnight at room temperature. The resulting suspension was transferred to 45 mL centrifuge tubes, sealed, and then subjected to centrifugation (4500 rpm for 15 min). The resulting solid was washed with THF (3 x 40 mL), at which point the supernatant became transparent. All the steps were carried out under inert atmosphere. The solid was transferred to a vial, dried overnight via Ar, and then stored inside a glovebox freezer.

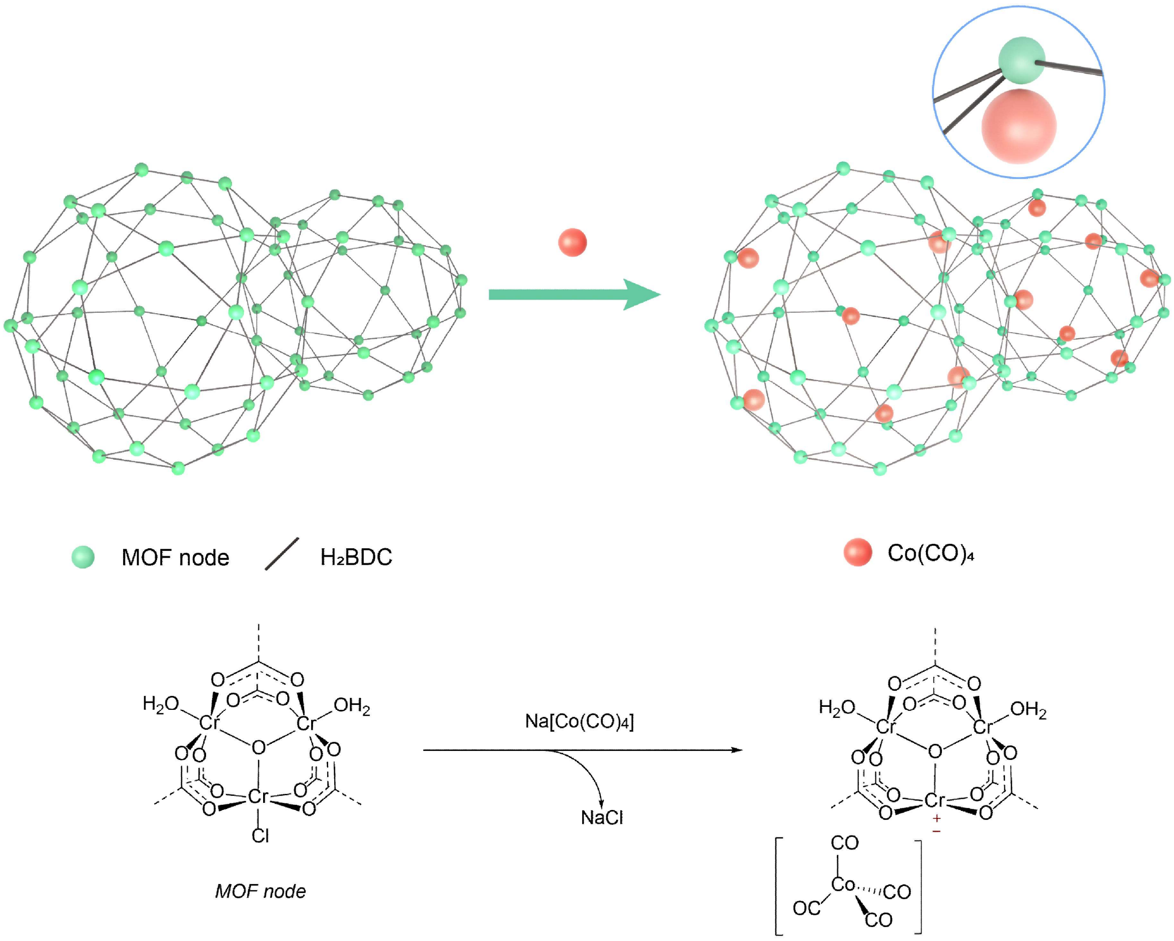

Figure S5. Synthesis of MOF-$Co(CO)_4$.

2.6 Synthesis of $[Co(CO)_3(PPh_3)_2][Co(CO)_4]$

The synthesis was carried out based on a reported method.[114] In the argon-filled glovebox, a 200 mL Schlenk flask was charged with 1,4-dioxane (70 mL) and heated to 30 °C. $Co_2(CO)_8$ (800 mg, 2.34 mmol, 1.00 equiv) was added into the flask, and after the solid had dissolved, a solution of triphenylphosphine (5.00 g, 19.1 mmol, 8.16 equiv) in 1,4-dioxane (10 mL) was slowly added to the flask under stirring. Stirring was continued for 20 min after the addition before the Schlenk flask was taken out the glovebox and allowed to cool down in an ice bath. The temperature of reaction mixture should be maintained at around

15 °C to permit crystallization of the target product. An appreciable amount of solid was visible within the flask after 1 hour, and the Schlenk flask was consequently transferred back into glovebox. The suspension was filtered and the recovered solid was washed with pentane (50 mL). The brown solid that was left behind was then dissolved in acetone (100 mL). The acetone solution was collected in a 250 mL flask and left to stand overnight, during which time a precipitate formed. The supernatant was then decanted into a vial which was removed from the glovebox and to which water that had been degassed with an argon stream was slowly added to encourage crystallization. An orange-red solid was obtained in around 70% yield (only an approximate value could be determined due to the residual water content of the sample).

Single crystals suitable for X-ray analysis were obtained when 1 mL of the acetone solution described above was slowly added on top of 5 mL Ar-saturated water and the resulting layered liquid was left without moving under room temperature for 7 days.

2.7 Synthesis of S1

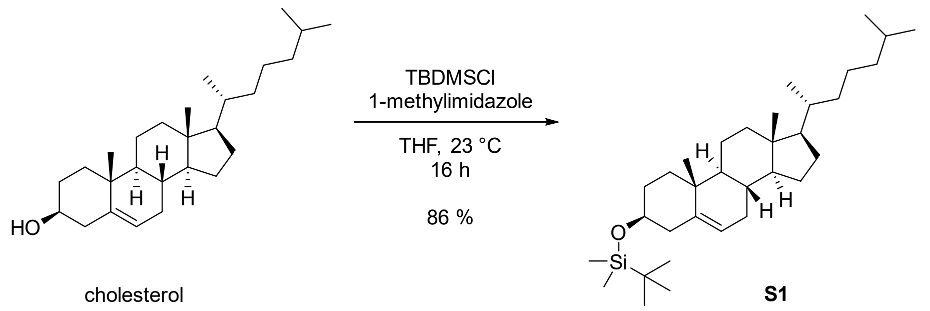

The synthesis of **S1** was carried out according to a reported method,[115] and the spectral data collected for **S1** match previously reported data.

^{1}H NMR (600 MHz, $CDCl_3$) δ 5.31 (dt, J = 5.4, 2.1 Hz, 1H, 6), 3.48 (tt, J = 11.1, 4.7 Hz, 1H, 3), 2.30 – 2.23 (m, 1H, 4'), 2.16 (ddd, J = 13.4, 5.0, 2.3 Hz, 1H, 4''), 1.71 (ddd, J = 13.1, 4.1, 2.5 Hz, 1H, 2'), 1.01 – 0.98 (m, 3H, 19), 0.91 (d, J = 6.5 Hz, 3H, 21), 0.89 (s, 9H, 30), 0.87 (d, J = 6.6 Hz, 3H, 26), 0.86 (d, J = 6.6 Hz, 3H, 27), 0.67 (s, 3H, 18), 0.06 (s, 6H, 28).

^{13}C NMR (151 MHz, $CDCl_3$) δ 141.74 (5), 121.32 (6), 72.81 (3), 56.97 (14), 56.30 (17), 50.38 (9), 42.98 (4), 42.49 (13), 39.97 (12), 39.68 (24), 36.75 (10), 35.93 (20), 32.25 (2), 32.07 (8), 28.39 (16), 26.10 (30), 24.45 (15), 23.97 (23), 22.97 (26), 22.71 (27), 21.22 (11), 19.59 (19), 18.87 (21), 18.43 (29), 12.01 (18), -4.43 (28).

^{29}Si NMR (119 MHz, $CDCl_3$) δ 16.77.

3 Temporal Evolution of Rh and Co Prices

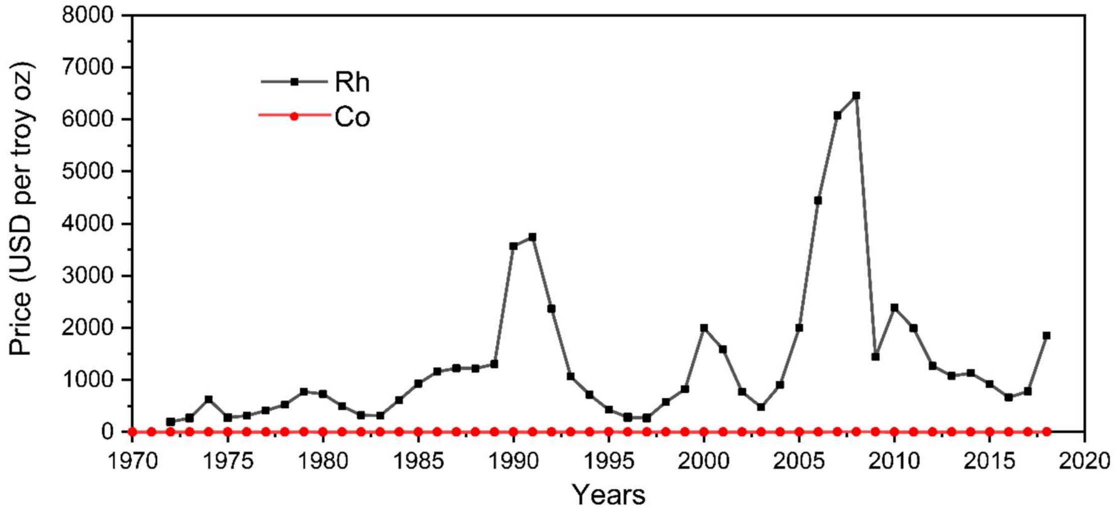

Figure S6. Comparison of the price of a troy ounce of Rhodium (Rh) and Cobalt (Co) metal between 1973 and 2018.[116] Notably, rhodium is not only substantially more expensive than cobalt, it also demonstrates huge price fluctuations, which creates challenges for industrial use since the amount of capital required for catalyst purchase can vary drastically.

4 Mechanism of Homogeneous Hydroformylation

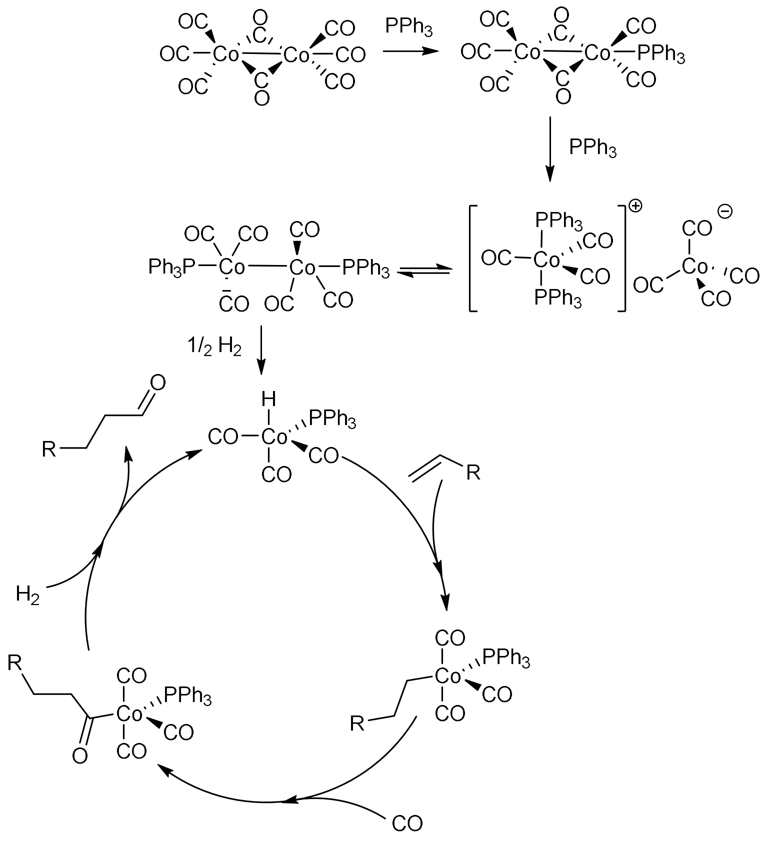

Figure S7. Accepted mechanism for homogeneous hydroformylation catalyzed by $Co_2(CO)_8$ and triphenylphosphine.[117]

5 Characterization Data

5.1 X-ray Crystallography

5.1.1 X-ray structure of $[Co(CO)_3(PPh_3)_2]^+[Co(CO)_4]^-$

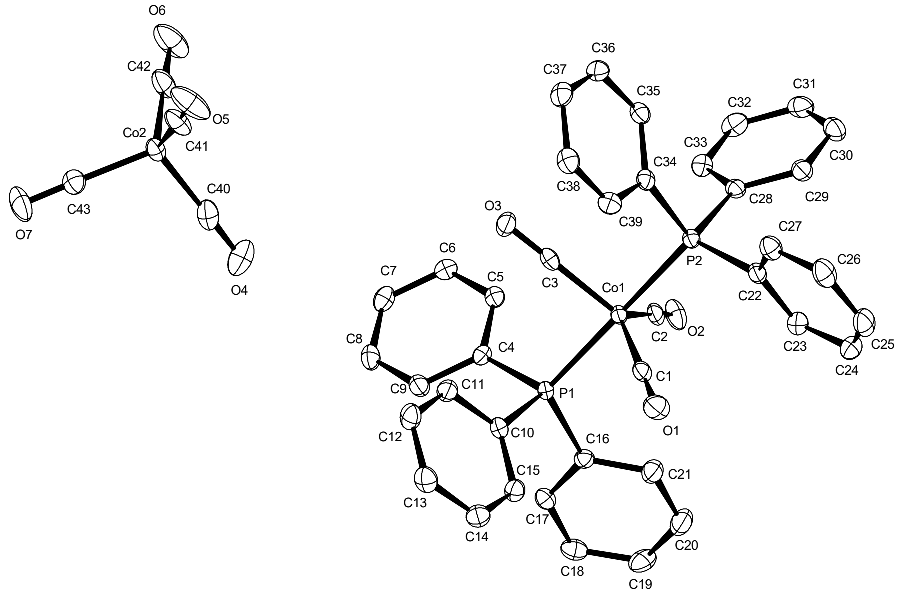

Figure S8. X-ray structure of $[Co(CO)_3(PPh_3)_2][Co(CO)_4]$. The cif file is available under the CCDC number CCDC-2466986.

Table S5. Crystal data and structure refinement.

Identification code	16078	
Empirical formula	$C_{43} H_{30} Co_2 O_7 P_2$	
Color	colourless	
Formula weight	838.47 g · mol^{-1}	
Temperature	100(2) K	
Wavelength	0.71073 Å	
Crystal system	MONOCLINIC	
Space group	**$P2_1/c$, (no. 14)**	
Unit cell dimensions	a = 10.1274(3) Å	α= 90°.
	b = 19.0267(6) Å	β= 103.476(2)°.
	c = 20.2766(6) Å	γ= 90°.
Volume	3799.5(2) Å^3	

Z	4	
Density (calculated)	1.466 Mg · m^{-3}	
Absorption coefficient	1.009 mm^{-1}	
F(000)	1712 e	
Crystal size	0.125 x 0.113 x 0.030 mm^3	
θ range for data collection	2.066 to 26.412°.	
Index ranges	-12 ≤ h ≤ 12, -23≤ k ≤ 23, -25 ≤ l ≤ 25	
Reflections collected	165787	
Independent reflections	7786 [R_{int} = 0.0859]	
Reflections with I>2σ(I)	6541	
Completeness to θ = 25.242°	100.0 %	
Absorption correction	Gaussian	
Max. and min. transmission	0.97 and 0.91	
Refinement method	Full-matrix least-squares on F^2	
Data / restraints / parameters	7786 / 0 / 487	
Goodness-of-fit on F^2	1.047	
Final R indices [I>2σ(I)]	R_1 = 0.0286	wR^2 = 0.0644
R indices (all data)	R_1 = 0.0395	wR^2 = 0.0700
Largest diff. peak and hole	0.4 and -0.3 e · $Å^{-3}$	

Table S6. Bond lengths [Å] and angles [°].

Co(1)-P(2)	2.2584(5)	Co(1)-P(1)	2.2425(5)
Co(1)-C(1)	1.797(2)	Co(1)-C(2)	1.784(2)
Co(1)-C(3)	1.805(2)	P(2)-C(22)	1.8165(19)
P(2)-C(28)	1.8182(19)	P(2)-C(34)	1.8134(19)
P(1)-C(4)	1.8211(19)	P(1)-C(10)	1.8108(19)
P(1)-C(16)	1.8231(19)	O(1)-C(1)	1.138(2)
O(2)-C(2)	1.142(2)	O(3)-C(3)	1.138(2)
C(4)-C(5)	1.392(3)	C(4)-C(9)	1.401(3)
C(5)-C(6)	1.385(3)	C(6)-C(7)	1.378(3)
C(7)-C(8)	1.389(3)	C(8)-C(9)	1.389(3)
C(10)-C(11)	1.397(3)	C(10)-C(15)	1.391(3)

C(11)-C(12)	1.382(3)	C(12)-C(13)	1.391(3)
C(13)-C(14)	1.381(3)	C(14)-C(15)	1.390(3)
C(16)-C(17)	1.395(3)	C(16)-C(21)	1.391(3)
C(17)-C(18)	1.385(3)	C(18)-C(19)	1.385(3)
C(19)-C(20)	1.383(3)	C(20)-C(21)	1.388(3)
C(22)-C(23)	1.399(3)	C(22)-C(27)	1.391(3)
C(23)-C(24)	1.385(3)	C(24)-C(25)	1.384(3)
C(25)-C(26)	1.381(3)	C(26)-C(27)	1.391(3)
C(28)-C(29)	1.394(3)	C(28)-C(33)	1.394(3)
C(29)-C(30)	1.392(3)	C(30)-C(31)	1.384(3)
C(31)-C(32)	1.383(3)	C(32)-C(33)	1.388(3)
C(34)-C(35)	1.388(3)	C(34)-C(39)	1.401(3)
C(35)-C(36)	1.395(3)	C(36)-C(37)	1.380(3)
C(37)-C(38)	1.390(3)	C(38)-C(39)	1.380(3)
Co(2)-C(40)	1.768(2)	Co(2)-C(41)	1.767(2)
Co(2)-C(42)	1.752(2)	Co(2)-C(43)	1.762(2)
O(4)-C(40)	1.151(3)	O(5)-C(41)	1.149(3)
O(6)-C(42)	1.157(3)	O(7)-C(43)	1.155(2)
P(1)-Co(1)-P(2)	178.33(2)	C(1)-Co(1)-P(2)	89.76(6)
C(1)-Co(1)-P(1)	89.37(6)	C(1)-Co(1)-C(3)	117.98(8)
C(2)-Co(1)-P(2)	93.56(6)	C(2)-Co(1)-P(1)	88.11(6)
C(2)-Co(1)-C(1)	124.45(9)	C(2)-Co(1)-C(3)	117.40(9)
C(3)-Co(1)-P(2)	90.75(6)	C(3)-Co(1)-P(1)	88.39(6)
C(22)-P(2)-Co(1)	114.59(6)	C(22)-P(2)-C(28)	103.93(8)
C(28)-P(2)-Co(1)	116.14(6)	C(34)-P(2)-Co(1)	108.97(6)
C(34)-P(2)-C(22)	106.75(9)	C(34)-P(2)-C(28)	105.73(9)
C(4)-P(1)-Co(1)	113.50(6)	C(4)-P(1)-C(16)	106.73(8)
C(10)-P(1)-Co(1)	111.43(6)	C(10)-P(1)-C(4)	104.02(8)
C(10)-P(1)-C(16)	106.78(9)	C(16)-P(1)-Co(1)	113.68(6)
O(1)-C(1)-Co(1)	179.57(18)	O(2)-C(2)-Co(1)	176.04(16)
O(3)-C(3)-Co(1)	178.95(17)	C(5)-C(4)-P(1)	120.17(14)
C(5)-C(4)-C(9)	119.09(17)	C(9)-C(4)-P(1)	120.74(14)
C(6)-C(5)-C(4)	120.47(17)	C(7)-C(6)-C(5)	120.38(18)
C(6)-C(7)-C(8)	119.87(18)	C(9)-C(8)-C(7)	120.27(18)

C(8)-C(9)-C(4)	119.91(18)	C(11)-C(10)-P(1)	117.83(14)
C(15)-C(10)-P(1)	122.39(14)	C(15)-C(10)-C(11)	119.74(17)
C(12)-C(11)-C(10)	120.10(18)	C(11)-C(12)-C(13)	119.88(19)
C(14)-C(13)-C(12)	120.28(18)	C(13)-C(14)-C(15)	120.14(19)
C(14)-C(15)-C(10)	119.85(18)	C(17)-C(16)-P(1)	119.71(14)
C(21)-C(16)-P(1)	121.13(15)	C(21)-C(16)-C(17)	119.16(18)
C(18)-C(17)-C(16)	120.27(18)	C(17)-C(18)-C(19)	120.18(19)
C(20)-C(19)-C(18)	119.92(19)	C(19)-C(20)-C(21)	120.2(2)
C(20)-C(21)-C(16)	120.26(19)	C(23)-C(22)-P(2)	118.64(15)
C(27)-C(22)-P(2)	121.65(15)	C(27)-C(22)-C(23)	119.60(18)
C(24)-C(23)-C(22)	120.07(19)	C(25)-C(24)-C(23)	119.9(2)
C(26)-C(25)-C(24)	120.4(2)	C(25)-C(26)-C(27)	120.2(2)
C(26)-C(27)-C(22)	119.8(2)	C(29)-C(28)-P(2)	120.68(15)
C(33)-C(28)-P(2)	119.81(14)	C(33)-C(28)-C(29)	119.45(17)
C(30)-C(29)-C(28)	120.02(19)	C(31)-C(30)-C(29)	120.12(19)
C(32)-C(31)-C(30)	120.06(19)	C(31)-C(32)-C(33)	120.3(2)
C(32)-C(33)-C(28)	120.09(18)	C(35)-C(34)-P(2)	120.89(14)
C(35)-C(34)-C(39)	119.44(17)	C(39)-C(34)-P(2)	119.29(14)
C(34)-C(35)-C(36)	119.81(18)	C(37)-C(36)-C(35)	120.44(19)
C(36)-C(37)-C(38)	119.88(18)	C(39)-C(38)-C(37)	120.11(18)
C(38)-C(39)-C(34)	120.29(18)	C(41)-Co(2)-C(40)	111.98(10)
C(42)-Co(2)-C(40)	107.14(11)	C(42)-Co(2)-C(41)	109.82(10)
C(42)-Co(2)-C(43)	112.75(10)	C(43)-Co(2)-C(40)	106.72(10)
C(43)-Co(2)-C(41)	108.44(9)	O(4)-C(40)-Co(2)	178.2(2)
O(5)-C(41)-Co(2)	177.6(2)	O(6)-C(42)-Co(2)	177.6(2)
O(7)-C(43)-Co(2)	178.68(19)		

5.2 Powder X-ray Diffraction

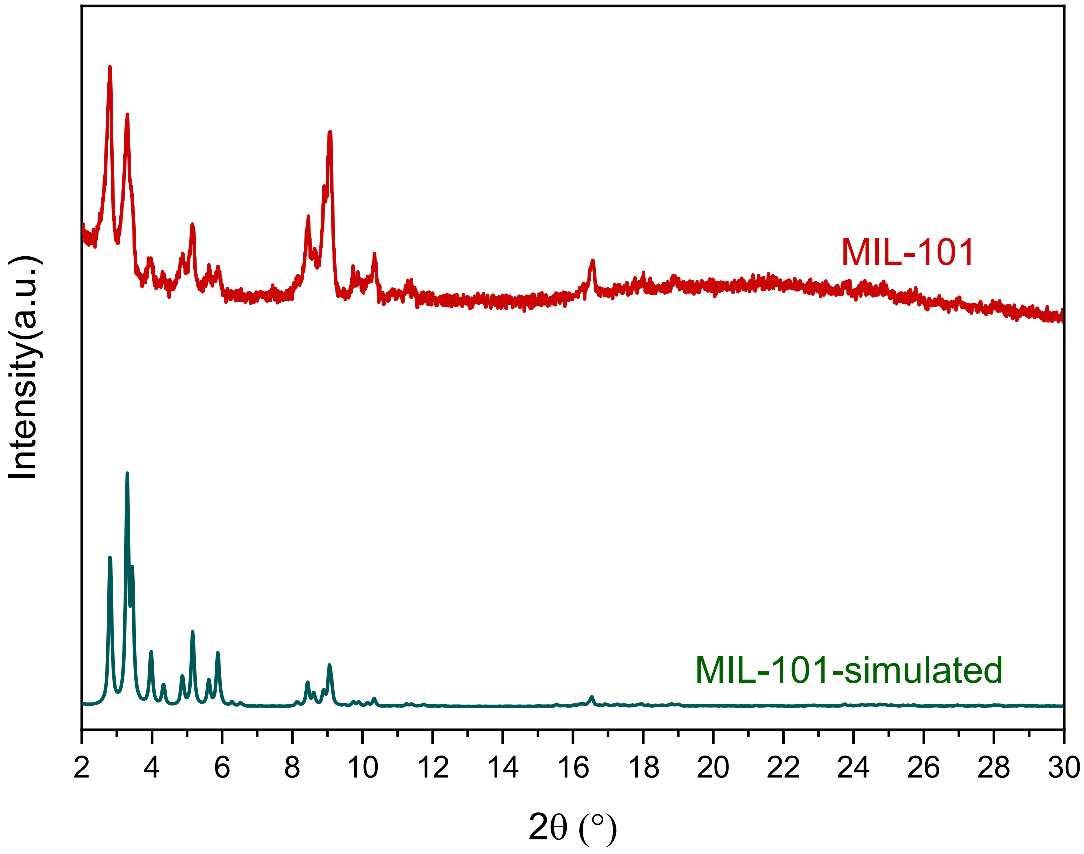

Figure S9. Comparison of the experimental PXRD pattern of MIL-101 with a simulation.

A comparison of the PXRD patterns of MOF-**P** containing the phosphine ligands **P1**-**P6** is provided in the main text.

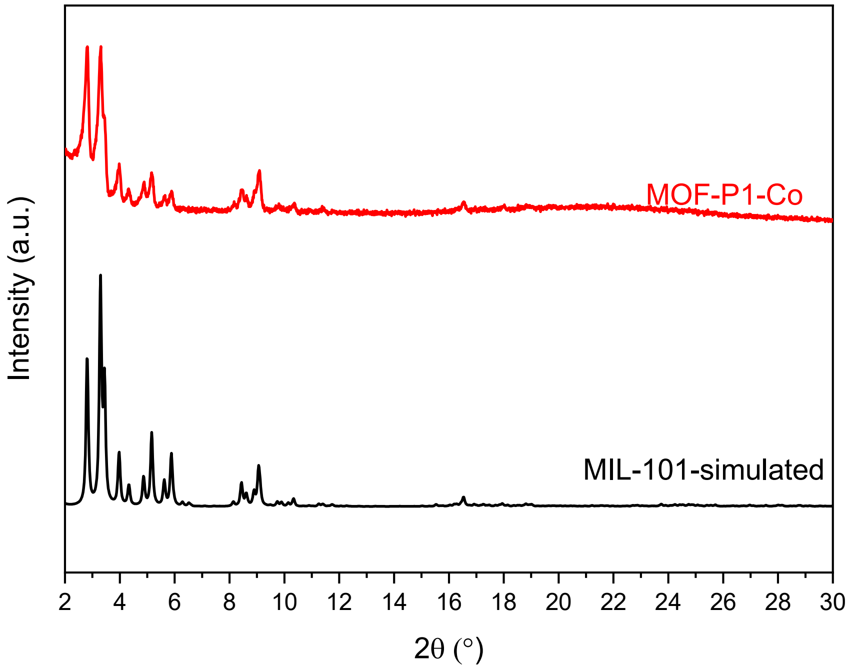

Figure S10. Comparison of the experimental PXRD pattern of MOF-**P1**-Co with a simulation for MIL-101.

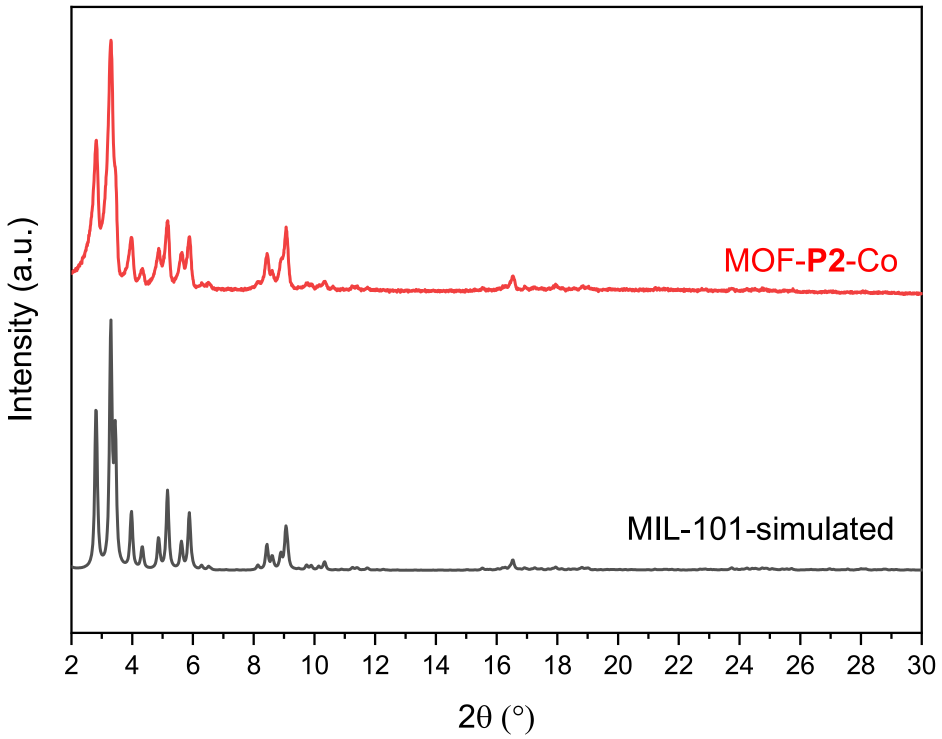

Figure S11. Comparison of the experimental PXRD pattern of MOF-**P2**-Co with a simulation for MIL-101.

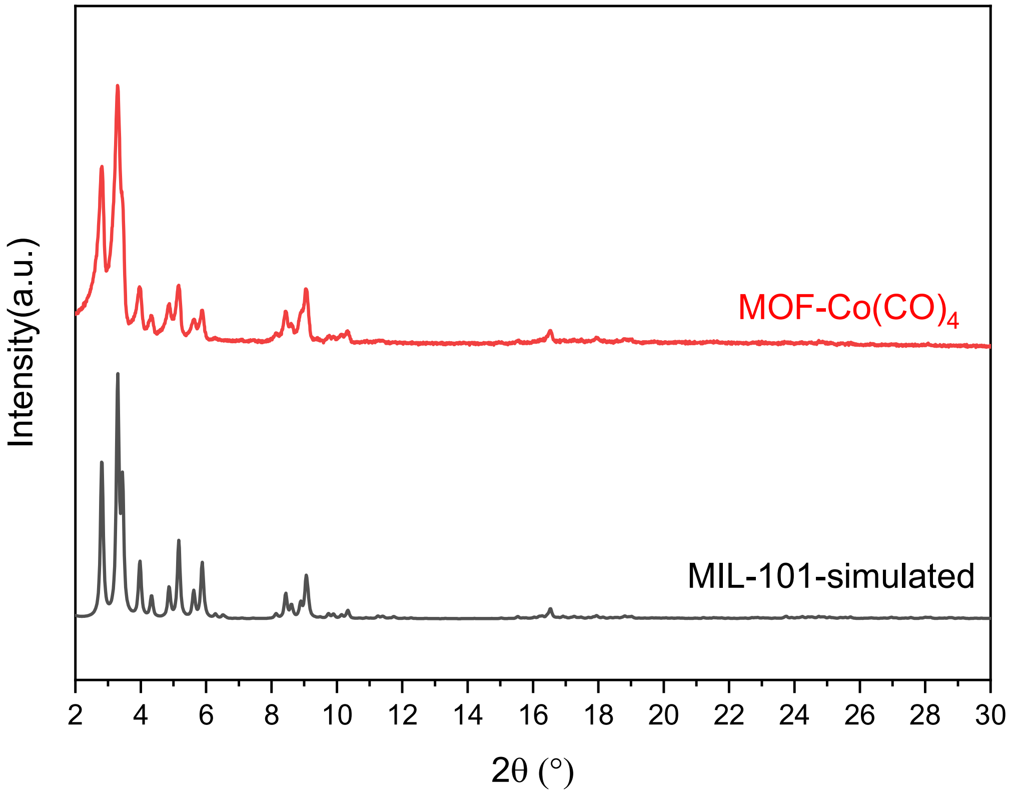

Figure S12. Comparison of the experimental PXRD pattern of MOF-$Co(CO)_4$ with a simulation for MIL-101.

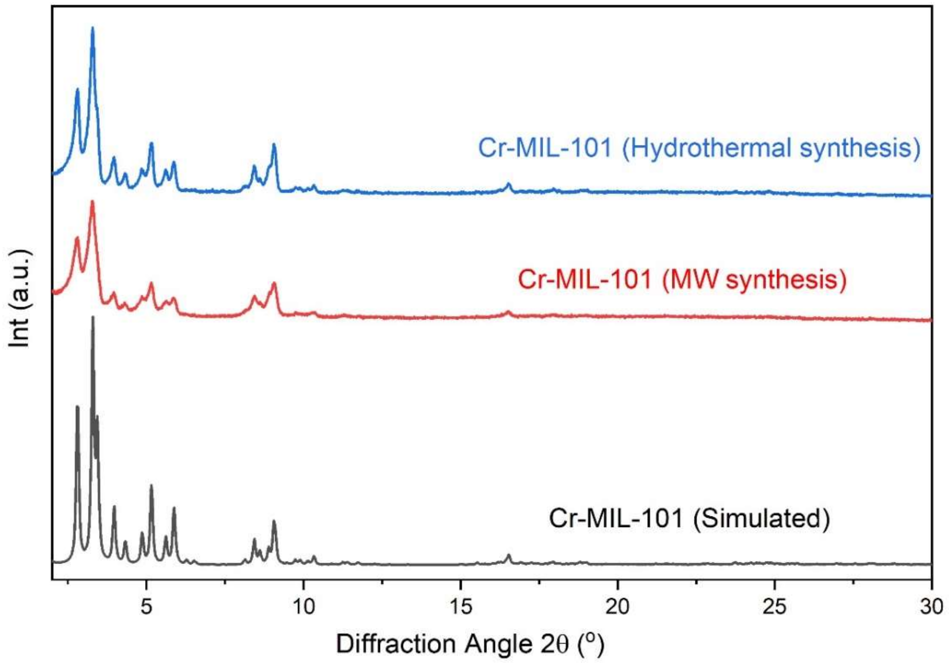

Figure S13. Comparison of the experimental PXRD patterns of Cr-MIL-101 synthesized using microwave (MW) synthesis and Cr-MIL-101 prepared via hydrothermal synthesis with a simulated pattern of Cr-MIL-101. An appreciable broadening of the diffraction peaks was observed for the sample prepared via MW synthesis, which we attribute to the formation of small MOF crystallites.

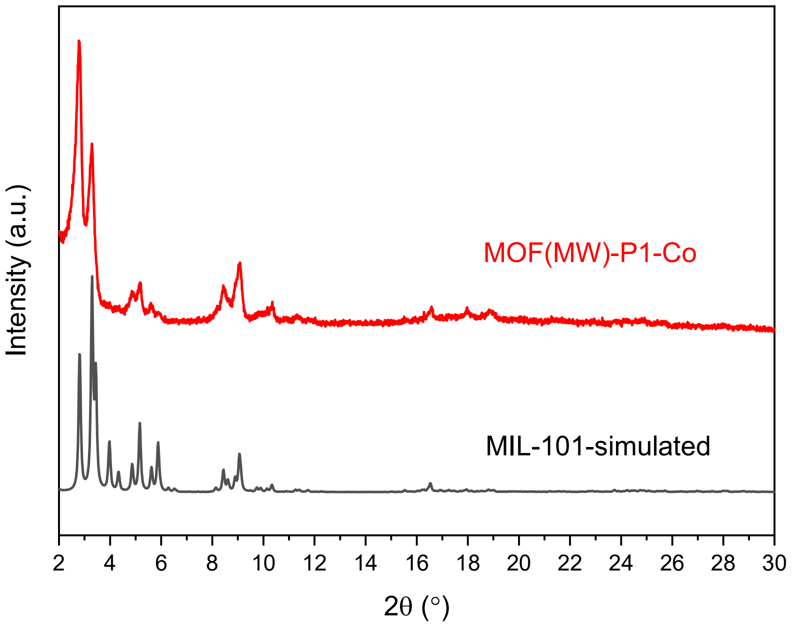

Figure S14. Comparison of the PXRD pattern of MOF(MW)-**P1**-Co with a simulation of Cr-MIL-101.

5.3 SEM Images and Energy Disperse X-ray Spectroscopy

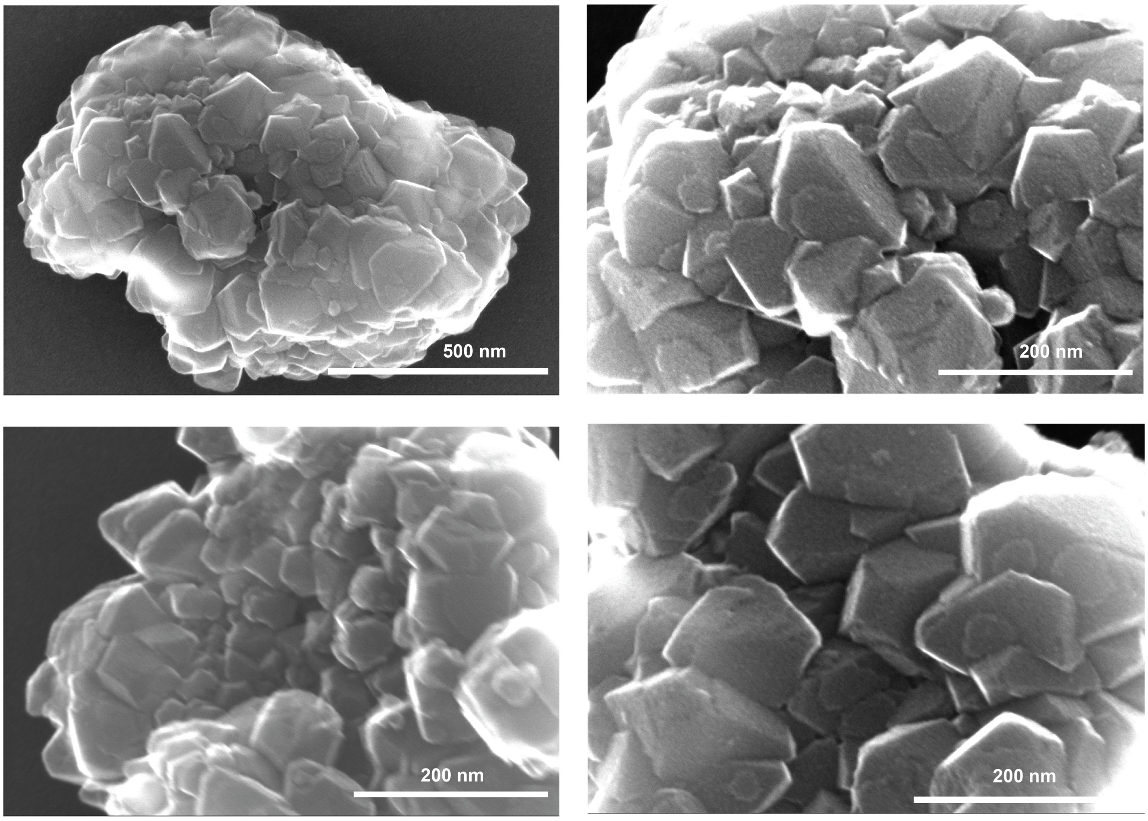

Figure S15. SEM images of Cr-MIL-101 synthesized using a microwave-based synthesis. Unlike the regular octahedra with an average longest dimension of around 300 nm obtained in the hydrothermal synthesis, Cr-MIL-101 (MW synthesis) consists of substantially smaller crystallites with a more irregular morphology.

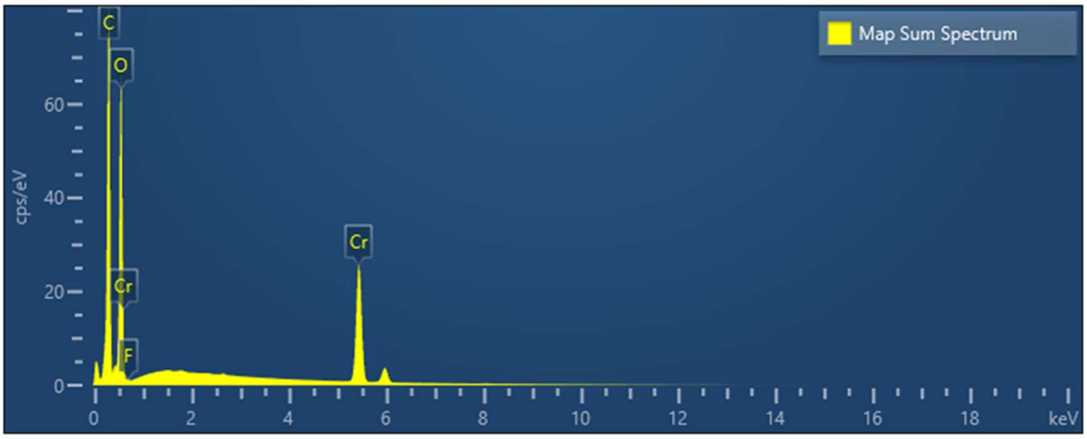

Figure S16. EDX spectrum of MIL-101-**F/OH**. The elements detected, in atomic percent, were Cr (30.97%), O (68.06%), F (0.97%).

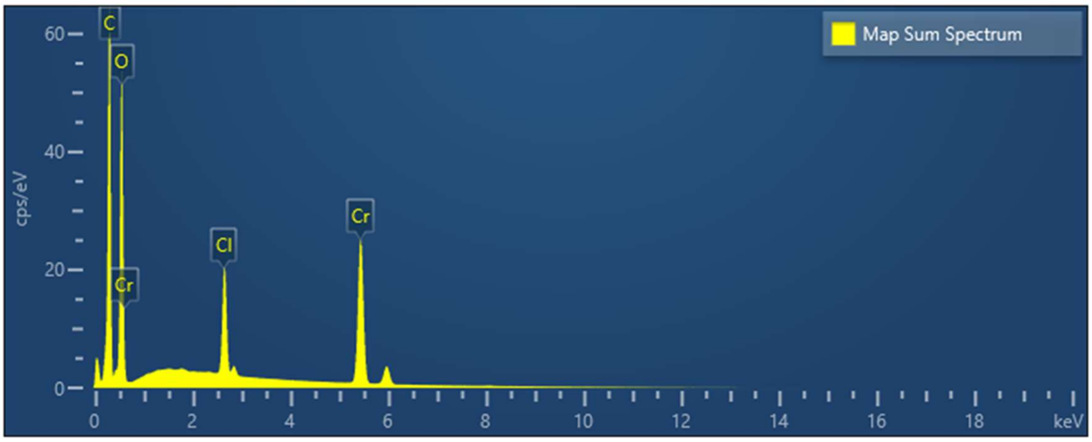

Figure S17. EDX spectrum of MIL-101-**Cl.** The elements detected, in atomic percent, were Cr (26.48%), O (65.45%), Cl (8.07).

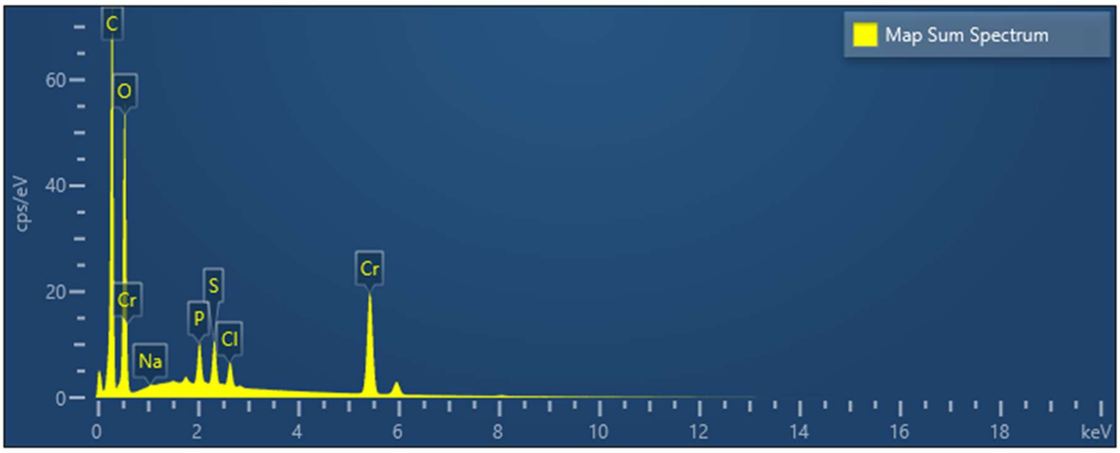

Figure S18. EDX spectrum of MOF-**P1**. The elements detected, in atomic percent, were Cr (21.65%), O (68.17%), Cl (2.27%), P (3.64%), Na (0.31%), S (3.95%).

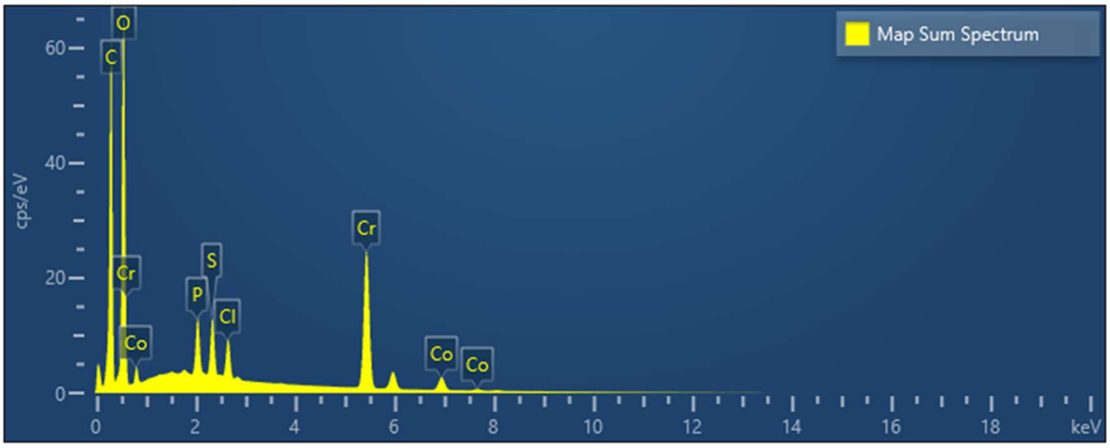

Figure S19. EDX spectrum of MOF-**P1**-Co. The elements detected, in atomic percent, were Cr (20.62%), O (65.70%), P (3.65%), S (3.70%), Cl (2.62%), Co (3.72%).

5.4 Gas Sorption

Argon sorption isotherms, pore size distributions, BET surface area values and pore volumes of MOF-**P1** samples containing different average loadings of **P1** are provided in the main text. The original experimental data along with meta data is provided for all other samples in the form of adsorption information (aif) files.

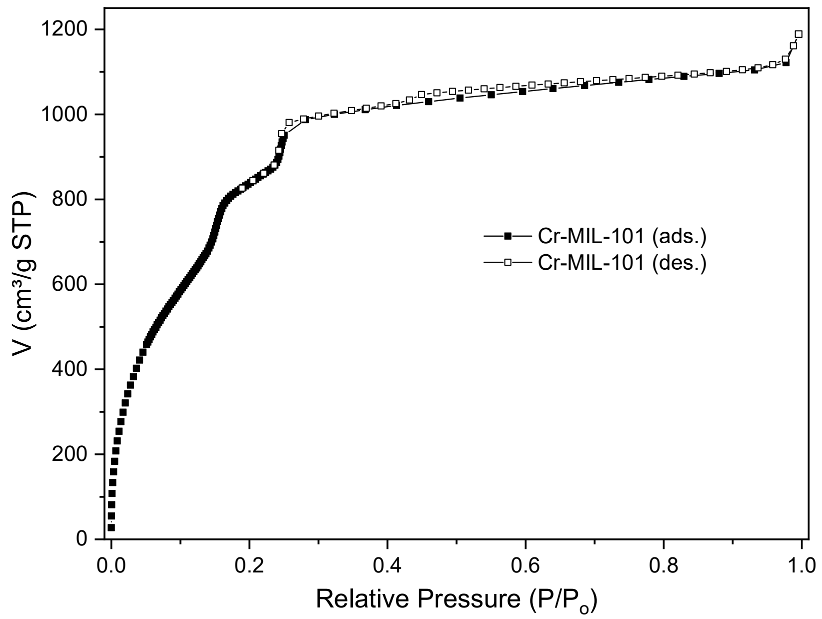

Figure S20. Ar sorption isotherm of Cr-MIL-101 synthesized using microwave (measured at 87 K). Based on the data, a BET surface area of 2570 $m^2 \cdot g^{-1}$ and total pore volume (1.44 $cm^3 \cdot g^{-1}$) was determined.

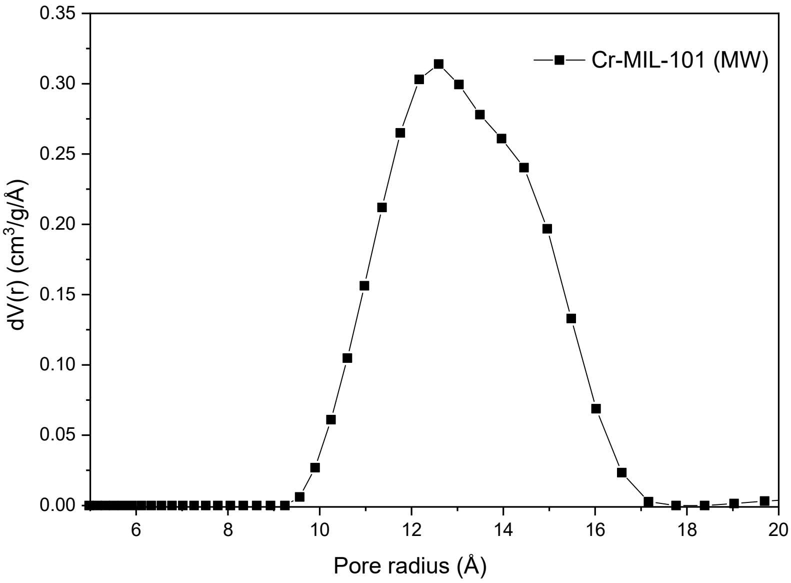

Figure S21. Pore size distribution of MIL-101(MW) derived from argon adsorption data.

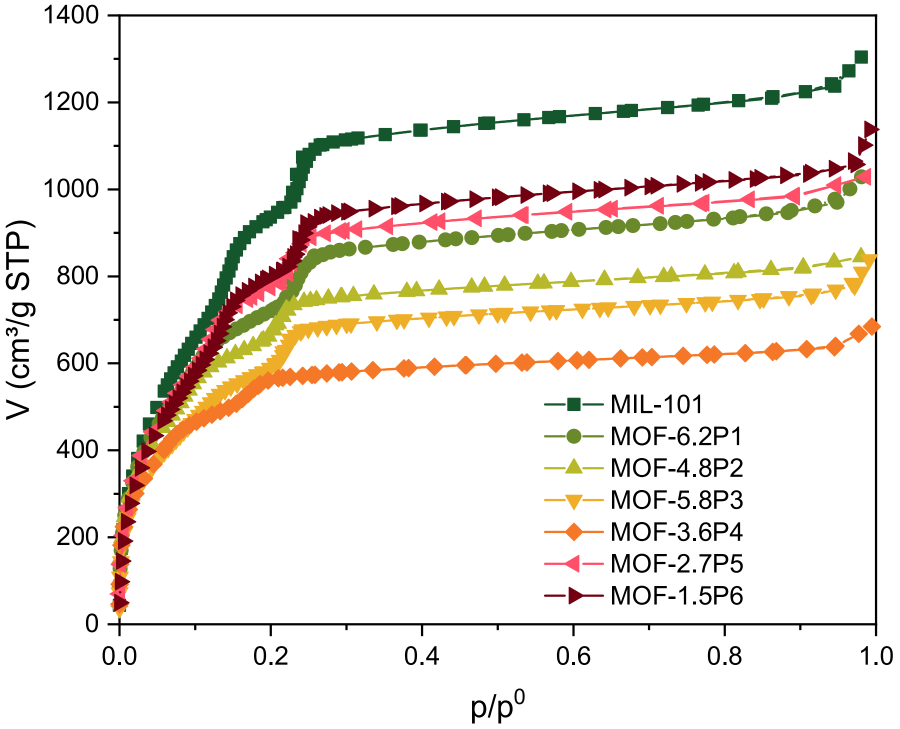

Figure S22 Comparison of the argon adsorption isotherms of MOF-**P** containing the phosphines **P1-P6.**

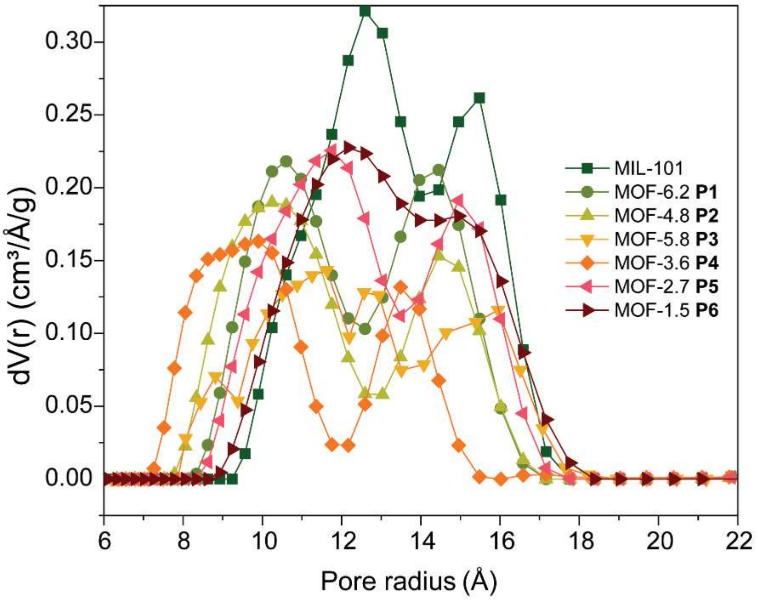

	Surface area ($m^2 \cdot g^{-1}$)	Pore volume ($cm^3 \cdot g^{-1}$)
MIL-101	2895	1.66
MOF-6.2 **P1**	2460	1.31
MOF-2.6 **P2**	2522	1.07
MOF-4.3 **P3**	2012	1.06
MOF-6.2 **P4**	1843	0.87
MOF-6.2 **P5**	2566	1.31
MOF-6.2 **P6**	2548	1.44

Figure S23 Comparison of the pore size distribution derived from argon adsorption data, surface area values and pore volumes of MOF-**P** containing the phosphines **P1-P6.**

The cobalt-carbonyl-containing samples could not be fully activated due to their thermal instability. In attempt to remove the majority of solvent molecules in the MOF pore without causing decomposition of the cobalt complexes that reside within the pores, we carried out a mild activation procedure. Specifically, MOF-P-Co was washed with toluene prior to repeated solvent exchange with pentane. The resulting sample was subjected to the dynamic vacuum provided by a turbomolecular pump for 12 h at 25 °C. After the activation was complete, the characteristic CO stretches of the pore-confined cobalt carbonyl complex could still be detected. The material in question was used for the argon sorption experiments shown in Fig. S25-S27. However, TGA data for MOF-P-Co subjected to the mild activation protocol described indicates that solvent molecules remain trapped within the pores, so that the argon sorption data presented underestimates the real pore volumes and surface values of the samples.

Notably, when MOF-P-Co was subjected to the standard activation conditions for Cr-MIL-101 (outgassing at 150 °C), no CO vibrations were observed for the resulting material (Fig. S24). MOF-P-Co that was activated at 150 °C showed no activity in hydroformylation catalysis, which indicates that the presence of the carbonyl ligands within the material is crucial for the catalytic activity of MOF-P-Co in hydroformylation.

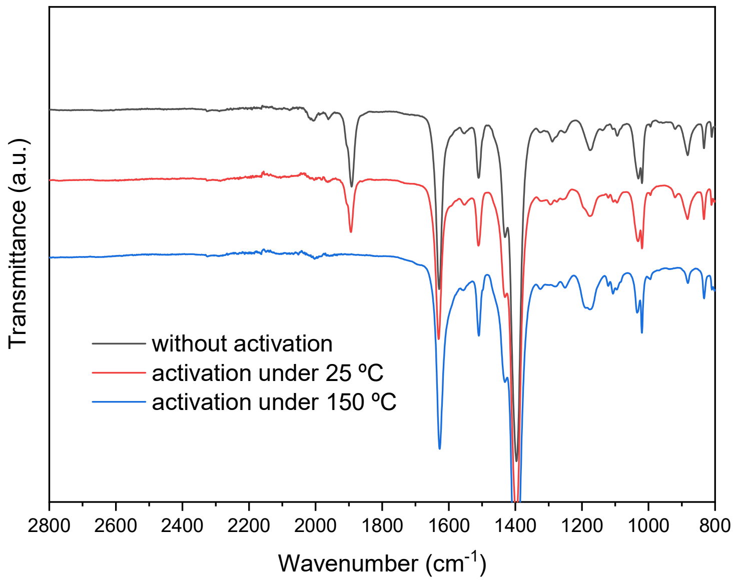

Figure S24. Comparison of ATR-IR spectra of MOF-**P1**-Co samples with different activated condition. After treatment at 150 °C, no CO stretching vibrations (2100cm^{-1} – 1850 cm^{-1}) could be observed.

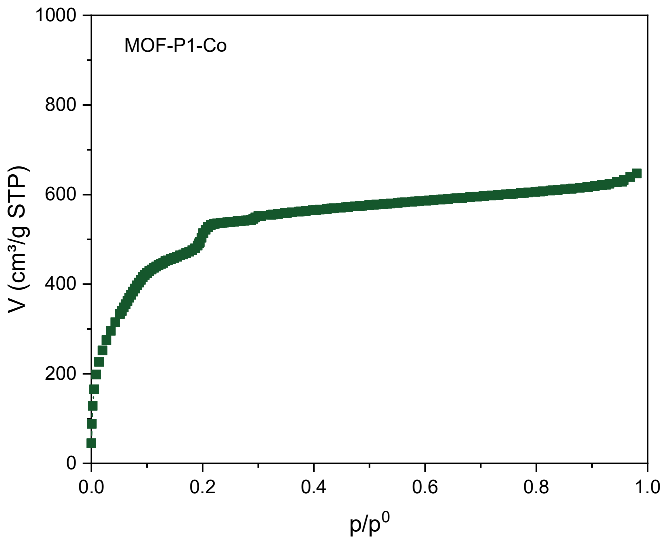

Figure S25. Argon adsorption isotherm of MOF-**P1**-Co. Based on the data, a BET surface area of 1870 m^2 · g^{-1} and total pore volume (0.82 cm^3 · g^{-1}) was determined.

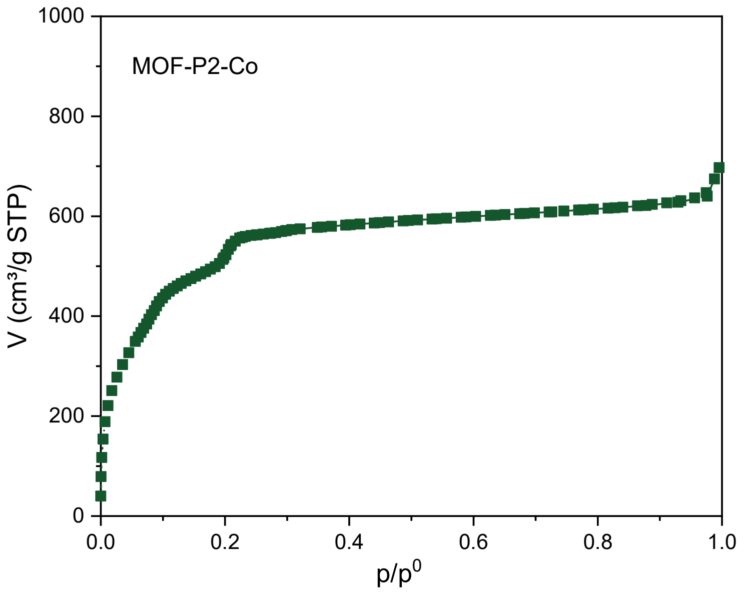

Figure S26. Argon adsorption isotherm of MOF-**P2**-Co. Based on the data, a BET surface area of 1988 $m^2 \cdot g^{-1}$ and total pore volume (0.83 $cm^3 \cdot g^{-1}$) was determined.

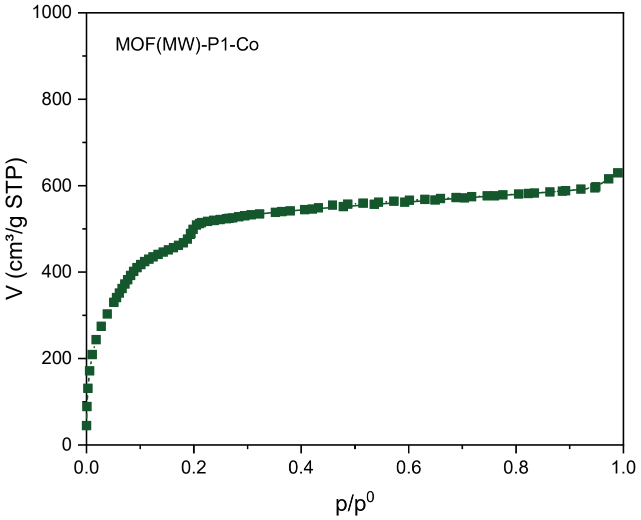

Figure S27. Argon adsorption isotherms of MOF(MW)-**P1**-Co. Based on the data, a BET surface area of 1801 $m^2 \cdot g^{-1}$ and total pore volume (0.79 $cm^3 \cdot g^{-1}$) was determined.

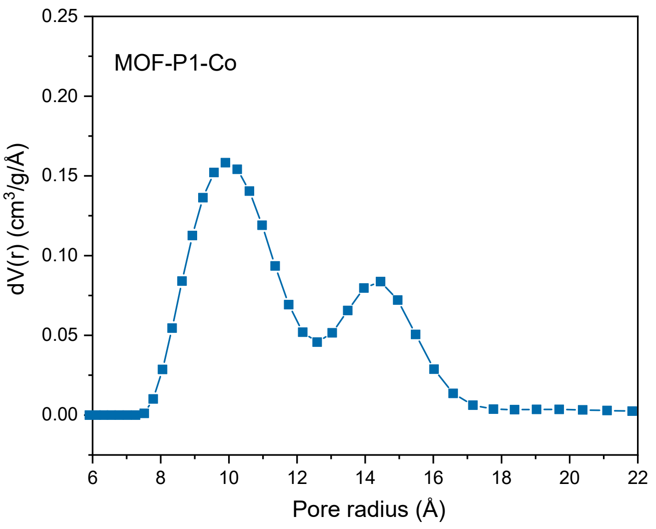

Figure S28. Pore size distribution of MOF-**P1**-Co derived from argon adsorption data. The half pore widths of the two types of mesoporous cages are 9.9 Å and 14.5 Å.

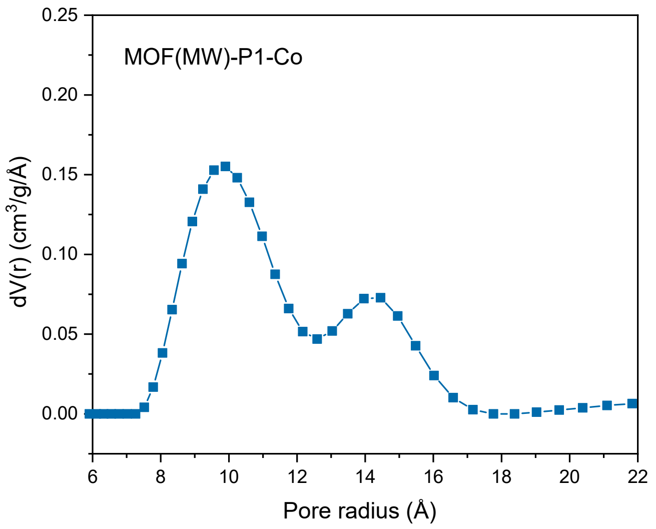

Figure S29. Pore size distribution of MOF(MW)-**P1**-Co derived from argon adsorption data. The half pore widths of the two types of mesoporous cages are 9.9 Å and 14.5 Å.

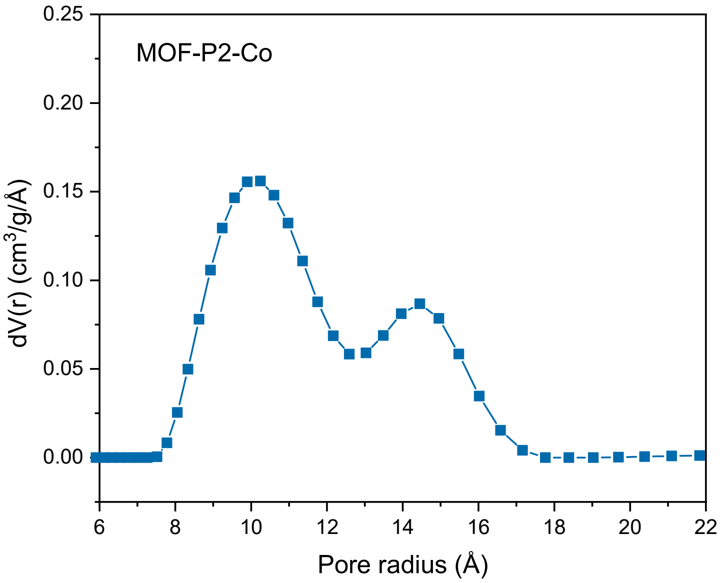

Figure S30. Pore size distribution of MOF-**P2**-Co derived from argon adsorption data. The half pore widths of the two types of mesoporous cages are 10.2 Å and 14.5 Å.

5.5 Thermal Gravimetric Analysis

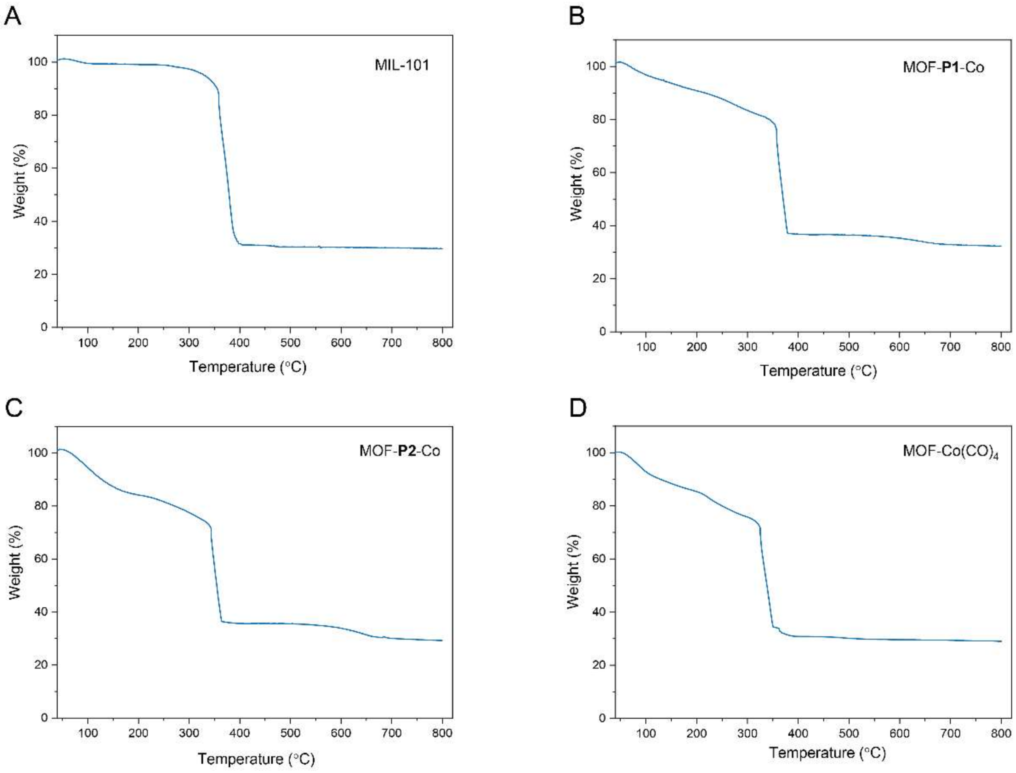

Figure S31. Comparison of the TGA traces of MIL-101 (**A**)**,** MOF-**P1**-Co (**B**), MOF-**P2**-Co (**C**) and MOF-$Co(CO)_4$ (**D**) collected under air. All samples were not activated. MIL-101 exhibited thermal stability in air up to 300 °C before decomposition occurred, whereas all three cobalt-containing samples showed a notable mass loss starting from 50 °C. A rigorous assignment of the mass loss to the loss of carbonyl ligands from the MOF-encapsulated cobalt carbonyl complexes was not possible because the low temperature stability precluded rigorous activation. The low-temperature mass loss is thus due to both the decomposition of the pore-encapsulated cobalt carbonyls and residual solvent molecules that remain present in the pores.

Additional TGA experiments with activated MOF samples (see below) showed that the mild activation conditions required to ensure that the pore-contained cobalt carbonyl complexes remain stable were not able to fully remove solvent molecules from the pores. The low-temperature mass loss (between 30 °C and 100 °C) corresponds to 15% of the total weight of the sample, whereas the loss of the carbonyl ligands associated with cobalt would only correspond to a mass loss of 4.7 wt%, so that both solvent molecules and carbonyl ligands are lost in a low temperature step that could not be resolved further.

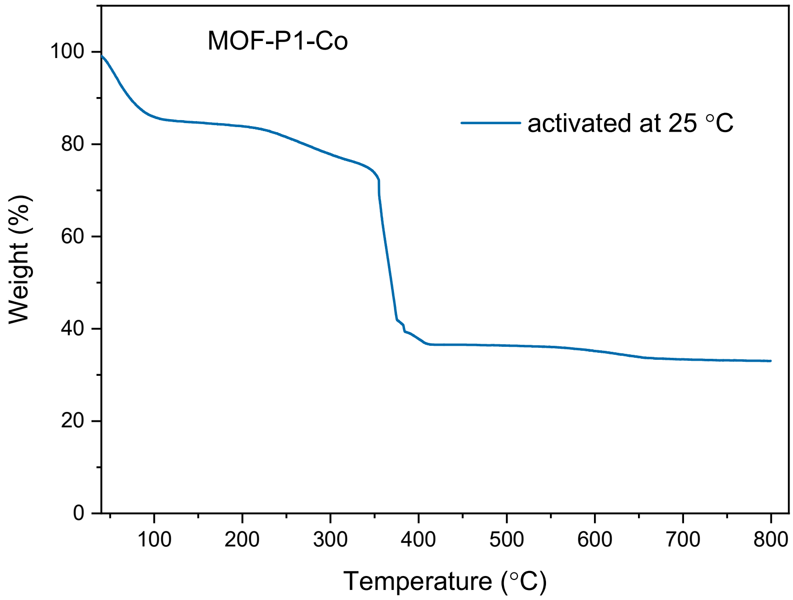

Figure S32. TGA trace of MOF-**P1**-Co activated at 25 °C.

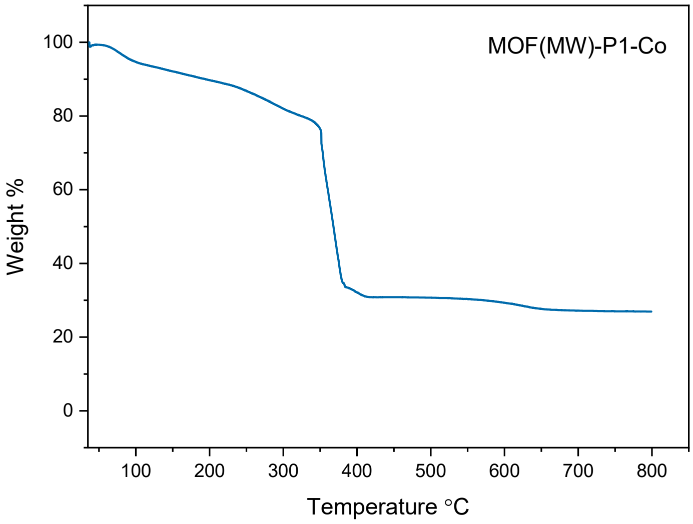

Figure S33. TGA trace of MOF(MW)-**P1**-Co activated at 25 °C.

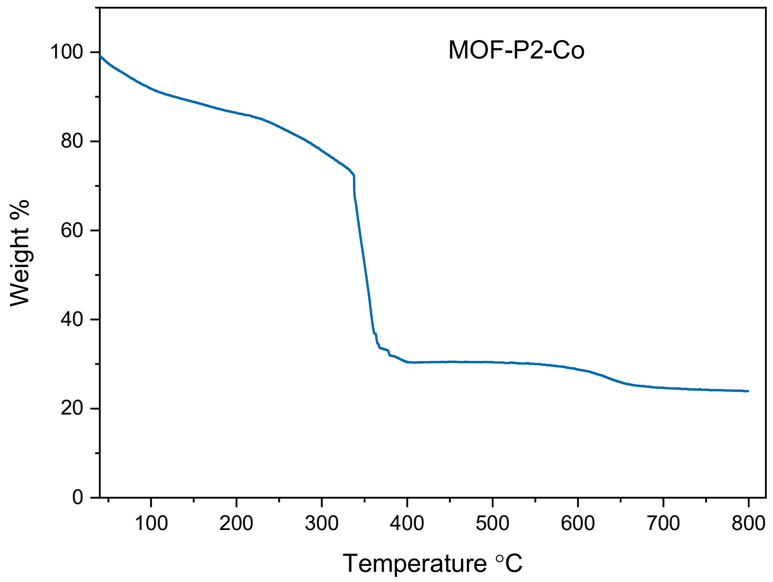

Figure S34. TGA trace of MOF-**P2**-Co activated at 25 °C.

5.6 NMR Spectroscopy

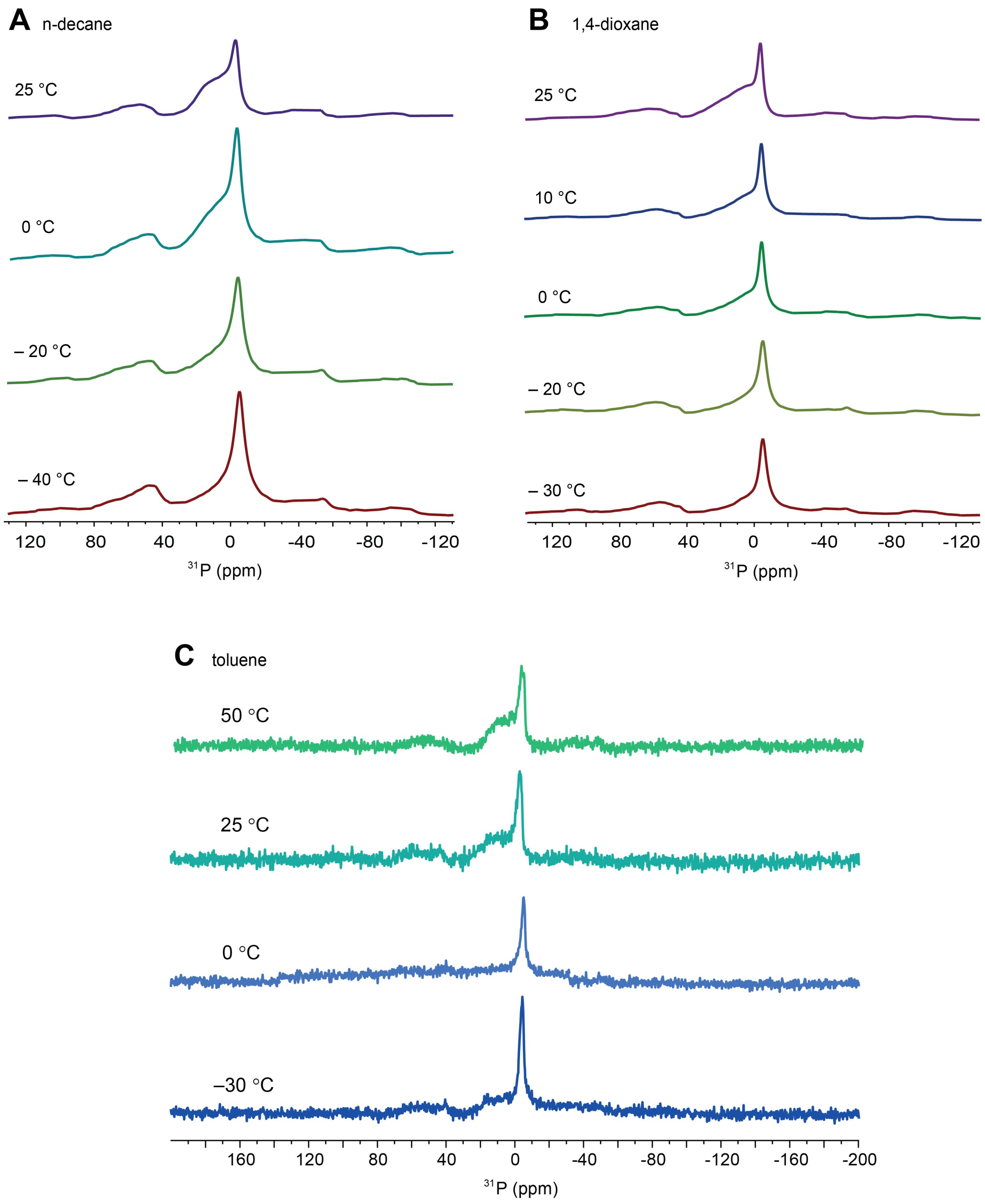

Figure S35. Mobility Assessment of MOF-**P1** by variable-temperature ^{31}P SS-NMR spectroscopy in the presence **A** n-decane, **B** 1,4-dioxane, and **C** toluene.

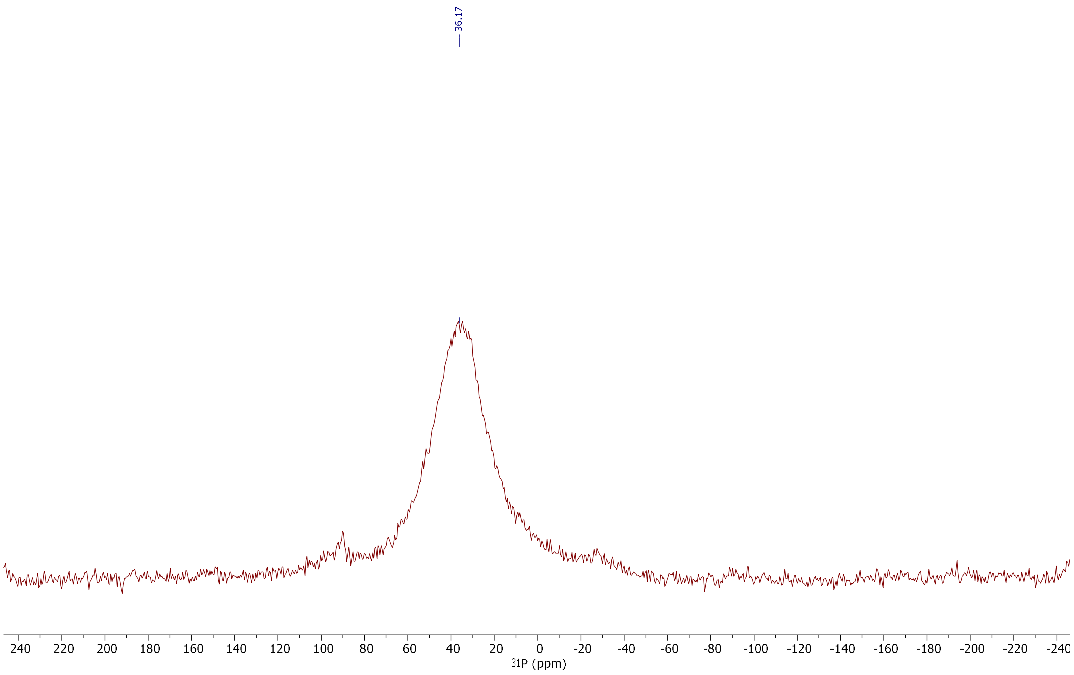

Figure S36. Solid-state ^{31}P NMR spectrum of MOF-**P1** after air exposure for 24 h. Exposure to ambient air leads to oxidation of the phosphorus center, as evidenced by the appearance of a resonance centered at 36.2 ppm. Signals corresponding to oxidized phosphine ligands were not observed in solid-state NMR data for MOF-P or MOF-P-Co samples, which were handled under inert atmosphere.

To aid in the assignment of ^{59}Co NMR spectra, we relied on theoretical calculations in addition to comparison with literature data. To establish a theoretical basis for the NMR parameters, we first performed density functional theory (DFT) calculations using the CASTEP program with the GIPAW method on the crystal structure. Guided by these DFT results, we then manually refined the experimental spectrum using the 'sola' module in TopSpin (Bruker), adjusting parameters to match the observed linewidth and peak positions (Figure 3B in main manuscript).

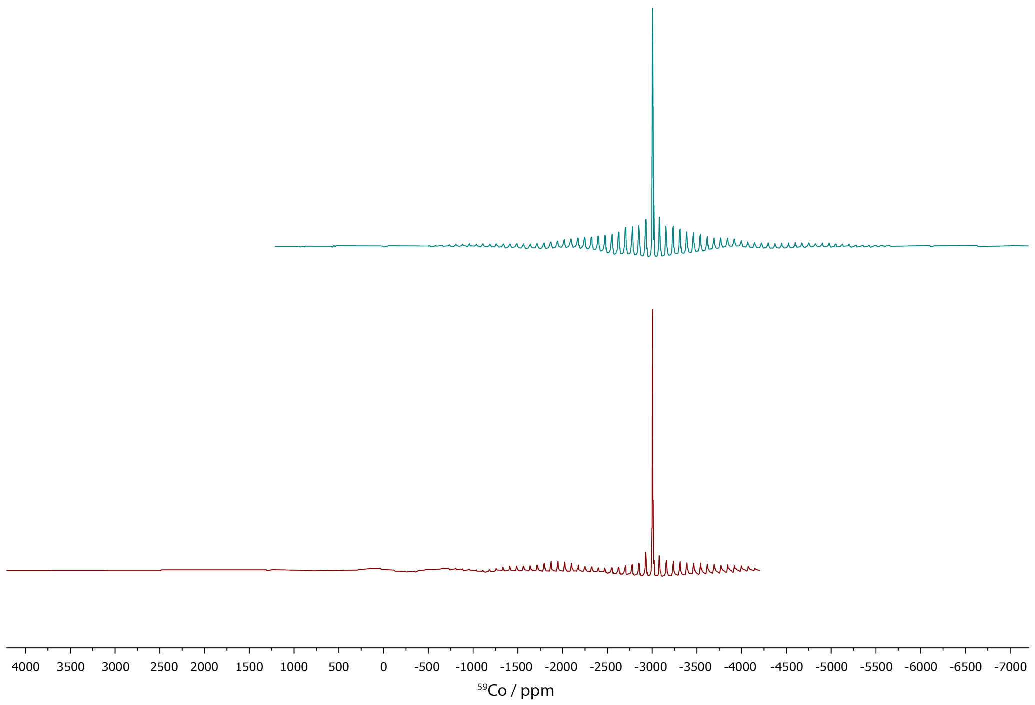

Figure S37. Comparison of ^{59}Co spectra of $[Co(CO)_3(PPh_3)_2]^+[Co(CO)_4]^-$ that were collected with different spectral widths under 9 kHz MAS. While two magnetically inequivalent cobalt nuclei are present in $[Co(CO)_3(PPh_3)_2]^+[Co(CO)_4]^-$, the low symmetry of the cobalt center in the cationic fragment precludes observation of a ^{59}Co resonance unless extremely long acquisition times are used (see main manuscript). The depicted signal corresponds to the ^{59}Co resonance of the $Co(CO)_4$ anion for which cobalt occupies a high-symmetry environment.

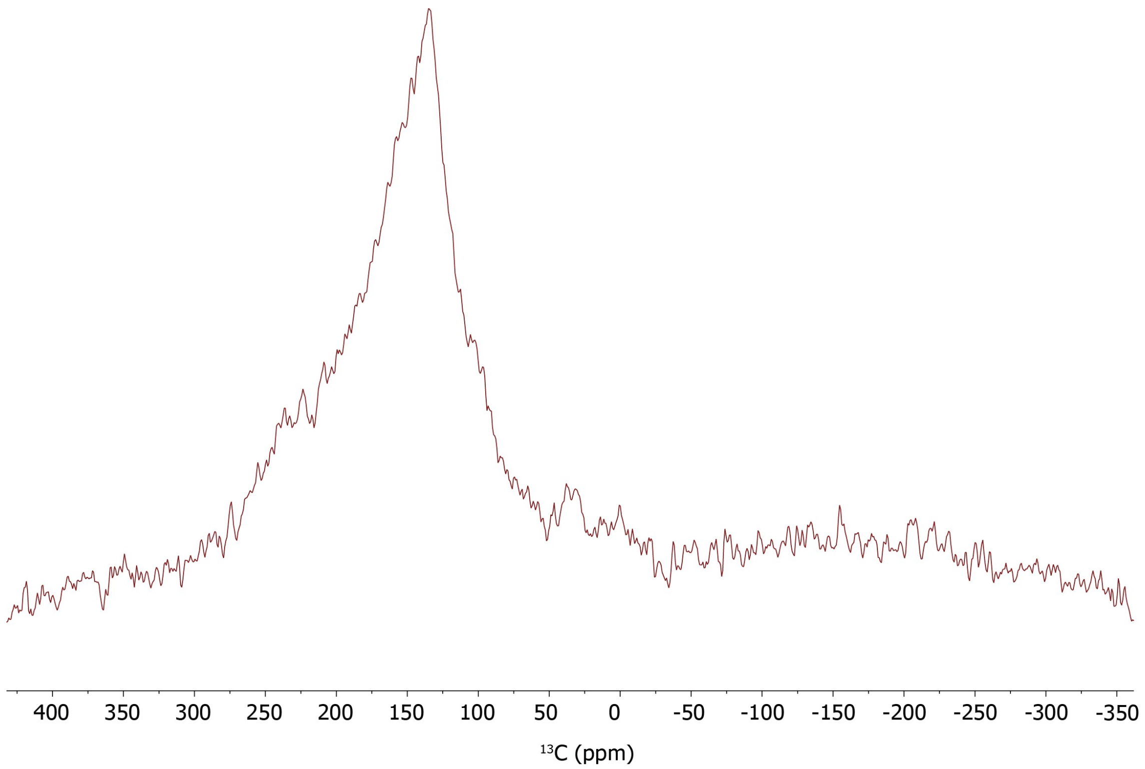

Figure S38. Solid-state ^{13}C NMR spectrum of MOF-**P1**.

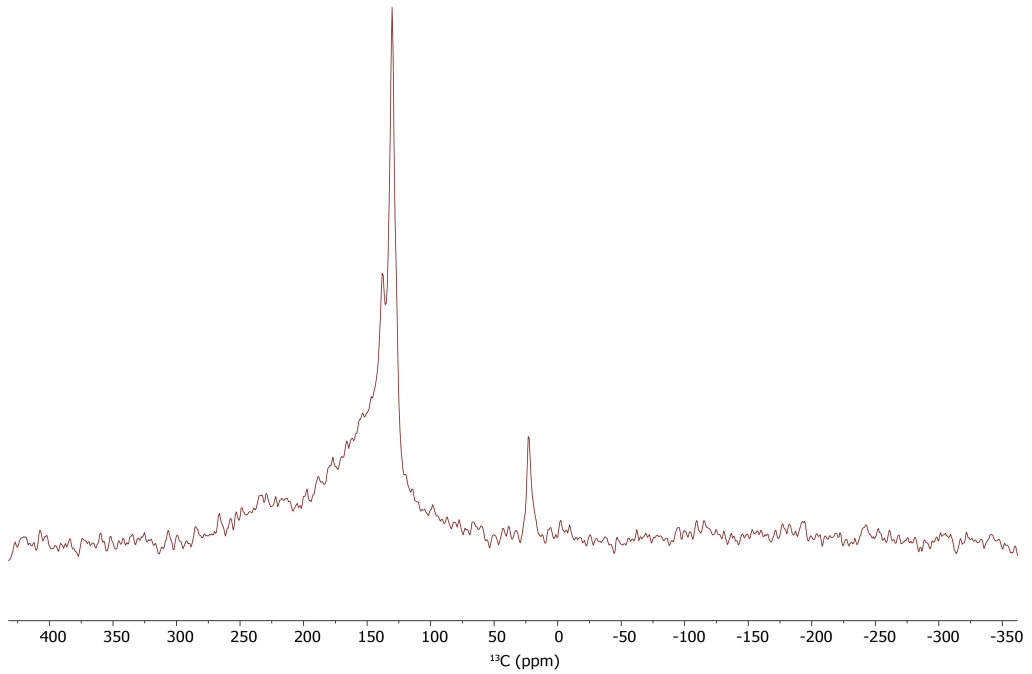

Figure S39. Solid-state ^{13}C NMR spectrum of MOF-**P1**-Co. The sample was prepared in toluene and was not rigorously activated prior to the measurement (see discussion in sorption and TGA sections). Sharp signals corresponding to toluene (130 ppm, 137 ppm and 23 ppm) inside the MOF cage were thus clearly apparent in the spectrum.

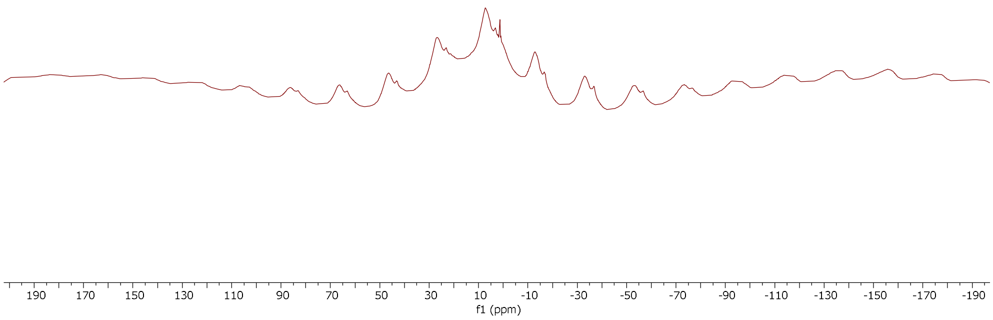

Figure S40. Solid-state ^{1}H NMR spectrum of MOF-**P1.**

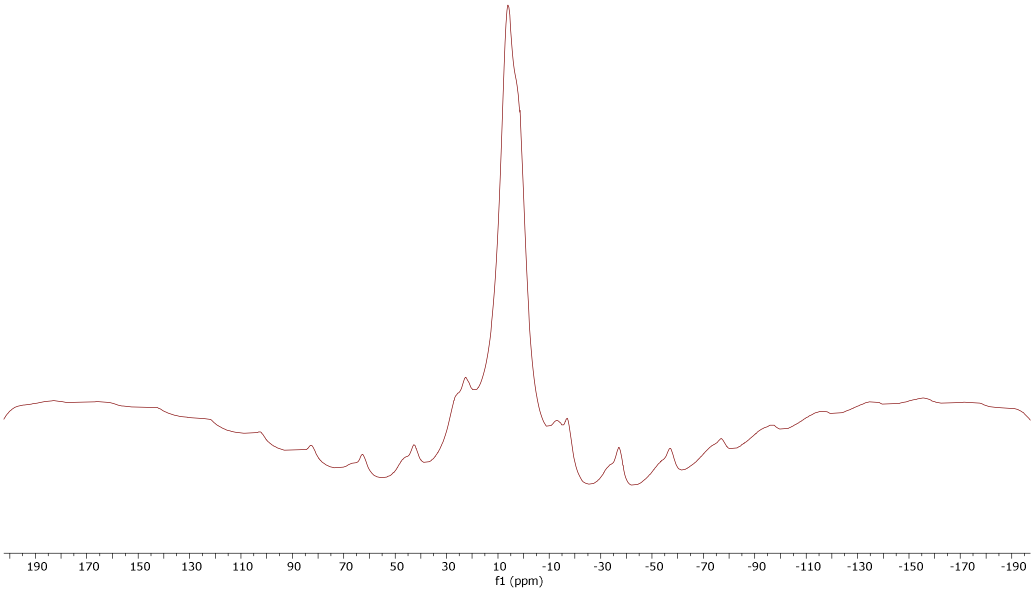

Figure S41. Solid-state ^{1}H NMR spectrum of MOF-**P1**-Co.

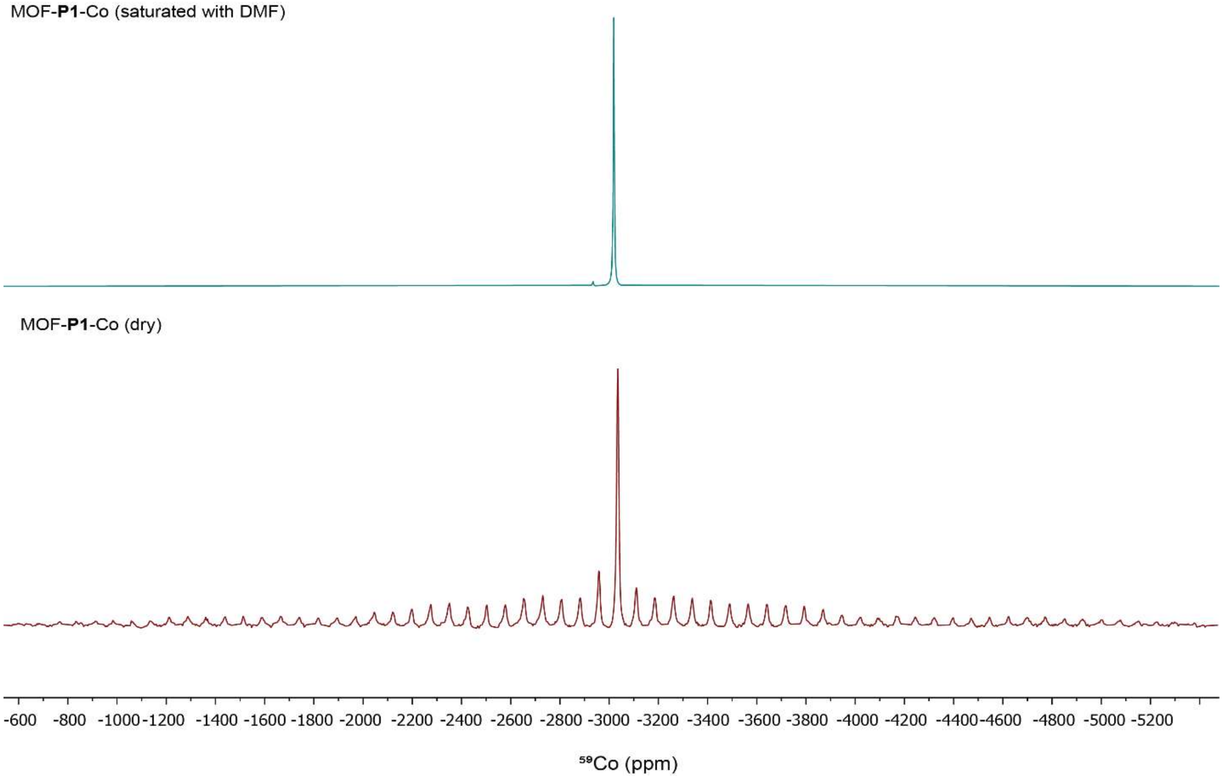

Figure S42. Comparison of ^{59}Co NMR spectra of dry MOF-**P1**-Co (bottom) and MOF-**P1**-Co saturated with DMF (top). The disappearance of spinning side bands for the spectrum collected with a MOF sample in which the pores are filled with DMF indicates that the observed cobalt center ($[Co(CO)_4]$ anion) has a high degree of mobility.

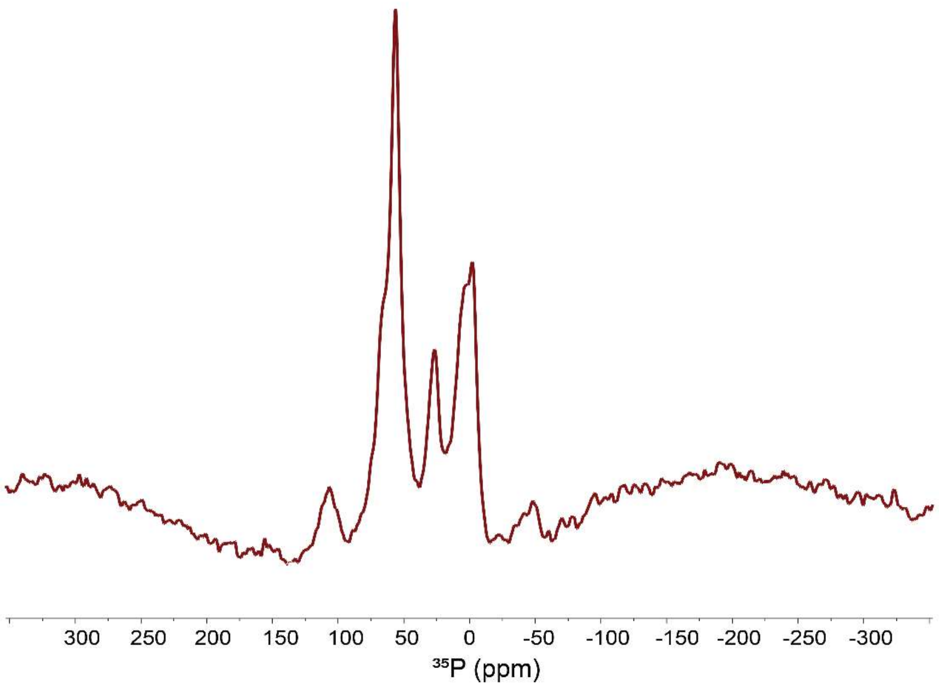

Figure S43. Solid-state ^{31}P NMR spectrum of pristine MOF-**P1**-Co with DMF-solvated pores. The emergence of a sharp peak was observed for the ^{31}P signal derived from [Co(CO)$_3$(**P1**)$_2$]$^-$ when the pores were filled with DMF, indicating that [Co(CO)$_3$(**P1**)$_2$]$^-$ becomes mobile within the MOF super-cage in the presence of a polar solvent. The coordinating solvent also led to the partial displacement of phosphine ligands from the Co^{I} center so that an additional sharp signal is apparent around 0 ppm, which corresponds to free **P1**. In addition to the sharp signals, a residual signal with spinning side bands is also present, so that not all [Co(CO)$_3$(**P1**)$_2$]$^-$ complexes were mobile during the experiment. Since partial decomposition of the species of interest had been observed even in the presence of limited amounts of DMF, however, the addition of increased amounts of DMF was not attempted.

5.1 Infrared Spectroscopy

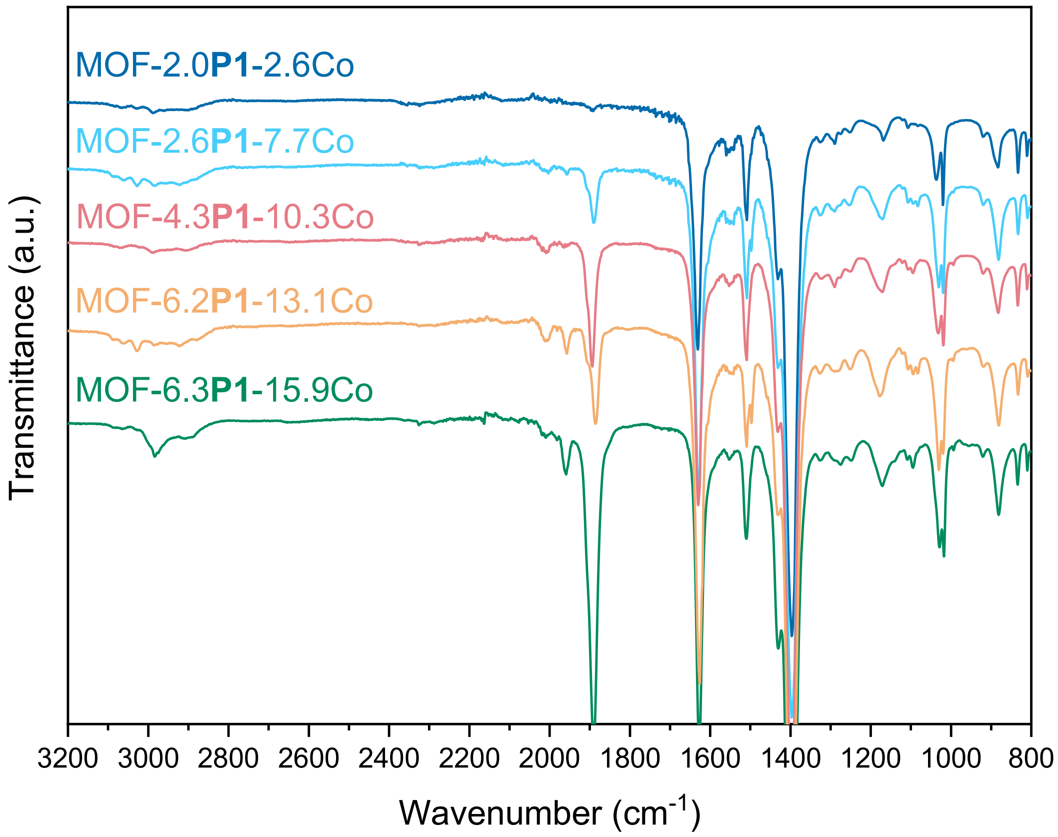

Figure S44. Comparison of ATR-IR spectra of MOF-**P1**-Co samples with different **P1** and Co contents.

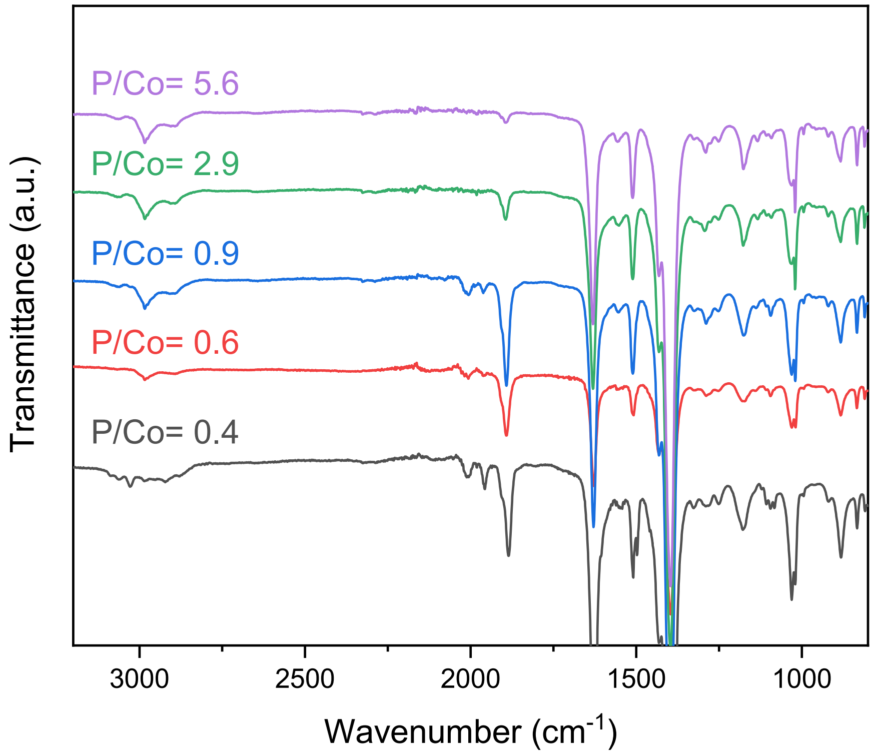

Figure S45. Comparison of ATR-IR spectra of MOF-**P1**-Co with different P/Co ratios. The amount of **P1** present was kept constant at an average of 6.2 **P1** ligands per MIL-101 cage.

6 Catalysis

6.1 Standard Experimental Procedure for Heterogeneous Hydroformylation

The hydroformylation reaction of 1-hexene was carried out in a stainless steel autoclave with an internal volume of 45 mL. In an argon-filled glovebox, MOF-**P1**-Co (11.8 mg – corresponding to 0.50 mol% [Co]) and substrate (0.96 mmol, 1.00 equiv) was placed inside the autoclave before toluene (10 mL) was added. Dodoceane (0.10 mL) was added as an internal standard to determine the mass balance. Then the autoclave was sealed, removed from the glovebox, and filled with CO (20 bar) and H_2 (20 bar) before being heated at 170 °C with continuous stirring (250 rpm). Please note that the temperature indicated in tables and figures refers to the set-point of the heating apparatus, and that the internal temperature inside the autoclave remained around 5 °C below the indicated set-point throughout the reaction. After completion of the reaction, the reaction mixture was allowed to cool to room temperature before the catalyst was removed by filtration and the resulting filtrate was analyzed by GC, GC-MS and/or NMR to determine the conversion, selectivity and mass balance. The response to changes in reaction conditions is described in subsequent figures. Please see Fig. S51 for any deviations from the standard reaction conditions for different substrates.

6.2 Optimization of Reaction Conditions

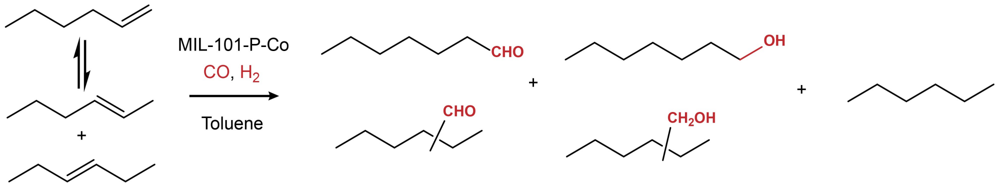

6.2.1 Reaction Temperature

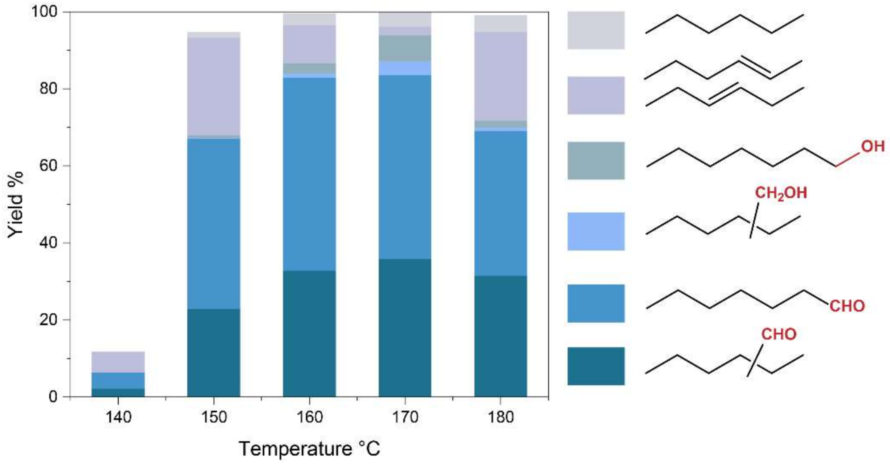

Figure S46. Reaction temperature optimization from 140 °C to 180 °C. Reaction conditions: catalyst: MOF-**P1**-Co corresponding to 1 mol% Co; 0.96 mmol 1-hexene (1.00 equiv.), 10 mL toluene, 6 h reaction time, 40 bar syngas (1 : 1 ratio of CO and H_2).

6.2.2 Total Pressure and CO / H_2 Ratio

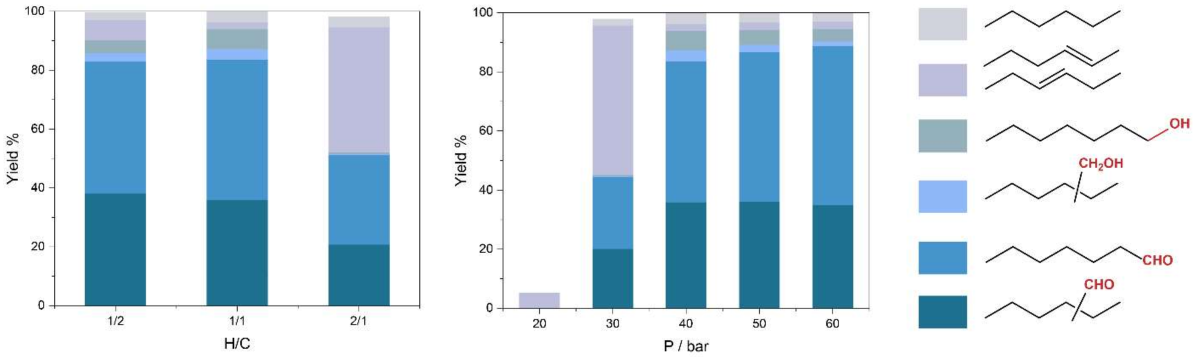

Figure S47. Optimization of the ratio of H_2 and CO (shown on x-axis of left figure as H/C) and the total pressure. Reaction conditions: MOF-**P1**-Co corresponding to 1 mol% Co; 0.96 mmol 1-hexene (1.00 equiv), 10 mL toluene, 6 h, 170 °C.

6.2.3 Reaction Time

An extension of the reaction time reduces the amount of isomerized olefins obtained and increases the fraction of oxo-products obtained. The isomerized olefins are thus competent substrates for hydroformylation and the reaction conditions explored in Fig. 5A and Fig. 5C for catalyst comparisons and recycling of the heterogeneous catalyst correspond to kinetically limited reaction conditions under which the full accessible conversion has not yet been achieved.

Table S7. Effect of reaction time on product distribution.[a]

Reaction time/h	Hexane %	Isomerization%	i-aldehyde %	n-aldehyde%	i-alcohol%	n-alcohol%
6	3	21	30	43	-	2
12	5	4	38	51	1	2

[a]Reaction conditions: MOF-**P1**-Co corresponding to a loading of 0.5 mol% Co; 0.96 mmol 1-hexene, 10.0 mL toluene, 170 °C, 40 bar syngas.

6.3 Optimization of Catalyst Composition

6.3.1 Effect of **P1** Loading

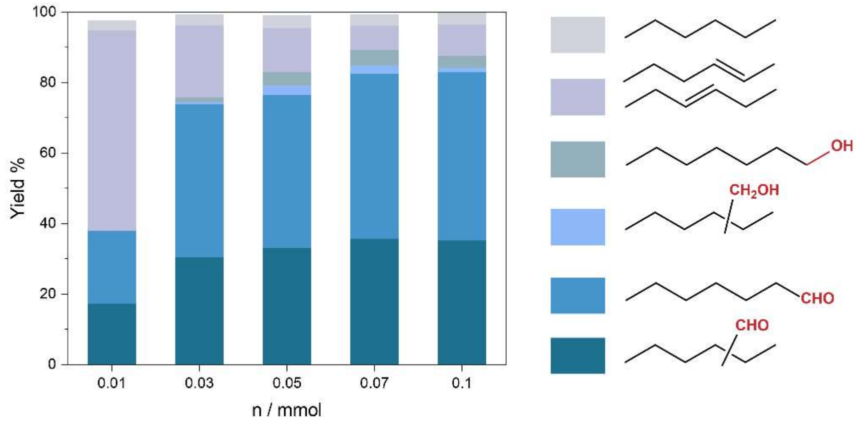

Figure S48. Effect of different average numbers of **P1** ligands per MOF cage on product distribution in hydroformylation. The x-axis lists the amount of **P1** that was used to prepare the heterogeneous ligand in question (see experimental section). Interestingly, higher phosphine loadings (corresponding to an average content of 6.2 – 6.4 **P1** ligands per MOF cage) gave rise to improved performance. Reaction conditions: 0.5 mol% Co; 0.96 mmol hexene, 10 mL toluene, 6 h, 170 ^{o}C, 0.10 mL dodecane, 40 bar syngas.

6.3.2 Effect of P / Co Ratio

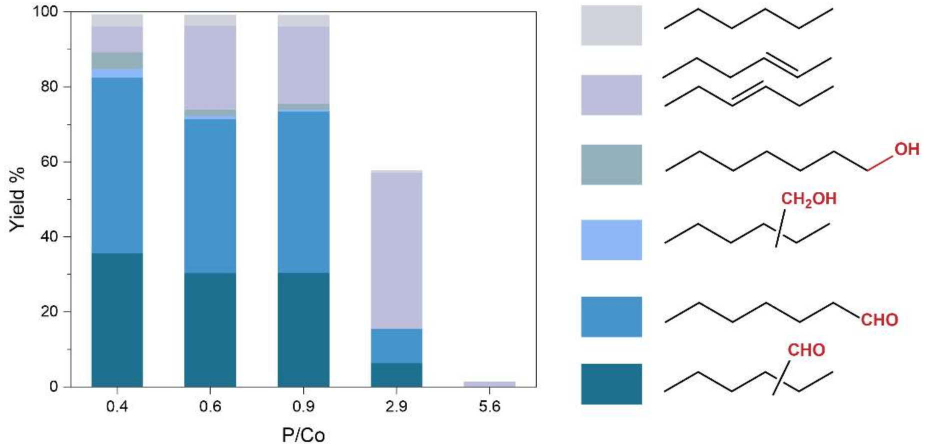

Figure S49. Effect of the ratio of **P1** and Co in MOF-**P1**-Co on product distribution in hydroformylation. Reaction conditions: 0.5 mol% Co; 0.96 mmol hexene, 10.0 mL toluene, 6 h, 170 ^{o}C, 40 bar syngas.

6.4 Control Reaction with Cr-MIL-101

To determine whether the isomerization of 1-hexene to internal olefins observed during hydroformylation reactions was (partially) promoted by Cr^{III} centers or potential defect sites in Cr-MIL-101, we carried out a control reaction (Table S8). When only the Cr-MIL-101 MOF host was present, no conversion of 1-hexene could be observed, so that the isomerization observed in the presence of MOF-**P1**-Co was promoted by cobalt, not chromium.

Table S8. Reaction products observed in the attempted hydroformylation of n-hexene catalyzed by either MOF-**P1**-Co or Cr-MIL-101.[a]

Catalyst	1-hexene (%)	Isomerized hexene (%)	i-aldehyde %	n-aldehyde %	i-alcohol (%)	n-alcohol (%)
MOF-**P1**-Co	3	21	30	43	-	2
MOF	100	-	-	-	-	-

[a]Reaction conditions: 11.8 mg catalyst; 0.96 mmol 1-hexene, 10.0 mL toluene, 170 °C, 40 bar syngas.

6.5 Kinetic experiments

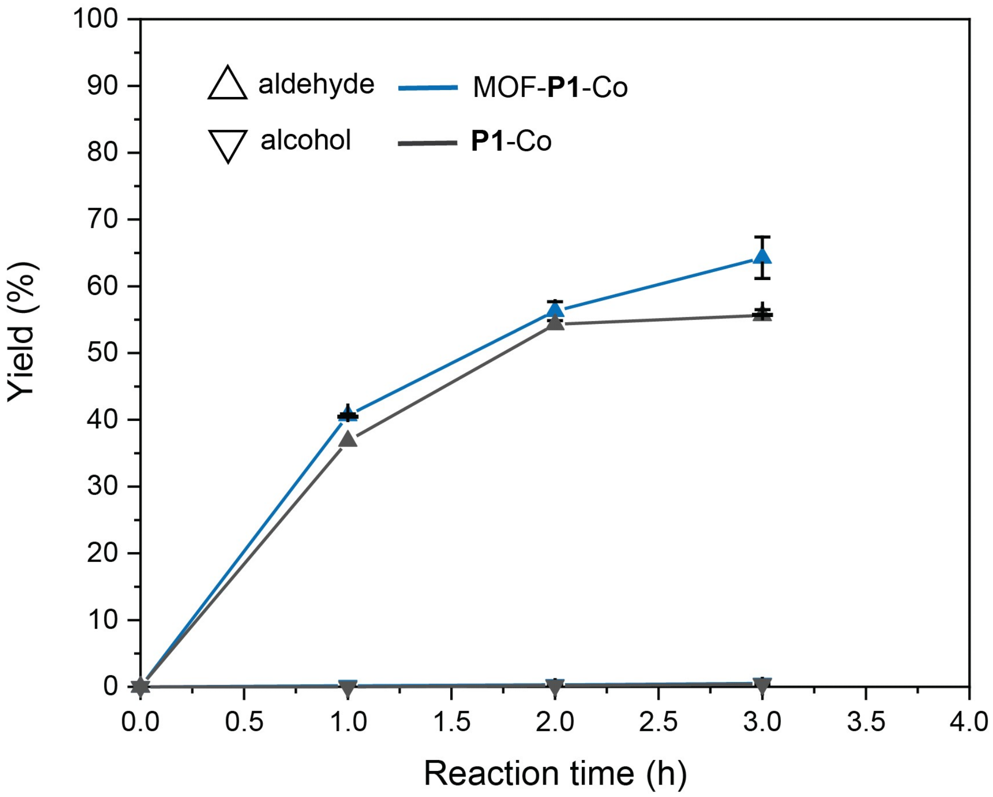

Figure S50. Comparison of kinetic profiles of 1-hexene hydroformylation catalyzed by MOF-**P1**-Co (blue) or and **P1**/Co (black). For the heterogeneous catalyst, two experiments were carried out for each time point and the range of yields of aldehyde products obtained is indicated. Reaction conditions: catalyst amount corresponding to 0.5 mol% Co; 0.96 mmol hexene, 10.0 mL toluene, 170 °C, 40 bar syngas. The product distribution and corresponding yields are tabulated in Table S9.

Table S9. Kinetic data of hexene hydroformylation reaction catalyzed by MOF-**P1**-Co and P1-Co.

Reaction Time/h	Cat.	Repetition	Hexane %	Isomerization %	i-aldehyde %	n-aldehyde %
1	P1-Co	1	2	53	15	22
1	MOF-**P1**-Co	1	2	51	17	24
		2	2	51	17	24
2	P1-Co	1	2	41	23	31
2	MOF-**P1**-Co	1	2	35	25	36
		2	2	38	24	33

3	P1-Co	1	2	42	22	31
		2	3	40	23	33
3	MOF-**P1**-Co	1	3	29	28	38
		2	3	33	26	36

6.6 Evaluation of Potential Mass Transfer Limitations with MOF(MW)-P1-Co

To evaluate whether mass transfer through the porous catalyst has a notable effect on reaction outcomes, Cr-MIL-101 with a substantially smaller crystallite size compared to the standard catalyst was prepared via a reported microwave-based synthesis protocol. Relevant PXRD, SEM, and sorption data are shown in Chapter 5. Since essentially identical product distributions were obtained after short reaction times for catalysts with different crystallite sizes (Table S10), mass transfer through the pore system does appear to affect reaction outcomes in the heterogeneous hydroformylation of 1-hexene.

Table S10. Comparison of product distributions observed for 1-hexene hydroformylation with small crystallite MOF(MW)-**P1**-Co and MOF-**P1**-Co.[a]

Catalyst	Hexane %	Isomerization%	i-aldehyde %	n-aldehyde%	i-alcohol%	n-alcohol%
MOF(MW)-**P1**-Co	2	50	17	25	0	0
MOF-**P1**-Co	2	51	17	24	0	0

[a]Reaction conditions: MOF-**P1**-Co or MOF(MW)-**P1**-Co corresponding to a loading of 0.5 mol% Co; 0.96 mmol 1-hexene, 10.0 mL toluene, 170 °C, 1 h, 40 bar syngas.

6.7 Substrate Scope

In our exploration of the substrate scope, we focused longer-chain terminal olefins and substrates containing an internal double bond, since efficient cobalt-catalyzed olefin isomerization permits an isomerization-hydroformylation sequence. The starting olefins are depicted in the main text while Fig. S51 shows the aldehyde products that were obtained along with deviations in the reaction conditions from those employed in the hydroformylation of 1-hexene.

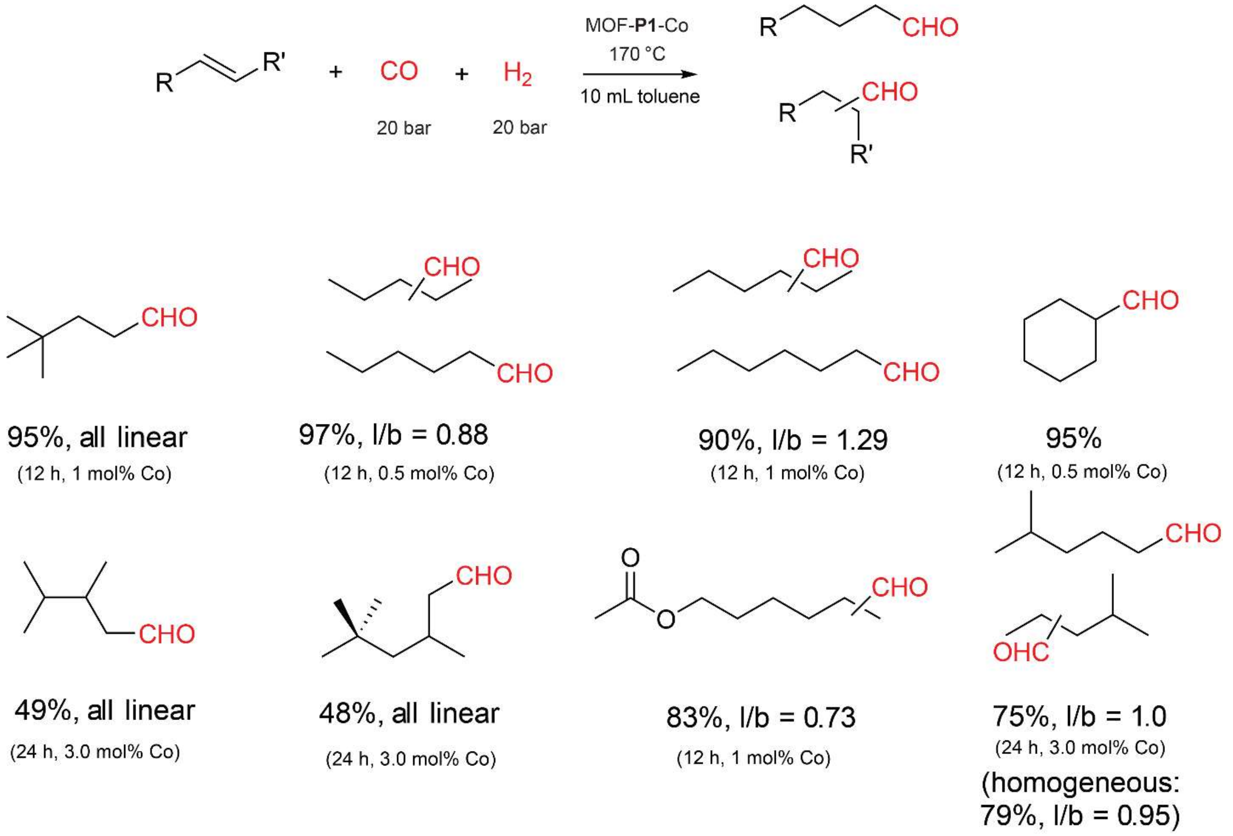

Figure S51 Substrate scope MOF-**P1**-Co hydroformylation catalysis. The catalyst loading and reaction time are indicated for all substrates. All other reaction conditions were identical to those described in the standard experimental procedure for 1-hexene.

6.8 Hydroformylation of Bulky Substrates

To evaluate whether bulky substrates could access the MOF cages for reaction, heterogeneous hydroformylation of cholesterol (largest dimension: 1.78 nm; see Fig. S52) was attempted and the results compared with a homogeneous control reaction. Since cholesterol exhibits a very high degree of local steric hindrance surrounding the double bond the catalyst loading was increased to 2.5 mol% [Co] and the reaction time was extended to 110 h (all other conditions were identical to the optimized standard reaction conditions). The heterogeneous MOF-**P1**-Co catalyst gave rise to comparable ratios of remaining olefin to aldehyde product than were observed with the homogeneous analogue (Fig. S53 and S54): while MOF-**P1**-Co furnished an olefin : aldehyde ratio of 1 : 0.52, an olefin : aldehyde ratio of 1 : 0.58 was observed with the homogeneous catalyst. Despite the fact that the length of the cholesterol (1.9 nm) exceeds the diameter of the pore window of MIL-101 (1.6 nm), no significant mass transfer limitations were encountered.

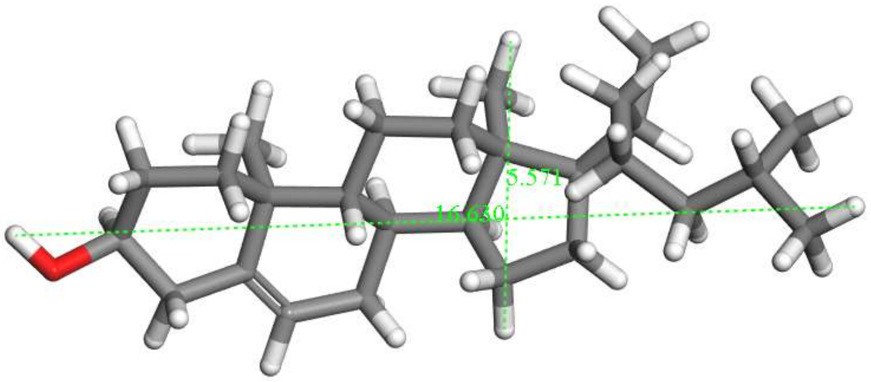

Figure S52 Estimated molecular dimensions of cholesterol based on a structure optimization in Materials Studio (DMol3, BLYP functional). The van der Waals diameter of hydrogen (240 pm) was added to arrive at the length and width values stated in the main text.

Due to the low efficiency of both the homogeneous and heterogeneous hydroformylation of cholesterol, we also prepared **S1** in which the alcohol moiety is protected with a TBDMS group to test whether the hydroxyl group interfered with cobalt-catalyzed hydroformylation. Since hydroformylation was less efficient with **S1** than with cholesterol, we concluded that the hydroxyl group is tolerated in the transformation and the low efficiency of hydroformylation for both the homogeneous and the heterogeneous catalyst is likely due to the steric hindrance surrounding the olefin.

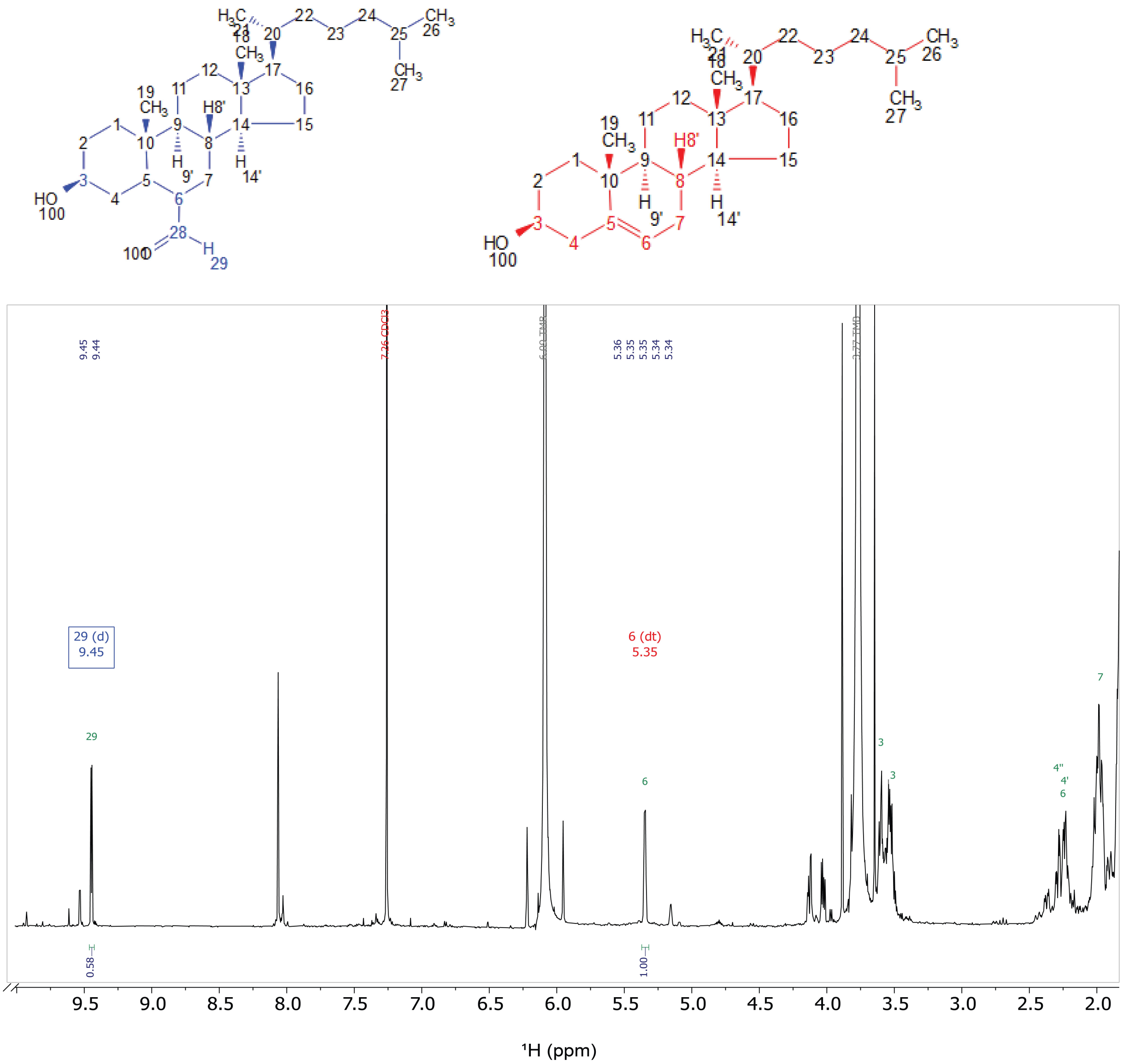

Figure S53. ^{1}H-NMR spectra of the unpurified reaction mixture obtained from homogeneous hydroformylation of cholesterol. Reaction condition: cholesterol (0.200 mmol, 77.3 mg), 2.5 mol% [Co], **P1** (0.009 mmol, 3.3 mg), 10 mL toluene, 110 h, 170 °C, 40 bar syngas.

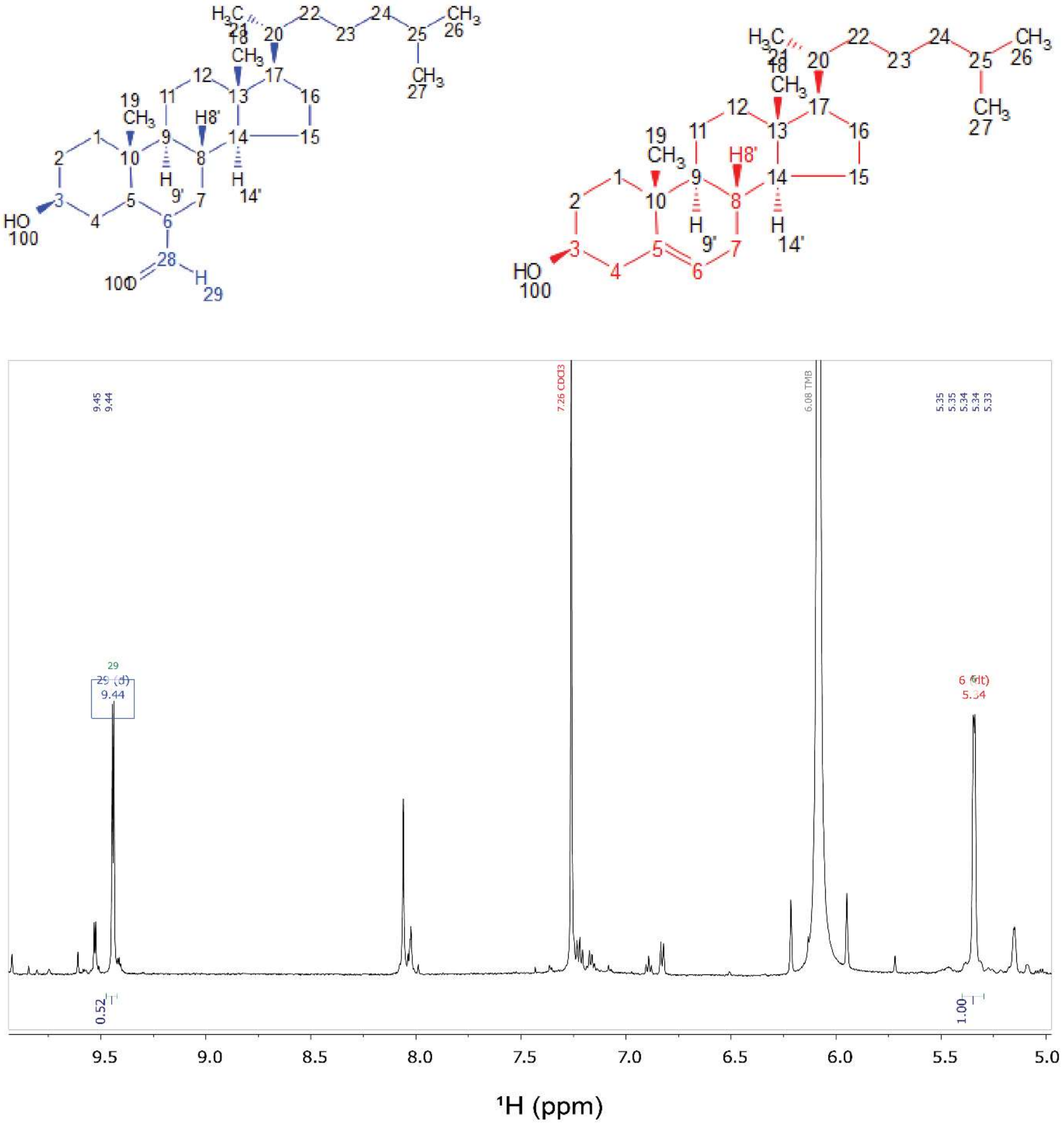

Figure S54 ^{1}H-NMR spectra of the unpurified reaction mixture obtained from heterogeneous hydroformylation of cholesterol. Reaction condition: cholesterol (0.200 mmol, 77.3 mg), 2.5 mol% [Co] in the form of MOF-**P1**-Co (23.7 mg), 10 mL toluene, 110 h, 170 °C, 40 bar syngas.

6.9 Hydroformylation of Resin-Bound Substrate

To rule out the possibility that the MOF-based catalyst functions through release of a catalytically active species into solution, olefin-functionalized Merrifield resin was investigated as a substrate for hydroformylation. Since the olefin substrate is attached to a bulky resin particle which is unable to enter the MOF pores, no conversion is expected for the heterogeneous hydroformylation of the olefin-functionalized Merrifield resin.

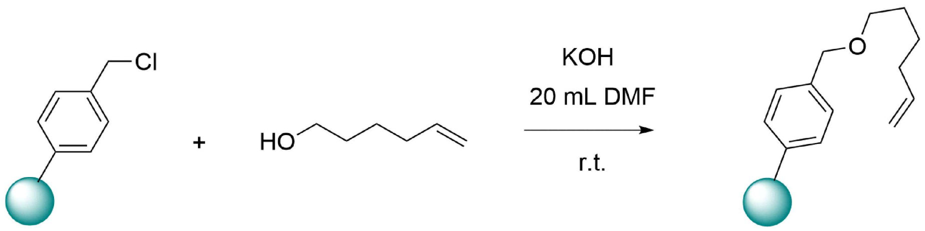

Olefin-functionalized Merrifield resin was prepared according to a reported procedure.[118] The successful attachment of the olefin moiety to the resin was confirmed by ATR-IR spectroscopy (Fig. S55).

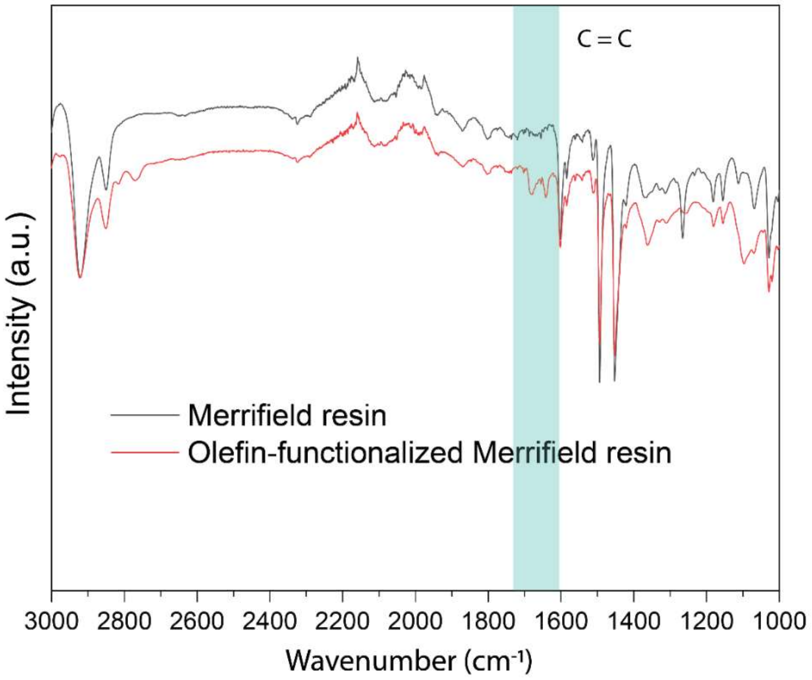

Figure S55. Comparison of ATR-IR spectra of Merrifield resin and olefin-functionalized Merrifield resin. The blue bar highlights the appearance of the C═C stretches around 1680 cm^{-1} after functionalization.

6.9.1 Procedure for Hydroformylation of Olefin-Functionalized Merrifield Resin

Two side-by-side reactions were performed, in which homogeneous and heterogeneous hydroformylation was carried out with an amount of catalyst that corresponds to 10.0 µmol cobalt in both cases.

Two autoclaves were prepared and 50.0 mg olefin-functionalized Merrifield resin was added into both. To one of the autoclaves, $Co_2(CO)_8$ (1.7 mg, 5.0 µmol), **P1** (3.3 mg, 9.0 µmol) and toluene (10 mL) were added. To the other autoclave, MOF-**P1**-Co (23.7 mg) and toluene (10 mL) were added. Both autoclaves were sealed, charged with 40 bar of syngas and heated at 170 °C. In-situ monitoring of the pressure change within the autoclave (Figure S57) showed that substantial conversion had occurred under homogeneous conditions. The extent of pressure change with the homogeneous catalyst for the resin-bound olefin was in good agreement with the real-time pressure variation trends observed during the optimization of heterogeneous hydroformylation with 1-hexene (Figure S56) when the reaction time was extended to 36 h. The reaction with the homogeneous catalyst was thus stopped after 36 h while the heterogeneous reaction, for which no pressure change could be observed, was monitored for an additional 12 h. To confirm the lack of reactivity under heterogeneous reaction condition, the heterogeneously catalyzed reaction was repeated, but no pressure change could be observed over the course of 55 h.

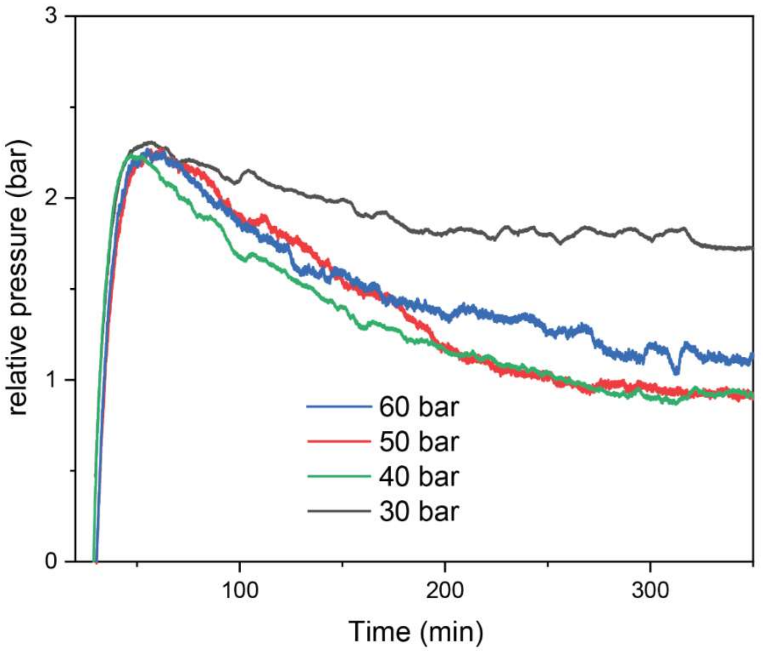

Figure S56 Comparison of reaction gas pressure profiles for the hydroformylation of 1-hexene with MOF-**P1**-Co with different pressures of syngas. All data was normalized to account for the different initial pressures. Reaction conditions: catalyst: MOF-**P1**-Co corresponding to 1 mol% Co; 0.96 mmol 1-hexene (1.00 equiv), 10 mL toluene, 6 h, 170 °C.

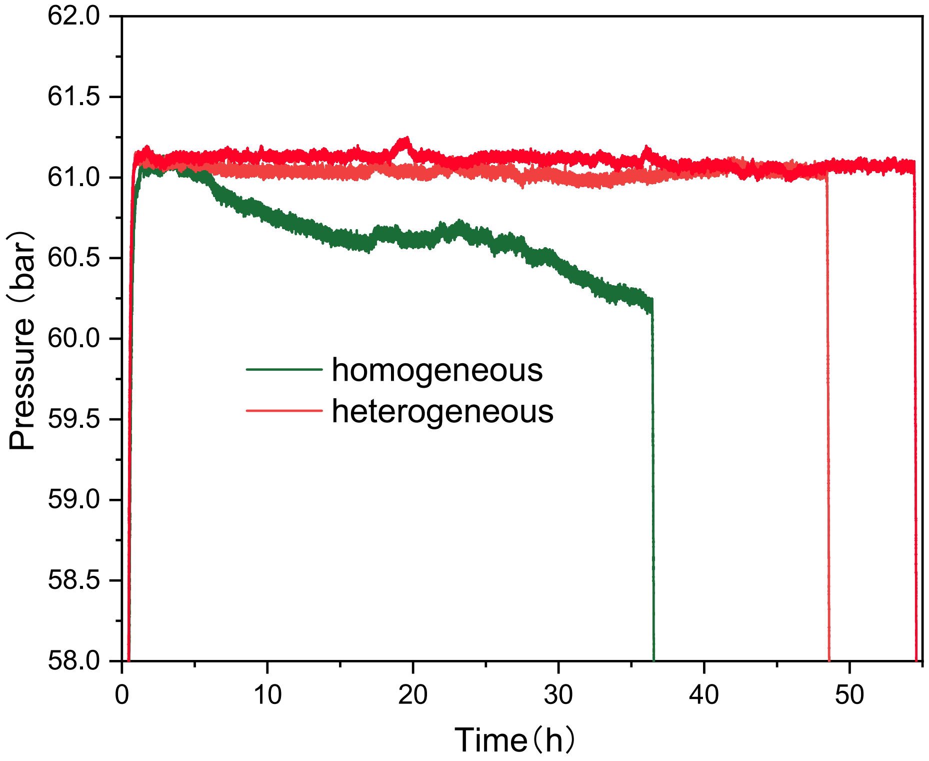

Figure S57. Comparison of reaction gas pressure profiles for the hydroformylation of olefin-functionalized Merrifield resin with homogenous ($Co_2(CO)_8$, **P1**) and heterogeneous (MOF-**P1**-Co) catalysts. The notable pressure drop observed with the homogeneous catalyst indicates that this catalyst mediates gas-consuming processes (hydrogenation or hydroformylation), while the heterogeneous counterpart does not promote any gas-consuming reactions.

7 Recycling Experiments

7.1 Experimental Procedure

In an argon-filled glovebox, an autoclave containing a magnetic stir bar was filled with 11.8 mg MOF-**P1**-Co, 10 mL toluene, and 0.96 mmol 1-hexene, before it was sealed inside glovebox. The autoclave was pressurized with CO (20 bar) and 20 bar H_2 (20 bar), and then heated at 170 ºC for 6 hours under continuous stirring (250 rpm). After the reaction was completed and the autoclave cooled down, the exhaust valve was connected to a tube, with the other end of the tube placed under the water before the gas pressure within the autoclave was released slowly. The release valve was closed again and the autoclave was transferred into a glovebox. Inside the glovebox, the liquid inside the autoclave was collected in a vial, which was closed, removed from the glovebox and subjected to centrifugation. The vial was brought back into the glovebox where the solid catalyst was collected and washed twice with toluene. Meanwhile, the supernatant liquid was analyzed by GC to determine the product distribution obtained in the hydroformylation reaction. The washed catalyst was placed inside a clean autoclave containing a stir bar and the reagents for the next reaction cycle were added.

Parallel experiments were conducted to ensure that material was available for characterization after each cycle, while another batch could be used to carry out a subsequent reaction cycle.

Procedure for ICP-OES measurement: After each cycle, the suspension was centrifuged to recover the solid catalyst which was used for the next reaction cycle while the supernatant was filtered by 0.22 µm PTFE filter. Around 3 g liquid was weighed and the exact weight was recorded. A rotary evaporator was then used to remove volatile organic components. The remaining solid, if present, was dissolved in deionized water and acid. The resulting solution was analyzed by ICP-OES to determine the metal and phosphorus content.

Hot filtration: After the catalyst had been subjected to the THF washing procedure, it was washed three times with toluene and then added to an autoclave that contained 10 mL toluene and 0.96 mmol 1-hexene. The autoclave was sealed inside glovebox and then removed from the glovebox. The autoclave was pressurized with CO (20 bar) and 20 bar H_2 (20 bar), and then heated at 170 ºC for 1 hour or 2 hours under continuous stirring (250 rpm). After the chosen time had elapsed, the autoclave was cooled down, the gas released, and the autoclave opened inside a glovebox. The liquid inside the autoclave was then collected into a vial, which was closed, removed from the glovebox and subjected to centrifugation (9000 rmp). The suspension was filtered by using a 0.22 µm PTFE filter before it was added back into an autoclave and subjected to the reaction conditions for hydroformylation for an additional 5 hours (1 h initial reaction time) or 4 hours (2 h initial reaction time).

7.2 Recycling of MOF-P1-Co that was not washed with THF

The main text presented recycling data for MOF-**P1**-Co that was washed with THF at the end of the synthesis (see experimental procedures for a description of the workflow). This washing step was introduced after our initial recycling experiment (Fig. S58) with MOF-**P1**-Co that had only been washed with THF at room temperature at the end of the synthesis. While satisfactory catalyst activity and selectivity was observed in the first reaction cycles, we determined a non-negligible amount of cobalt leaching in addition to a slight decrease in the conversions observed in cycle 4 and cycle 5. The THF washing procedure was thus introduced, which removes traces of loosely bound cobalt (3.12 ppm Co

detected in recovered liquid) in addition to changing the speciation of the cobalt centers (see Fig. 6A and discussion in the main text). Repeated recycling of MOF-**P1**-Co that had been subjected to a THF wash resulting in less than <0.7 ppm cobalt leaching and <0.05 ppm phosphine leaching (Table S11). Due to the very small particle size of MIL-101 and the small reaction volumes, full removal of the particles via centrifugation was challenging so that the residual amounts of chromium in solution may indicate very limited MOF degradation or simply imperfect removal of the crystallites from the solution subjected to ICP-OES analysis. Notably, no signs of degradation of the MOF crystallites due to partial decomposition of the MOF was apparent from the electron microscopy data of the recovered catalyst.

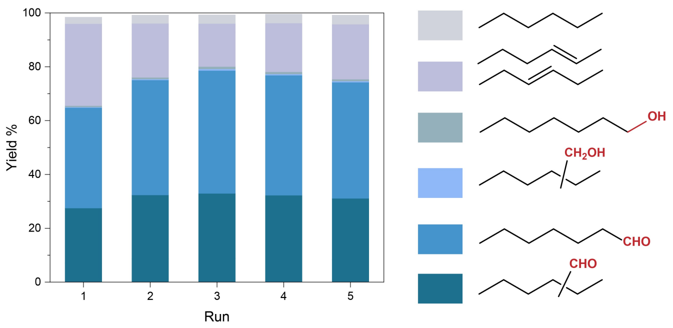

Figure S58. Recycling experiment. Reaction conditions: 11.8 mg MOF-**P1**-Co (not subjected to THF wash after synthesis) which corresponds to 0.5 mol% Co; 0.96 mmol hexene, 10.0 mL toluene, 6 h, 170 °C, 40 bar syngas.

7.3 Recycling of THF-Washed Catalyst

Table S11. Concentrations of chromium, cobalt and phosphorus detected in the filtered reaction mixture as determined by ICP-OES analysis.

**run**	**Cr (ppm)**	**Co (ppm)**	**P (ppm)**
catalyst washing with THF	1.48	3.12	0.08
cycle 1	7.89	0.62	0.04
cycle 2	8.77	0.57	0.02
cycle 3	9.48	0.29	0.04
cycle 4	20.96	0.52	0.03

7.4 Hot filtration

To test whether any leaching of catalytically active species from MOF-**P1**-Co takes place over the course of the reaction, two hot filtration experiments were carried out (Fig. S59). The amounts of aldehyde and alcohol that were detected after the filtered liquid was resubmitted to the reaction conditions after hot filtration after either 1 h or 2 h did not differ significantly from the values obtained after a reaction time of 1 h or 2 h, respectively. The differences in reaction yields observed did not exceed 2% and are thus identical to the experimental error observed for repeated experiments during kinetic experiments (c.f. Table S9).

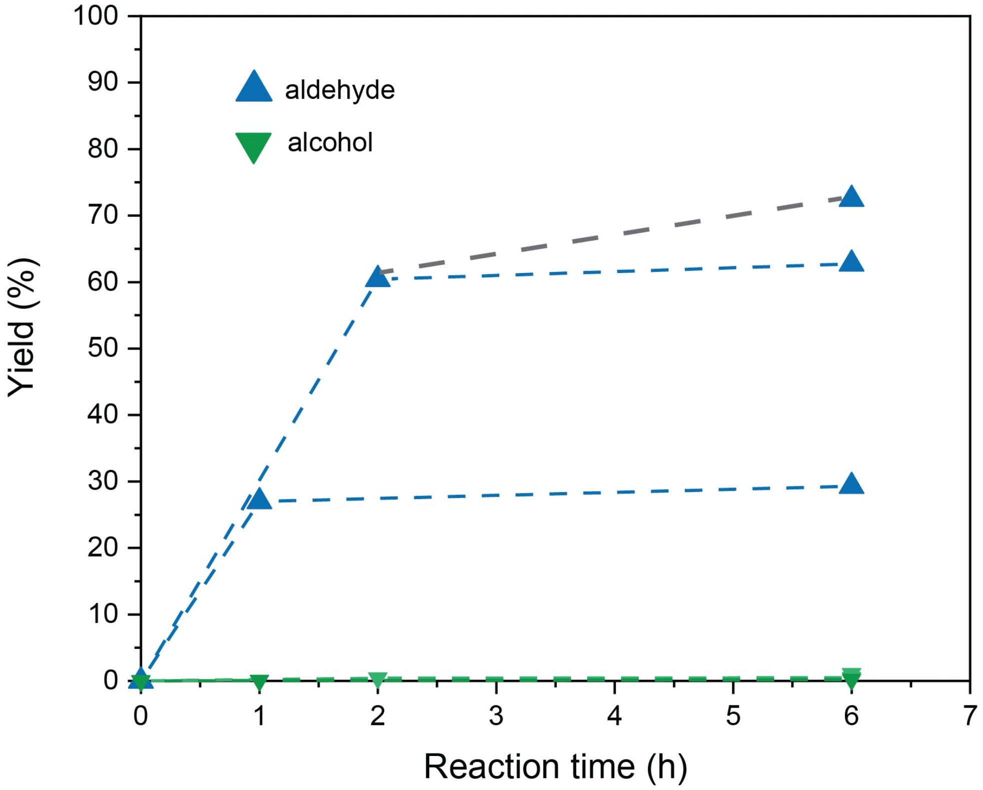

Figure S59. Comparison of catalytic activity with MOF-**P1**-Co under standard condition (grey line) and after the hot filtration of catalysts at 1 hour or 2 hour (blue line). Reaction condition: 11.8 mg MOF-**P1**-Co which corresponds to 0.5 mol% Co; 0.96 mmol hexene, 10.0 mL toluene, 170 °C, 40 bar syngas.

8 Comparison of Pristine and Recovered Catalyst

8.1 Electron Microscopy

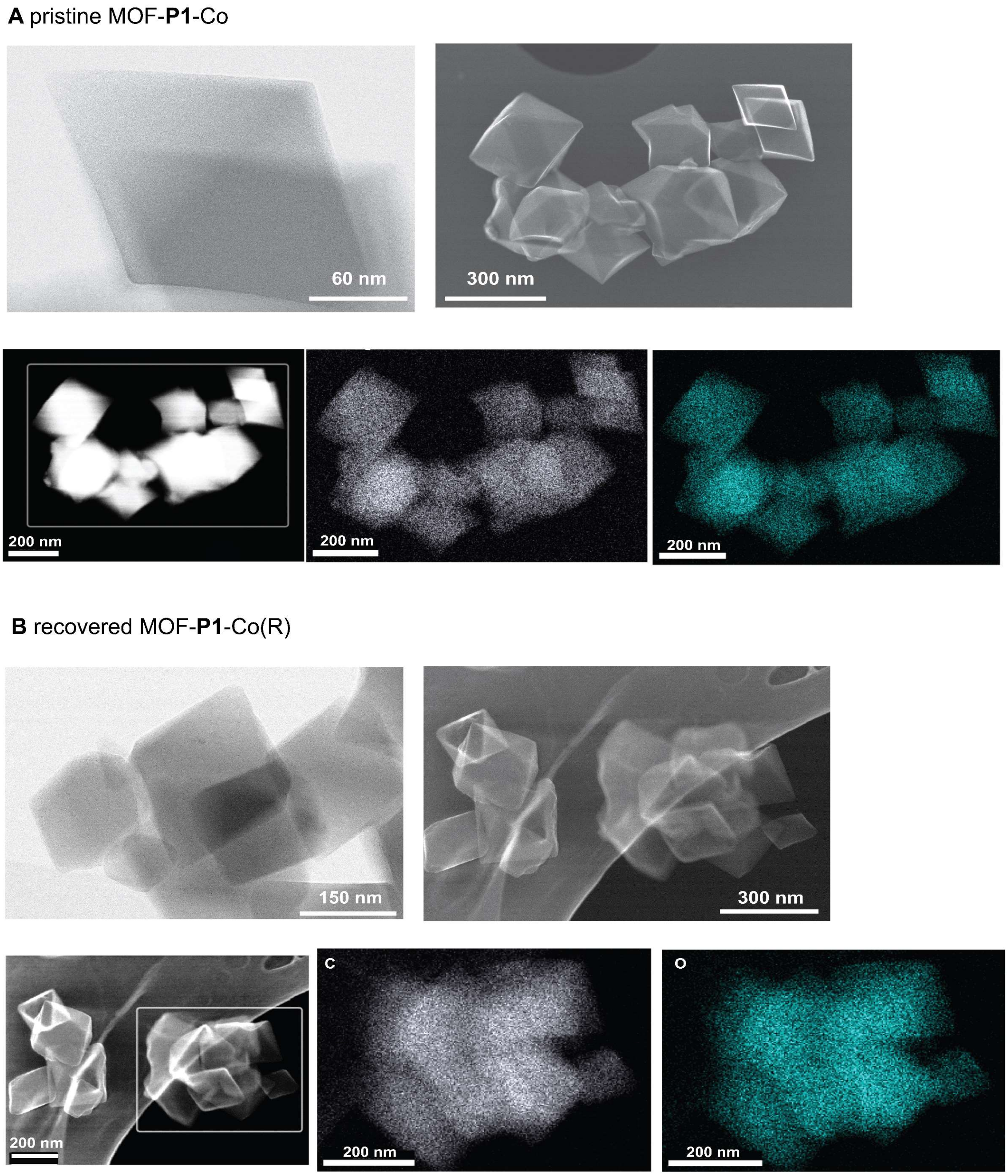

Figure S60. TEM images and EDX maps for C and O (EDX maps for other elements are shown in main text) for pristine (MOF-**P1**-Co) and recovered (MOF-**P1**-Co(R)) catalyst.

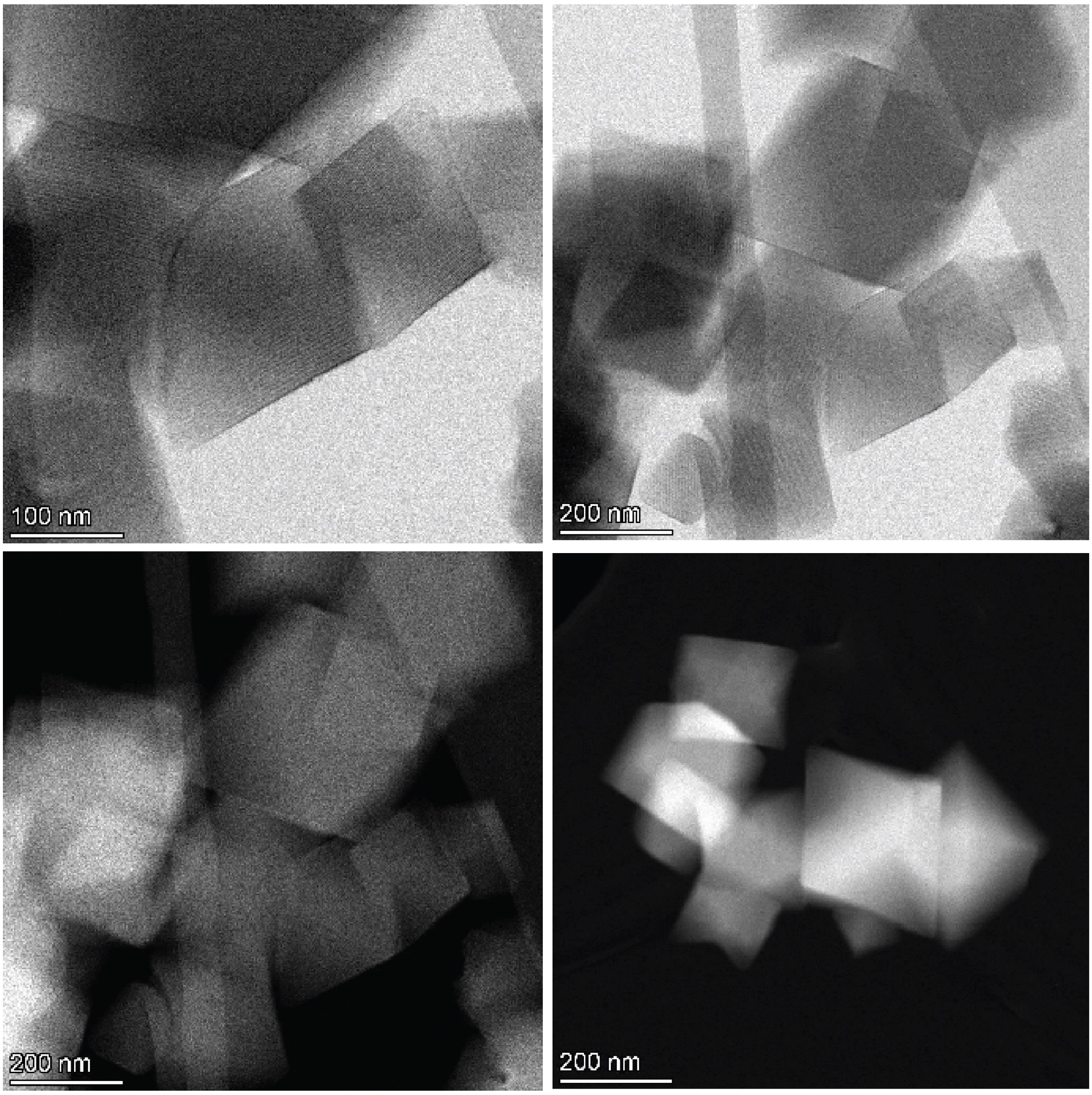

Figure S61. STEM images of MOF-**P2**-Co.

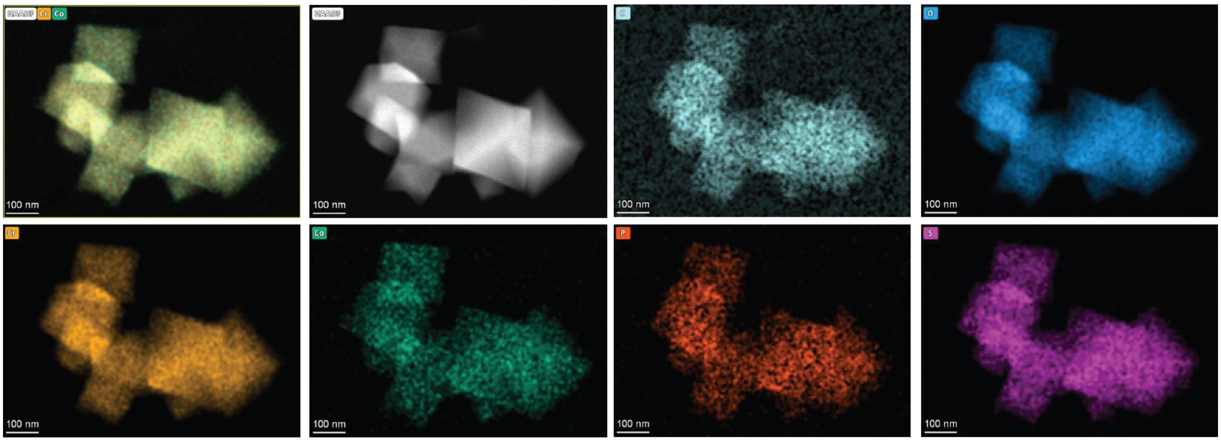

Figure S62. EDX maps for pristine MOF-**P2**-Co.

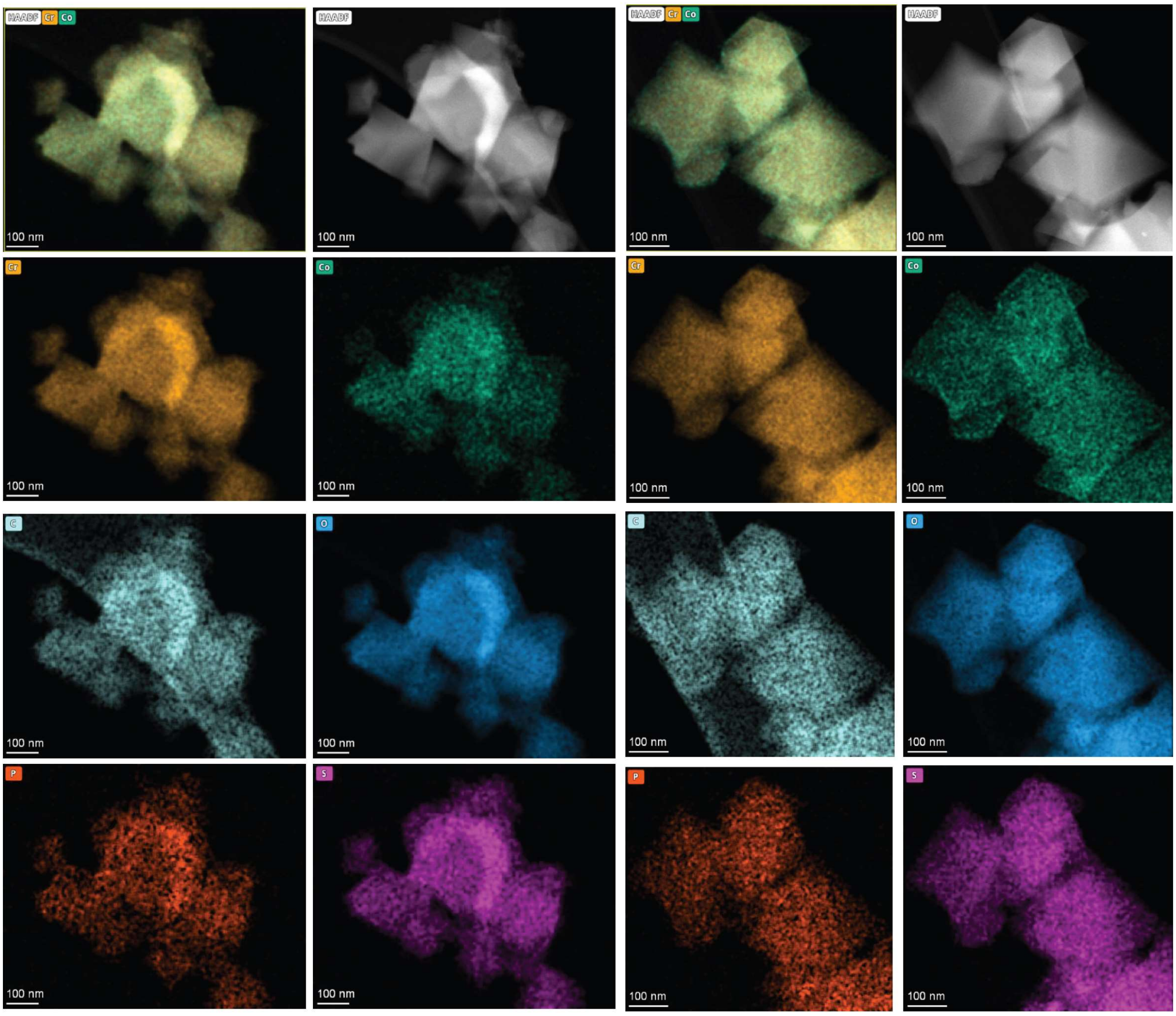

Figure S63. EDX maps for recovered catalyst MOF-**P2**-Co(R).

8.2 Nuclear Magnetic Resonance

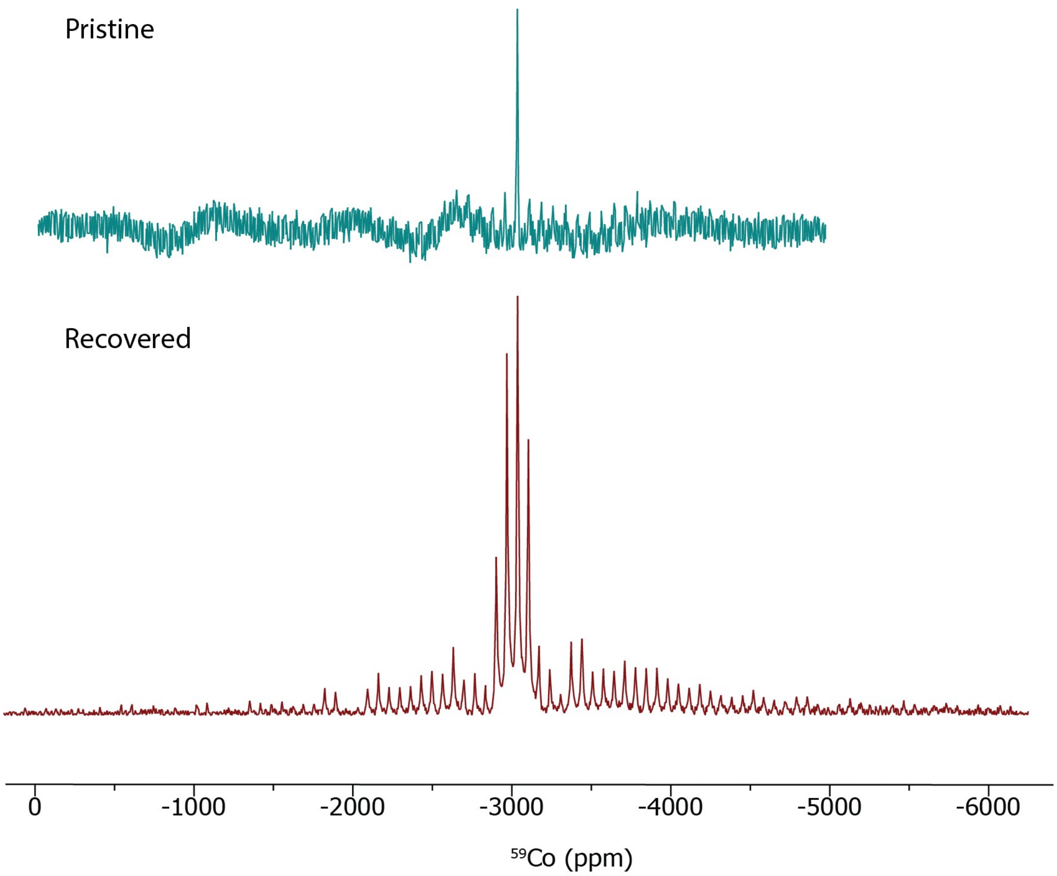

Figure S64. Comparison of solid-state ^{59}Co NMR spectra of pristine MOF-**P1**-Co and recovered MOF-**P1**-Co(R). The recovered sample was subjected to NMR analysis after 2 reaction cycles. The spectrum of the pristine catalyst was measured in a 4 mm rotor and the spectrum of the recovered catalyst was measured in a 7 mm rotor. The number of NMR scans carried out were 16384 (pristine catalyst) and 49540 (recovered catalyst).

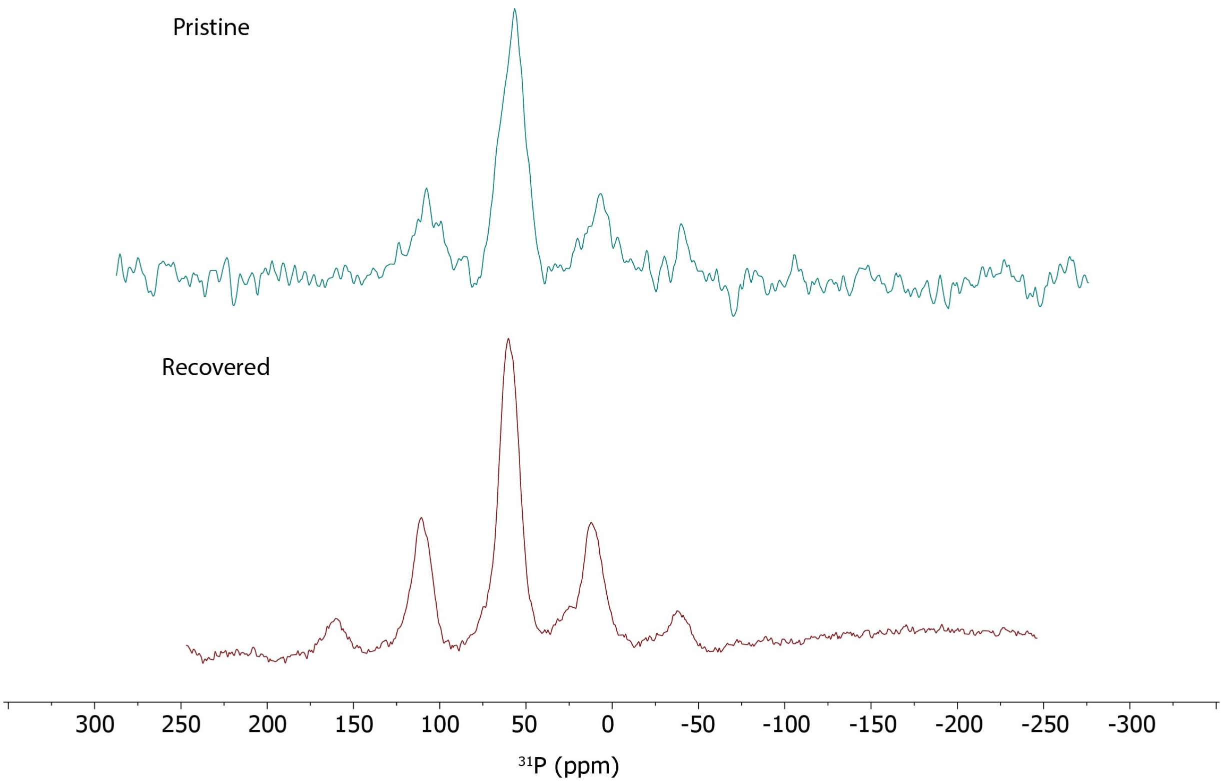

Figure S65. Comparison of solid-state ^{31}P NMR spectra of pristine MOF-**P1**-Co and recovered MOF-**P1**-Co(R). The spectrum of the recovered sample was collected after 2 reaction cycles. The number of NMR scans collected was 2084 (pristine catalyst) and 12288 (recovered catalyst).

8.3 X-ray Photoelectron Spectroscopy

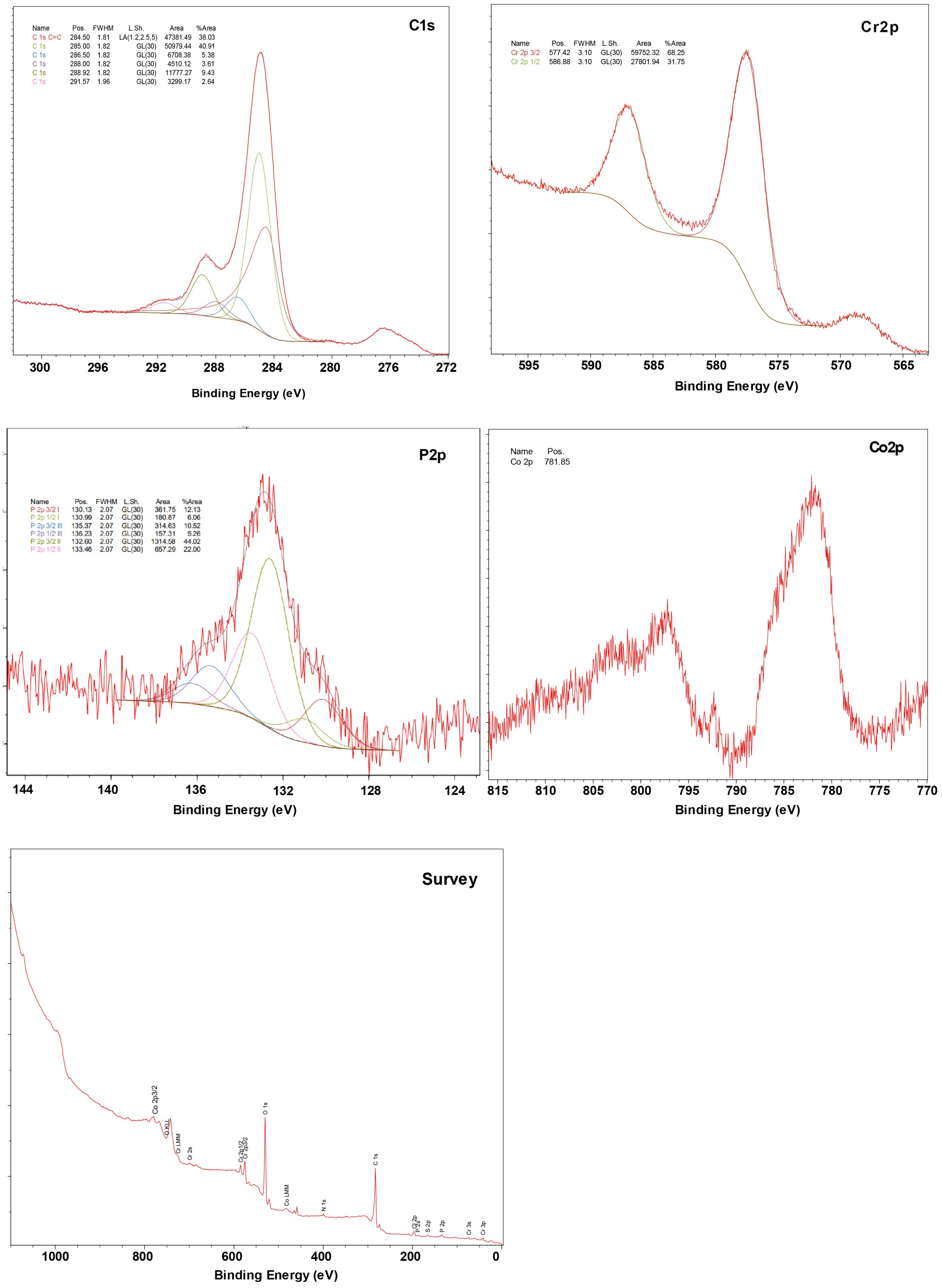

Figure S66. Survey and high-resolution XP spectra of MOF-**P1**-Co.

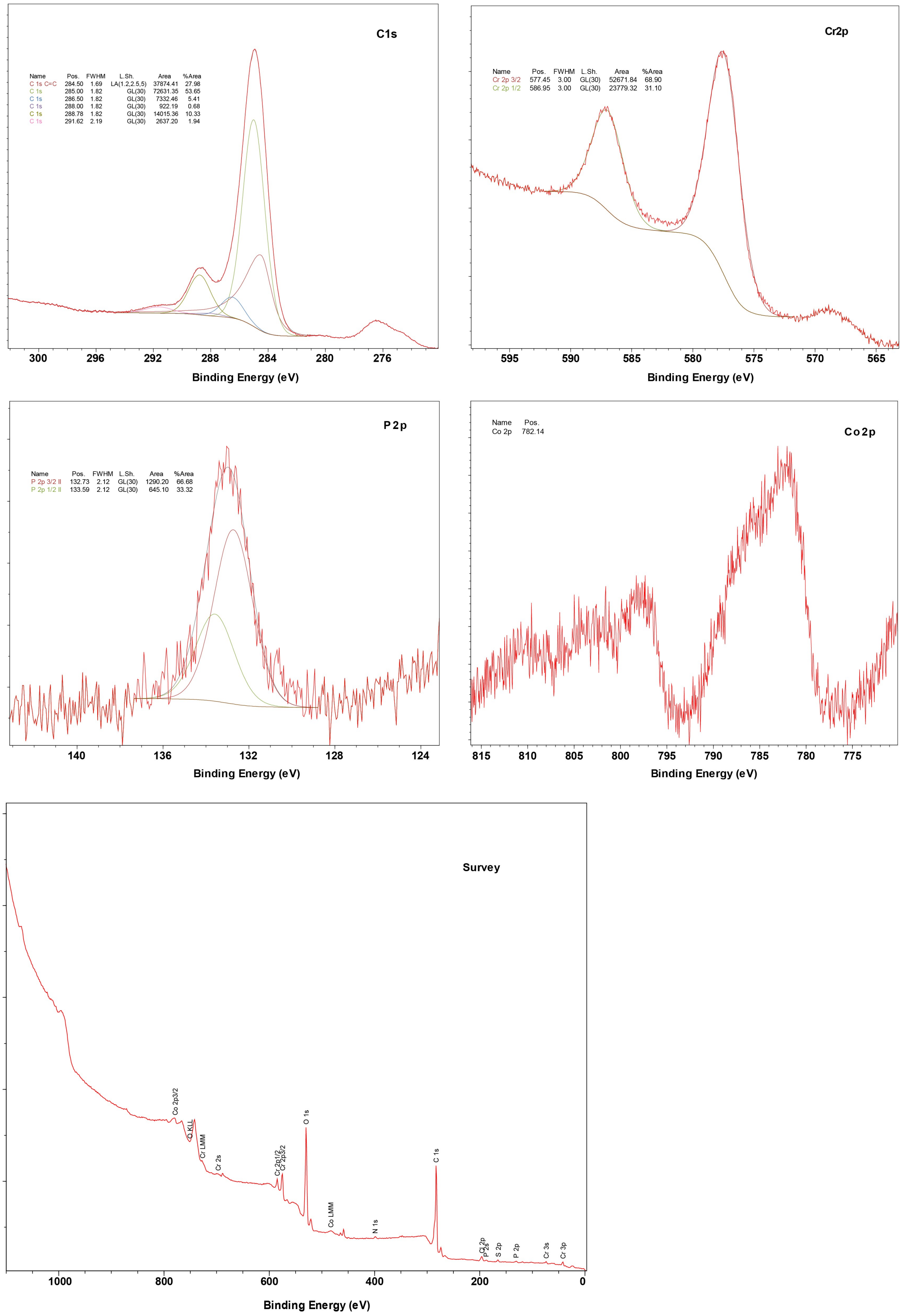

Figure S67. Survey and high-resolution XP spectra of MOF-**P1**-Co(R).

9 Structure of the Catalyst Resting State

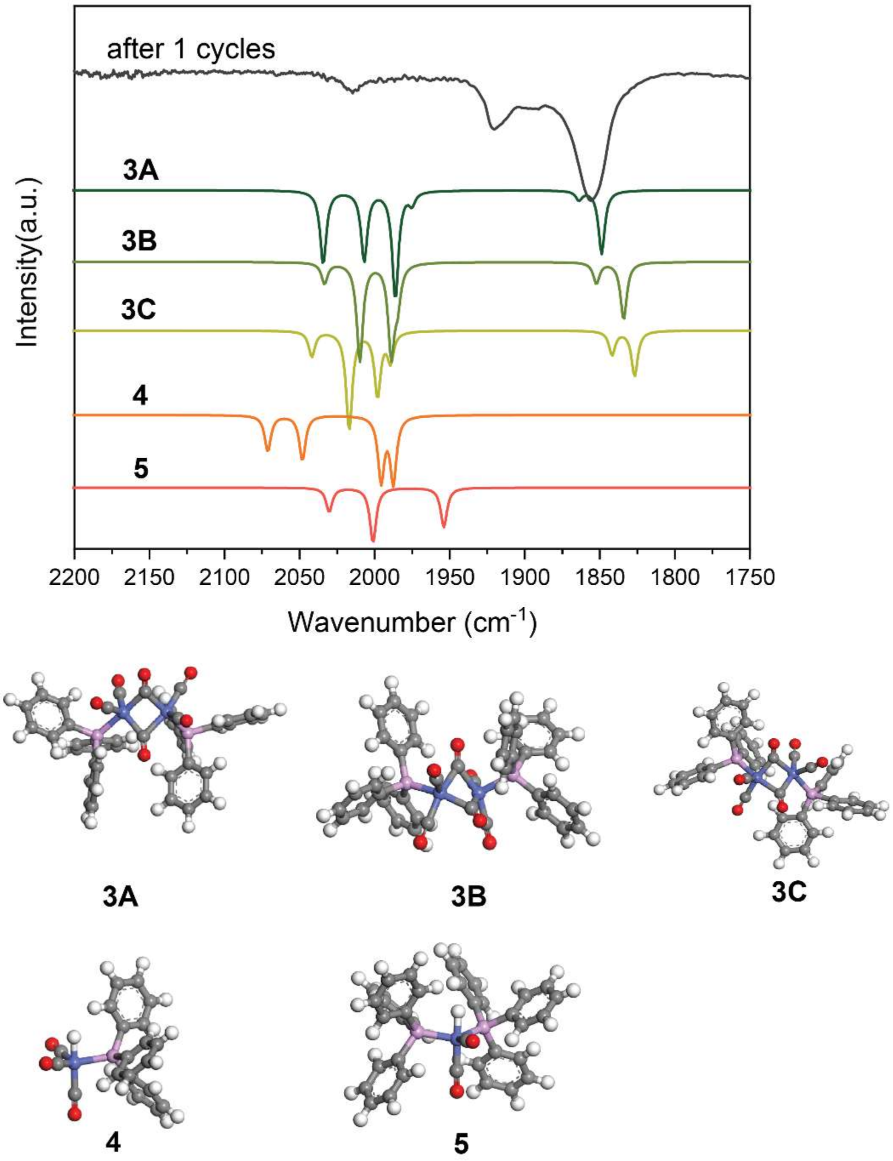

Figure S68. Comparison of the experimental AT-IR spectrum of MOF-**P1**-Co(R) with the theoretically predicted IR spectra of a range of different cobalt-containing candidate structures.

10 Recovery of MIL-101 from MOF-P1

10.1 Unsuccessful Phosphine Removal with Excess Salt

Removal of **P1** from the MOF framework in the presence of excess salt and a polar organic solvent proved unsuccessful (Fig. S69A), which highlights that **P1** is securely heterogenized within the MOF and the presence of even high concentrations of ionic species did not give rise to undesired leaching.

Experimental Procedure:

50 mg MOF-**P1** and NaCl (58.4 mg, 1.00 mmol, 50 equiv.) or LiCl (20.5 mg, 1.00 mmol, 50 equiv.) and MeOH (100 mL) were added to a 250 mL flask containing a stir bar. The reaction mixture was heated at 80 ^oC with stirring (500 rmp). After 16 h, the resulting green suspension was transferred to a 50 mL centrifuge tube, sealed, and subjected to centrifugation (4500 rpm for 15 min). No phosphine could be detected in the supernatant by either TLC or NMR analysis.

10.2 Successful Phosphine Removal with Excess Salt and Water

In the presence of both excess salt and a mixture of methanol and water the phosphine ligand could effectively be removed from the MOF pore (Fig. S69A) and MIL-101 was recovered from MOF-**P1** with undiminished quality (Fig. S69C).

Experimental Procedure:

50 mg MOF-**P1**, NaCl (58.4 mg, 1.00 mmol, 50 equiv.), methanol (50 mL) and water (50 mL) were added to a 250 mL flask containing a stir bar. The reaction mixture was heated at 80 ^oC with stirring (500 rmp). After 16 h, the resulting green suspension was transferred to a 50 mL centrifuge tube, sealed, and subjected to centrifugation (4500 rpm for 15 min). The supernatant was collected and analyzed by NMR spectroscopy and TLC (Figure S69B) before the solid was washed three times with water. The entire procedure was repeated two more times to ensure full phosphine removal. A further repetition was also attempted but no further removal of phosphine ligand could be detected so three repetitions were chosen as the optimized removal protocol.

The recovered MIL-101 was dried and analyzed by Ar sorption, PXRD, and ICP-OES (see main text and Fig. S69C).

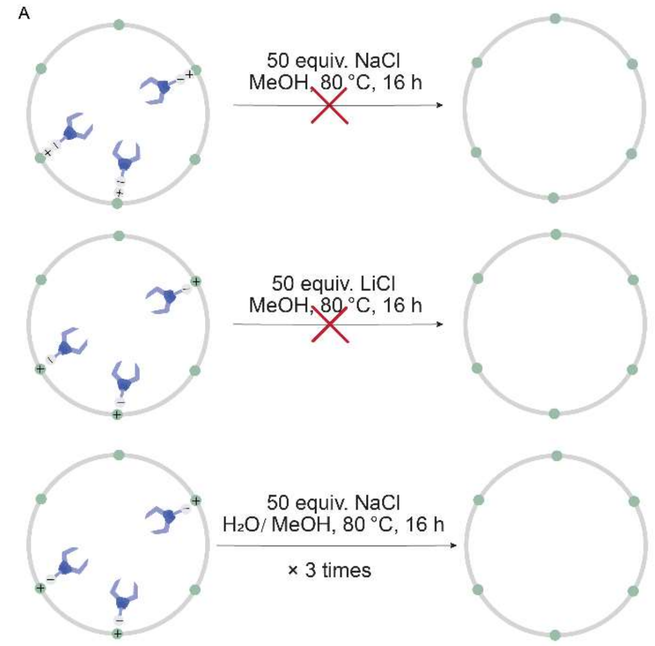

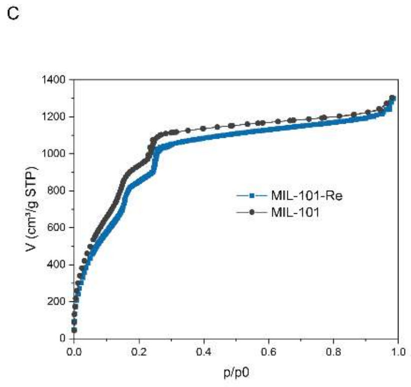

	Surface area ($m^2 \cdot g^{-1}$)	Porevolume ($cm^3 \cdot g^{-1}$)
MIL-101	2895	1.66
MIL-101-Re	2556	1.65

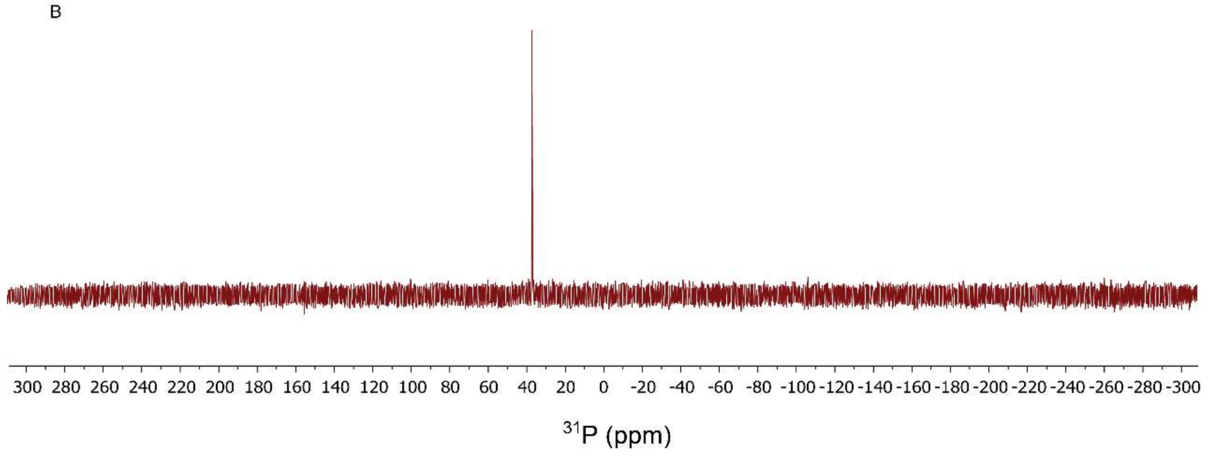

Figure S69 A Attempted removal of phosphine ligand and recovery of MOF support under different reaction conditions. Only in the presence of excess salt and water at elevated temperature could phosphine removal be detected. **B** ^{31}P NMR of supernatant. A chemical shift of 36 ppm indicated that the phosphine ligand was oxidized to the phosphine oxide during ligand removal, which was carried out under air. **C** Comparison of Ar adsorption isotherms, surface areas and pore volumes of pristine and recovered MIL-101 samples.

11 Computational Details

All computational analyses were carried out in the DMol³ module of the BIOVIA Materials Studio 2020 software package.[119-120]

In order to select a DFT functional that furnishes a high degree of agreement between predicted and experimentally observed stretching frequencies, a number of different functionals were compared (Table S12) for a relevant model system ($[Co(CO)_4]^-$ in the gas phase). The computationally predicted stretching frequencies for the CO ligand and the predicted C–O bond lengths were compared with experimental values. The PW91 functional was able to predict stretching frequencies (1883, 1885, and 1887 cm^{-1}) and C–O bond lengths (1.769 Å) that best reproduced experimental data (1894 cm^{-1} and 1.752 Å) and was thus chosen for all further analyses.

Table S12. Selection of the optimal functional for the model system ($[Co(CO)_4]^-$ in the gas phase).

Functional	CO stretch 1 [cm^{-1}]	CO stretch 2 [cm^{-1}]	CO stretch 3 [cm^{-1}]	Bond length [Å]
**PW91**	**1883**	**1885**	**1887**	**1.769**
BP	1878	1879	1881	1.770
BOP	1843	1843	1843	1.794
VWN-BP	1879	1880	1882	1.769
RPBEP	1879	1881	1883	1.781
HCTH	2047	2049	2051	1.764
PBEsol	1923	1923	1923	1.751
BLYP	1855	1858	1860	1.793
PBE	1947	1969	1990	1.766
SCAN	1961	2015	2030	1.741

Theoretical structures for a range of observed and proposed cobalt-containing structures were optimized in the gas phase with the PW91 functional using a dnd basis set and a fine integration cut-off. Vibrational frequency calculations were carried out for all structures to confirm the absence of imaginary frequencies for ground state structures. The predicted CO stretching frequencies were compared to experimental results throughout the work in order to aid in the assignment of the prepared structures.

11.1 List of DFT-Optimized Atomic Coordinates

$[Co(CO)_3(PPh_3)_2]^+$

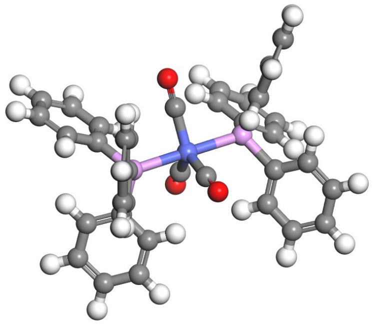

Charge: +1

	ATOM	X	Y	Z
1	Co	0.016955	-0.006364	-0.011847
2	P	1.280791	1.380665	1.276802
3	P	-1.259013	-1.399933	-1.278174
4	O	-1.516363	2.305239	-1.032518
5	O	-0.781142	-1.505959	2.398640
6	O	2.404515	-0.788370	-1.558341
7	C	-0.922856	1.402471	-0.621110
8	C	-0.467131	-0.916423	1.453498
9	C	1.469131	-0.480908	-0.953017
10	C	-0.468067	-3.013059	-1.632017
11	C	0.318489	-3.642824	-0.653467
12	H	0.505542	-3.155262	0.302286
13	C	0.871891	-4.899468	-0.898893
14	H	1.480027	-5.380442	-0.132997
15	C	0.651667	-5.537885	-2.121979
16	H	1.090997	-6.516891	-2.313357
17	C	-0.127910	-4.916480	-3.099420
18	H	-0.302232	-5.408147	-4.055545
19	C	-0.688048	-3.660458	-2.860192
20	H	-1.292848	-3.185223	-3.631026
21	C	-1.650813	-0.678207	-2.913795

22	C	-0.622404	-0.487211	-3.855165
23	H	0.396060	-0.810107	-3.639942
24	C	-0.903465	0.104009	-5.086160
25	H	-0.103457	0.239282	-5.813873
26	C	-2.203751	0.521405	-5.387177
27	H	-2.418710	0.987615	-6.348239
28	C	-3.224656	0.339467	-4.454238
29	H	-4.240843	0.658015	-4.683268
30	C	-2.954277	-0.260764	-3.222186
31	H	-3.760879	-0.403471	-2.504586
32	C	-2.875040	-1.802362	-0.511576
33	C	-3.487642	-3.038829	-0.774744
34	H	-2.989460	-3.779243	-1.398607
35	C	-4.737023	-3.331273	-0.226996
36	H	-5.200562	-4.295265	-0.433384
37	C	-5.385168	-2.399830	0.587079
38	H	-6.357843	-2.635099	1.018319
39	C	-4.777939	-1.171714	0.857437
40	H	-5.275416	-0.443419	1.497600
41	C	-3.527761	-0.872384	0.313531
42	H	-3.058914	0.083781	0.543168
43	C	0.304613	2.747168	2.003125
44	C	-0.926544	2.462407	2.619322
45	H	-1.309459	1.442775	2.643837
46	C	-1.666863	3.485133	3.210181
47	H	-2.616854	3.255594	3.691675
48	C	-1.192532	4.800005	3.184599
49	H	-1.775920	5.599183	3.640666
50	C	0.027586	5.087975	2.571978
51	H	0.402621	6.110331	2.550243
52	C	0.779909	4.067161	1.985301
53	H	1.733056	4.301273	1.514124
54	C	2.065895	0.538263	2.699243
55	C	2.103045	1.156527	3.958748

56	H	1.639239	2.130288	4.107687
57	C	2.733851	0.520618	5.030074
58	H	2.752413	1.004966	6.005708
59	C	3.332753	-0.727550	4.854080
60	H	3.821407	-1.221556	5.693367
61	C	3.299282	-1.346740	3.601386
62	H	3.765923	-2.320827	3.458705
63	C	2.665046	-0.721695	2.528079
64	H	2.639534	-1.216837	1.557252
65	C	2.652841	2.169661	0.356666
66	C	3.974142	2.084742	0.823057
67	H	4.199540	1.548922	1.743366
68	C	5.009341	2.689294	0.105452
69	H	6.031843	2.614788	0.473883
70	C	4.735693	3.383924	-1.073111
71	H	5.545391	3.853675	-1.630643
72	C	3.420259	3.475598	-1.539211
73	H	3.201556	4.018654	-2.457864
74	C	2.382627	2.865783	-0.835765
75	H	1.364336	2.941820	-1.215733

$[Co(CO)_4]^-$

Charge: –1

ATOM		X	Y	Z
1	Co	0.000000	0.000000	0.019917

2	O	2.382554	0.000000	-1.713708
3	O	0.000000	-2.430700	1.686631
4	O	-2.382554	0.000000	-1.713708
5	O	0.000000	2.430700	1.686631
6	C	1.436381	0.000000	-1.013222
7	C	0.000000	-1.453015	1.030341
8	C	-1.436381	0.000000	-1.013222
9	C	0.000000	1.453015	1.030341

Complex **2**

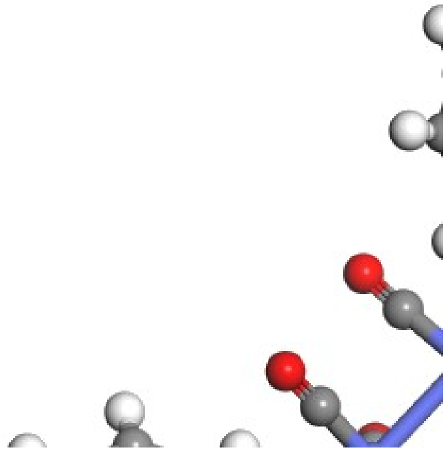

Charge: 0

	ATOM	X	Y	Z
1	Co	0.977786	0.942132	-0.061610
2	P	2.619291	2.397807	-0.163549
3	O	0.580553	1.224543	2.850591
4	O	2.652456	-1.256483	-1.096578
5	O	-0.760592	2.477719	-1.890910
6	C	0.730458	1.103993	1.702803
7	C	1.974046	-0.401520	-0.690657
8	C	-0.090772	1.861598	-1.165643
9	C	2.084394	4.158497	-0.109657
10	C	3.845362	2.276383	1.205486
11	C	3.653234	2.302585	-1.686395

12	Co	-1.012340	-0.889796	0.009817
13	P	-2.618538	-2.382002	0.149254
14	O	-0.595548	-1.199186	-2.897089
15	O	-2.744992	1.309883	0.944731
16	O	0.727675	-2.334487	1.908167
17	C	-0.750756	-1.073989	-1.750369
18	C	-2.046656	0.455319	0.574751
19	C	0.054951	-1.751518	1.157485
20	C	-2.311675	-3.950858	-0.768678
21	C	-4.248204	-1.825759	-0.504827
22	C	-2.997205	-2.949916	1.863348
23	C	4.239622	1.003051	1.647107
24	C	5.174789	0.870018	2.673323
25	C	5.715808	2.005682	3.281588
26	C	5.323622	3.275215	2.852760
27	C	4.392509	3.413437	1.820007
28	C	3.021500	2.071703	-2.919306
29	C	3.768015	2.023405	-4.096882
30	C	5.154994	2.188839	-4.057656
31	C	5.791911	2.404501	-2.834402
32	C	5.047353	2.463240	-1.653030
33	C	1.095608	4.533883	0.814622
34	C	0.668041	5.859309	0.889438
35	C	1.208297	6.821100	0.031691
36	C	2.183682	6.453174	-0.896496
37	C	2.621924	5.127979	-0.969014
38	C	-2.984294	-2.013486	2.910052
39	C	-3.282864	-2.408082	4.214710
40	C	-3.578120	-3.744727	4.494818
41	C	-3.581054	-4.684094	3.462070
42	C	-3.293395	-4.291484	2.152471
43	C	-1.013783	-4.485577	-0.778311
44	C	-0.753628	-5.693794	-1.425906
45	C	-1.781746	-6.371350	-2.086092

46	C	-3.073066	-5.840124	-2.091465
47	C	-3.340232	-4.634658	-1.436935
48	C	-4.283780	-1.172167	-1.747955
49	C	-5.498804	-0.759397	-2.293243
50	C	-6.691105	-0.978116	-1.597817
51	C	-6.662413	-1.615441	-0.356450
52	C	-5.447493	-2.038014	0.191019
53	H	3.803396	0.112462	1.196874
54	H	5.469733	-0.125087	3.005736
55	H	6.435633	1.900432	4.093319
56	H	5.739089	4.165664	3.324739
57	H	4.087820	4.408407	1.497267
58	H	1.943641	1.919328	-2.958779
59	H	3.262864	1.846473	-5.046044
60	H	5.738335	2.138840	-4.976877
61	H	6.874701	2.524720	-2.793031
62	H	5.555686	2.629247	-0.704111
63	H	0.650121	3.787370	1.471080
64	H	-0.098918	6.136192	1.612613
65	H	0.864527	7.853837	0.082278
66	H	2.606547	7.195736	-1.573352
67	H	3.379355	4.849319	-1.700504
68	H	-2.728267	-0.974151	2.709998
69	H	-3.270986	-1.669597	5.016473
70	H	-3.794956	-4.054070	5.517400
71	H	-3.802756	-5.730241	3.673757
72	H	-3.295300	-5.033844	1.355721
73	H	-0.202054	-3.952694	-0.285836
74	H	0.257223	-6.100879	-1.419630
75	H	-1.576971	-7.310079	-2.600060
76	H	-3.879790	-6.362823	-2.605465
77	H	-4.349807	-4.225283	-1.449321
78	H	-3.359296	-0.983375	-2.291307
79	H	-5.510664	-0.259623	-3.261828

80	H	-7.639387	-0.647393	-2.021244
81	H	-7.588447	-1.785544	0.193011
82	H	-5.435896	-2.532317	1.161939

Complex **3A**

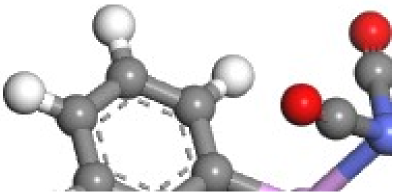

Charge: 0

ATOM		X	Y	Z
1	P	-2.693886	-0.056514	-0.602340
2	C	-3.068364	1.324286	0.561571
3	C	-4.329007	-0.359470	-1.414565
4	C	-2.545803	-1.514288	0.512224
5	C	-1.269355	-1.919140	0.923770
6	C	-1.117028	-2.980748	1.817851
7	C	-2.241134	-3.656994	2.297047
8	C	-3.517416	-3.272357	1.876373
9	C	-3.671935	-2.203569	0.991567
10	C	-3.130711	2.637340	0.066309
11	C	-3.448116	3.698649	0.913950
12	C	-3.691072	3.466824	2.270570
13	C	-3.615915	2.166891	2.774260
14	C	-3.305852	1.100890	1.925843
15	C	-4.455029	-1.445210	-2.298783
16	C	-5.669693	-1.704818	-2.933715
17	C	-6.772637	-0.875958	-2.708654
18	C	-6.656057	0.204504	-1.833740
19	C	-5.443447	0.460461	-1.186284

20	H	-0.391004	-1.414584	0.523609
21	H	-0.116420	-3.283798	2.123621
22	H	-2.121425	-4.492025	2.988399
23	H	-4.397098	-3.805283	2.238319
24	H	-4.669844	-1.909835	0.668868
25	H	-3.605443	-2.098206	-2.491232
26	H	-5.750487	-2.555002	-3.611744
27	H	-7.718633	-1.072765	-3.213292
28	H	-7.512449	0.853199	-1.647833
29	H	-5.374240	1.302156	-0.499777
30	H	-2.922863	2.836516	-0.984061
31	H	-3.496235	4.711454	0.513552
32	H	-3.805016	1.974895	3.831027
33	H	-3.254076	0.092727	2.332503
34	Co	-1.053306	0.272431	-2.156957
35	C	-0.977363	-1.478143	-2.538955
36	O	-0.880389	-2.611889	-2.782230
37	C	-2.137799	1.280884	-3.161197
38	O	-2.855706	1.865255	-3.860957
39	Co	1.519774	0.795374	-2.106261
40	C	2.597331	-0.302415	-3.019253
41	O	3.341526	-0.907951	-3.673445
42	C	-0.062342	1.425202	-0.996056
43	O	-0.181241	2.214189	-0.126773
44	C	0.308625	0.589758	-3.556841
45	O	0.313884	0.601340	-4.737659
46	C	1.987939	2.471723	-2.492957
47	O	2.329783	3.528914	-2.832445
48	P	2.786586	0.311668	-0.237362
49	C	4.610577	0.376597	-0.583463
50	C	2.619918	1.391455	1.246499
51	C	2.598403	-1.393041	0.451486
52	C	2.173162	-2.421961	-0.402319
53	C	2.142265	-3.745883	0.040728

54	C	2.526858	-4.057673	1.346299
55	C	2.927154	-3.036935	2.214409
56	C	2.962932	-1.712856	1.771217
57	C	5.126013	1.413983	-1.378562
58	C	6.498260	1.513552	-1.620224
59	C	7.377444	0.568547	-1.087929
60	C	6.873604	-0.475579	-0.310138
61	C	5.503348	-0.571738	-0.059175
62	C	1.528277	1.214813	2.111935
63	C	1.332549	2.073916	3.192405
64	C	2.215907	3.132709	3.416850
65	C	3.299591	3.321480	2.557163
66	C	3.502709	2.457061	1.479161
67	H	1.859411	-2.184536	-1.417559
68	H	1.808202	-4.529808	-0.638521
69	H	2.505526	-5.092491	1.689351
70	H	3.220339	-3.270969	3.238363
71	H	3.287924	-0.926499	2.451231
72	H	0.819986	0.404811	1.943516
73	H	0.476699	1.920491	3.849833
74	H	2.058348	3.808758	4.257557
75	H	3.995464	4.144454	2.724141
76	H	4.358905	2.614330	0.825526
77	H	4.456550	2.149915	-1.819714
78	H	6.876250	2.331414	-2.234419
79	H	7.548212	-1.225732	0.103879
80	H	5.135436	-1.399710	0.543530
81	H	8.447833	0.641940	-1.282030
82	H	-3.933666	4.298617	2.933111

Complex **3B**

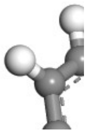

Charge: 0

ATOM		X	Y	Z
1	P	-2.104380	-2.100038	0.493555
2	C	-3.774647	-2.465692	1.190995
3	C	-2.264453	-2.489837	-1.302442
4	C	-1.087361	-3.524146	1.080094
5	C	-0.162264	-3.339150	2.115376
6	C	0.592972	-4.413462	2.593395
7	C	0.432212	-5.683703	2.038565
8	C	-0.487407	-5.878185	1.003498
9	C	-1.243720	-4.807554	0.525321
10	C	-4.831820	-1.574458	0.933233
11	C	-6.095168	-1.793306	1.483916
12	C	-6.316677	-2.891062	2.319937
13	C	-5.268842	-3.770833	2.594905
14	C	-4.006484	-3.564267	2.031628
15	C	-1.084392	-2.687499	-2.039401
16	C	-1.138646	-2.967603	-3.403521
17	C	-2.371533	-3.047460	-4.056404
18	C	-3.548356	-2.851828	-3.332587
19	C	-3.497962	-2.573395	-1.963816
20	H	-0.018768	-2.343632	2.533810
21	H	1.313669	-4.249705	3.394791
22	H	1.027699	-6.520717	2.404049
23	H	-0.615692	-6.867557	0.562824
24	H	-1.953035	-4.968735	-0.287027
25	H	-0.114663	-2.639249	-1.545573

26	H	-0.211920	-3.122829	-3.956356
27	H	-2.413577	-3.265059	-5.123730
28	H	-4.516553	-2.922476	-3.829213
29	H	-4.428039	-2.444261	-1.412981
30	H	-4.669056	-0.697280	0.308713
31	H	-6.903876	-1.093210	1.272317
32	H	-5.429408	-4.623114	3.255553
33	H	-3.199183	-4.257830	2.260835
34	Co	-1.406866	0.003810	1.132283
35	C	-1.892509	-0.393060	2.818330
36	O	-2.242152	-0.582729	3.907043
37	C	-2.661192	1.123725	0.548936
38	O	-3.492911	1.868853	0.230397
39	Co	1.114842	0.361594	0.898506
40	C	1.937527	-1.026510	0.121792
41	O	2.465192	-1.953010	-0.336935
42	C	-0.208750	1.321191	1.927556
43	O	-0.301383	2.200949	2.714971
44	C	-0.316284	0.191897	-0.446317
45	O	-0.407604	0.209758	-1.627215
46	C	1.954993	0.087419	2.455747
47	O	2.474204	-0.149122	3.466684
48	P	2.039590	2.190653	-0.086284
49	C	0.872365	3.385606	-0.877324
50	C	3.073264	3.250724	1.019016
51	C	3.225068	1.815347	-1.457852
52	C	2.892433	0.829412	-2.401953
53	C	3.763484	0.532787	-3.452130
54	C	4.979993	1.209539	-3.571537
55	C	5.316440	2.193081	-2.639809
56	C	4.443642	2.498770	-1.592220
57	C	-0.115705	4.001149	-0.086498
58	C	-1.044525	4.869848	-0.659889
59	C	-1.011756	5.131701	-2.031895

60	C	-0.039323	4.521169	-2.824781
61	C	0.897890	3.655757	-2.253715
62	C	4.037384	2.624948	1.827344
63	C	4.861671	3.379817	2.662533
64	C	4.726238	4.770260	2.712501
65	C	3.775214	5.400831	1.908497
66	C	2.957069	4.647648	1.061226
67	H	1.940860	0.303833	-2.330822
68	H	3.487735	-0.231890	-4.178624
69	H	5.663003	0.972429	-4.387653
70	H	6.262448	2.729068	-2.723665
71	H	4.719856	3.269018	-0.873747
72	H	4.154981	1.541905	1.796809
73	H	5.605200	2.878897	3.283564
74	H	3.663839	6.484913	1.941440
75	H	2.226213	5.153696	0.432158
76	H	-0.160841	3.811815	0.983950
77	H	-1.801741	5.336690	-0.029193
78	H	-1.743444	5.804421	-2.479619
79	H	-0.005483	4.714886	-3.897361
80	H	1.650499	3.190524	-2.888129
81	H	-7.299437	-3.053635	2.763283
82	H	5.360101	5.358748	3.376345

Complex **3C**

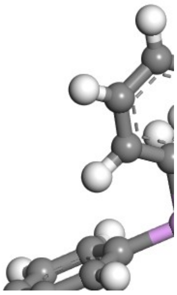

Charge: 0

ATOM		X	Y	Z
1	P	-2.452452	-0.800969	-1.395904
2	C	-4.142592	-1.035983	-0.676540
3	C	-2.718596	-1.075666	-3.200615
4	C	-1.575336	-2.345452	-0.896068
5	C	-0.724853	-2.315170	0.217228
6	C	-0.059619	-3.468487	0.638285
7	C	-0.233354	-4.666496	-0.056306
8	C	-1.082701	-4.709323	-1.165448
9	C	-1.753021	-3.558670	-1.583415
10	C	-5.055594	0.032399	-0.706543
11	C	-6.341730	-0.115278	-0.185042
12	C	-6.730938	-1.325845	0.392466
13	C	-5.824431	-2.385667	0.446061
14	C	-4.540025	-2.245093	-0.085236
15	C	-1.623119	-1.446711	-3.999561
16	C	-1.778795	-1.634283	-5.372020
17	C	-3.025657	-1.443025	-5.971549
18	C	-4.117162	-1.066396	-5.187743
19	C	-3.966663	-0.881196	-3.811058
20	H	-0.560354	-1.370756	0.734274
21	H	0.612357	-3.423716	1.495678
22	H	0.298297	-5.564559	0.258906
23	H	-1.224719	-5.643008	-1.710139
24	H	-2.409056	-3.602790	-2.452389
25	H	-0.643297	-1.603467	-3.550916
26	H	-0.917569	-1.921994	-5.975084
27	H	-3.145279	-1.584536	-7.045283
28	H	-5.095317	-0.915137	-5.645517
29	H	-4.832961	-0.599763	-3.213956
30	H	-4.762305	0.991540	-1.130771
31	H	-7.034985	0.725074	-0.219767
32	H	-6.113157	-3.330307	0.907691
33	H	-3.845922	-3.082298	-0.030763

34	Co	-1.531237	1.208426	-0.768438
35	C	-2.262344	1.024989	0.863210
36	O	-2.808506	0.968752	1.885811
37	C	-2.575581	2.434435	-1.525099
38	O	-3.281339	3.237297	-1.977388
39	Co	1.050550	1.296852	-0.819935
40	C	1.561356	-0.354306	-1.311099
41	O	1.875863	-1.388795	-1.731934
42	C	-0.199109	2.470608	0.017477
43	O	-0.288600	3.466919	0.650287
44	C	-0.323227	1.157933	-2.245901
45	O	-0.284772	1.127528	-3.426656
46	C	2.006306	2.432001	-1.816402
47	O	2.647930	3.080053	-2.532187
48	P	2.357526	1.321131	1.072090
49	C	3.591007	2.693744	1.039157
50	C	1.588148	1.552802	2.732511
51	C	3.389832	-0.192232	1.306327
52	C	4.298498	-0.568010	0.300978
53	C	5.039025	-1.744136	0.420848
54	C	4.872263	-2.572149	1.534884
55	C	3.971125	-2.207816	2.536432
56	C	3.238949	-1.022260	2.427017
57	C	3.130569	3.981164	0.712658
58	C	4.013968	5.059661	0.687952
59	C	5.366314	4.867924	0.983901
60	C	5.830053	3.593201	1.313031
61	C	4.947467	2.510137	1.345414
62	C	0.388456	0.889723	3.027102
63	C	-0.226991	1.045777	4.269647
64	C	0.355061	1.867116	5.237964
65	C	1.553717	2.526979	4.956236
66	C	2.166706	2.375468	3.711263
67	H	4.430309	0.058705	-0.581733

68	H	5.738697	-2.021217	-0.367878
69	H	5.443970	-3.496143	1.621520
70	H	3.832291	-2.843798	3.411260
71	H	2.548824	-0.741682	3.221300
72	H	-0.077612	0.263108	2.268970
73	H	-1.172030	0.541029	4.467886
74	H	2.012512	3.172830	5.705585
75	H	3.092390	2.908069	3.501502
76	H	2.076776	4.144876	0.487624
77	H	3.643693	6.051459	0.427359
78	H	6.055023	5.711737	0.961054
79	H	6.882584	3.435573	1.550403
80	H	5.319171	1.522931	1.615454
81	H	-7.733321	-1.439589	0.805444
82	H	-0.131364	2.004263	6.204151

Complex **4** [$HCo(CO)_3P$]

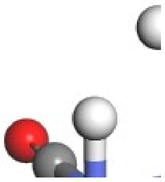

Charge: 0

ATOM		X	Y	Z
1	P	-0.472172	0.248459	-0.316297
2	C	-0.805770	-1.202308	0.771428
3	C	0.917447	-0.353796	-1.373481
4	C	0.377891	1.431823	0.817757
5	C	-0.401537	2.367877	1.513737
6	C	0.192196	3.260264	2.407552

7	C	1.575432	3.239725	2.601810
8	C	2.360842	2.319035	1.905127
9	C	1.767499	1.416486	1.019518
10	C	-1.446306	-2.316269	0.202197
11	C	-1.739589	-3.437004	0.977171
12	C	-1.415733	-3.452487	2.337110
13	C	-0.790526	-2.346189	2.912656
14	C	-0.479344	-1.226780	2.134504
15	C	1.301911	0.409326	-2.487034
16	C	2.364362	0.005433	-3.294374
17	C	3.051961	-1.176462	-3.008706
18	C	2.678250	-1.943419	-1.904608
19	C	1.620894	-1.534291	-1.087336
20	H	-1.478290	2.400965	1.346792
21	H	-0.424949	3.982873	2.941828
22	H	2.041154	3.945260	3.289670
23	H	3.441796	2.302385	2.048485
24	H	2.391459	0.702675	0.482328
25	H	0.765300	1.326698	-2.728536
26	H	2.646239	0.608799	-4.156866
27	H	3.870699	-1.502190	-3.649714
28	H	3.205196	-2.870103	-1.678343
29	H	1.341579	-2.142120	-0.227542
30	H	-1.717462	-2.305020	-0.854321
31	H	-2.231555	-4.296184	0.520904
32	H	-1.655977	-4.323463	2.946269
33	H	-0.541306	-2.348380	3.974339
34	H	0.015143	-0.371844	2.594361
35	Co	-2.254337	1.008244	-1.407570
36	H	-1.176772	1.527073	-2.291729
37	C	-3.524091	0.463382	-0.276990
38	O	-4.373866	0.135671	0.438820
39	C	-2.612890	2.744936	-1.388830
40	O	-2.825151	3.885464	-1.437066

41	C	-2.653706	0.012194	-2.829966
42	O	-2.905909	-0.596755	-3.785065

Complex **6** [$HCo(CO)_2P_2$]

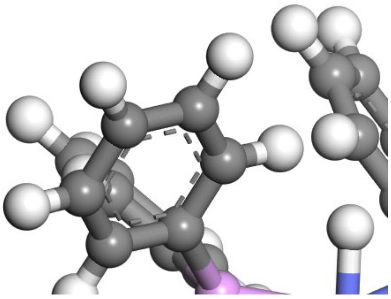

Charge: 0

ATOM		X	Y	Z
1	P	1.094707	-1.611324	0.406018
2	C	0.849659	-2.996010	1.607884
3	C	2.430780	-2.294206	-0.685974
4	C	2.036312	-0.376885	1.417745
5	C	1.304858	0.596201	2.113651
6	C	1.949899	1.554916	2.895491
7	C	3.344284	1.560116	2.981638
8	C	4.085053	0.598191	2.290475
9	C	3.436576	-0.365390	1.512739
10	C	0.181956	-4.148307	1.157218
11	C	-0.041509	-5.221655	2.019245
12	C	0.381594	-5.153783	3.350376
13	C	1.033942	-4.008868	3.810097
14	C	1.272033	-2.936766	2.943433
15	C	2.708746	-1.663425	-1.908691
16	C	3.735988	-2.122730	-2.734208
17	C	4.491738	-3.237532	-2.363052
18	C	4.213645	-3.884812	-1.157886
19	C	3.196299	-3.414542	-0.322285
20	H	0.220058	0.605866	2.016991

21	H	1.363756	2.313223	3.415316
22	H	3.852772	2.316169	3.580626
23	H	5.174030	0.595977	2.351865
24	H	4.025705	-1.109285	0.977032
25	H	2.102074	-0.815932	-2.226490
26	H	3.934687	-1.617240	-3.678951
27	H	5.285840	-3.603854	-3.013717
28	H	4.789155	-4.762726	-0.863209
29	H	2.996581	-3.927755	0.617364
30	H	-0.164647	-4.203471	0.124993
31	H	-0.556808	-6.109414	1.651716
32	H	0.197696	-5.988647	4.027017
33	H	1.362239	-3.943737	4.847959
34	H	1.788323	-2.051858	3.313340
35	Co	-0.739969	-0.999216	-0.643000
36	H	0.232839	-0.304773	-1.543478
37	C	-1.925349	-1.614810	0.519769
38	O	-2.728514	-2.026301	1.253744
39	C	-1.024654	-2.088102	-2.006780
40	O	-1.192829	-2.832851	-2.888054
41	P	-1.515693	1.045953	-0.773501
42	C	-2.979153	1.454977	0.286665
43	C	-0.366856	2.454320	-0.397801
44	C	-2.135844	1.503711	-2.461451
45	C	-2.776982	0.522655	-3.233496
46	C	-3.277953	0.828581	-4.500022
47	C	-3.139494	2.119753	-5.014416
48	C	-2.504654	3.103486	-4.252949
49	C	-2.006900	2.799719	-2.982935
50	C	-2.852980	1.353390	1.682714
51	C	-3.932431	1.628868	2.520755
52	C	-5.167879	1.990193	1.975732
53	C	-5.310564	2.075138	0.591000
54	C	-4.223262	1.816386	-0.249365

55	C	0.977988	2.357883	-0.785843
56	C	1.873327	3.397093	-0.530149
57	C	1.439586	4.548910	0.131060
58	C	0.104590	4.656410	0.525578
59	C	-0.794655	3.619052	0.260103
60	H	-2.876364	-0.488792	-2.842688
61	H	-3.766288	0.050978	-5.087874
62	H	-3.519654	2.355476	-6.008408
63	H	-2.394435	4.115176	-4.643674
64	H	-1.512493	3.575940	-2.399772
65	H	1.330067	1.449731	-1.273926
66	H	2.916675	3.297037	-0.830008
67	H	2.141023	5.356967	0.342093
68	H	-0.244506	5.552747	1.039348
69	H	-1.834884	3.716791	0.568509
70	H	-1.903435	1.047325	2.121806
71	H	-3.808404	1.551368	3.601007
72	H	-6.016029	2.194528	2.628765
73	H	-6.270286	2.349849	0.152971
74	H	-4.350721	1.893945	-1.327791

12 References

108. Fulmer, G. R.; Miller, A. J. M.; Sherden, N. H.; Gottlieb, H. E.; Nudelman, A.; Stoltz, B. M.; Bercaw, J. E.; Goldberg, K. I., NMR Chemical Shifts of Trace Impurities: Common Laboratory Solvents, Organics, and Gases in Deuterated Solvents Relevant to the Organometallic Chemist. *Organometallics* **2010,** *29* (9), 2176-2179.DOI: 10.1021/om100106e
109. Férey, G.; Mellot-Draznieks, C.; Serre, C.; Millange, F.; Dutour, J.; Surblé, S.; Margiolaki, I., A Chromium Terephthalate-Based Solid with Unusually Large Pore Volumes and Surface Area. *Science* **2005,** *309* (5743), 2040-2042.DOI: 10.1126/science.1116275
110. Park, H. D.; Dinca, M.; Roman-Leshkov, Y., Heterogeneous Epoxide Carbonylation by Cooperative Ion-Pair Catalysis in $Co(CO)_4^-$ Incorporated Cr-MIL-101. *ACS Cent. Sci.* **2017,** *3* (5), 444-448.DOI: 10.1021/acscentsci.7b00075
111. Mao, C.; Kudla, R. A.; Zuo, F.; Zhao, X.; Mueller, L. J.; Bu, X.; Feng, P., Anion stripping as a general method to create cationic porous framework with mobile anions. *J. Am. Chem. Soc.* **2014,** *136* (21), 7579-82.DOI: 10.1021/ja5030723
112. Carretero-Cerdán, A.; Carrasco, S.; Sanz-Marco, A.; Jaworski, A.; Martín-Matute, B., One-step microwave-assisted synthesis of amino-functionalized chromium(III) terephthalate MIL-101-NH_2. *Materials Today Chem.* **2023,** *31*, 101618.DOI: 10.1016/j.mtchem.2023.101618
113. Edgell, W. F.; Lyford, J. I. V., Preparation of sodium cobalt tetracarbonyl. *Inorg. Chem.* **1970,** *9* (8), 1932-1933.DOI: 10.1021/ic50090a032
114. Hieber, W.; Freyer, W., Über triphenylphosphinhaltige Kobaltcarbonyle. *Chemische Berichte* **2006,** *91* (6), 1230-1234.DOI: 10.1002/cber.19580910618
115. Amann, A.; Ourisson, G.; Luu, B., A Novel Stereospecific Synthesis of 22-Hydroxylated Triterpenes and Steroids: Syntheses of 22R-Hydroxylanosterol and 22R-Hydroxydesmosterol. *Synthesis* **1987,** *1987*, 696 - 700, DOI: 10.1055/s-1987-28048
116. Metalary. https://www.metalary.com (accessed 02/2025).
117. Wood, C. D.; Garrou, P. E., Formate formation during $Co_2(CO)_8/PR_3$-catalyzed hydroformylation. *Organometallics* **1984,** *3* (1), 170-174.DOI: 10.1021/om00079a030
118. Genna, D. T.; Pfund, L. Y.; Samblanet, D. C.; Wong-Foy, A. G.; Matzger, A. J.; Sanford, M. S., Rhodium Hydrogenation Catalysts Supported in Metal Organic Frameworks: Influence of the Framework on Catalytic Activity and Selectivity. *ACS Catal.* **2016,** *6* (6), 3569-3574.DOI: 10.1021/acscatal.6b00404
119. Delley, B., From molecules to solids with the DMol3 approach. *J. Chem. Phys.* **2000,** *113* (18), 7756-7764.DOI: 10.1063/1.1316015
120. Perdew, J. P.; Burke, K.; Ernzerhof, M., Generalized Gradient Approximation Made Simple. *Phys. Rev. Lett.* **1996,** *77* (18), 3865-3868.DOI: 10.1103/PhysRevLett.77.3865
